# Tunable Photocatalytic Selectivity by Altering the Active Center Microenvironment of an Organic Polymer Photocatalyst

**DOI:** 10.1021/acsami.2c17607

**Published:** 2023-01-03

**Authors:** Julian Heuer, Thomas Kuckhoff, Rong Li, Katharina Landfester, Calum T. J. Ferguson

**Affiliations:** † Max Planck Institute for Polymer Research, Ackermannweg 10, Mainz55128, Germany; ‡ School of Chemistry, University of Birmingham, Edgbaston, BirminghamB15 2TT, United Kingdom

**Keywords:** photocatalysis, block copolymer, nanoparticles, selectivity, hydrophilicity

## Abstract

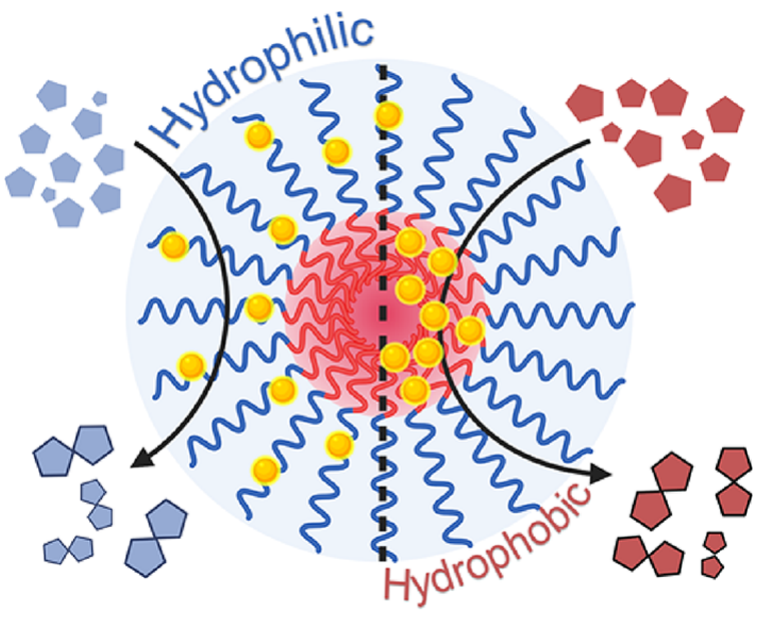

The favored production of one product over another is a major challenge in synthetic chemistry, reducing the formation of byproducts and enhancing atom efficacy. The formation of catalytic species that have differing reactivities based on the substrate being converted, has been targeted to selectively control reactions. Here, we report the production of photocatalytic self-assembled amphiphilic polymers, with either hydrophilic or hydrophobic microenvironments at the reactive center. Benzothiadiazole-based photocatalysts were polymerized into either the hydrophilic or the hydrophobic compartment of a diblock copolymer by RAFT polymerization. The difference in the reactivity of each microenvironment was dictated by the physical properties of the substrate. Stark differences in reactivity were observed for polar substrates, where a hydrophilic microenvironment was favored. Conversely, both microenvironments performed similarly for very hydrophobic substrates, showing that reagent partitioning is not the only factor that drives photocatalytic conversion. Furthermore, the use of secondary swelling solvents allowed an additional reagent exchange between the continuous phase and the heterogeneous photocatalyst, resulting in a significant 5-fold increase in conversion for a radical carbon–carbon coupling.

## Introduction

Catalysis has become one of the central anchors of organic chemistry, with evermore sophisticated catalytic systems reported every year.^1,2^ Much of this progress has been in the pursuit of selective catalysis. For the preferential generation of a specific stereoisomer at increased reaction rates, enantioselective catalysts are targeted.^3−6^ However, many of these synthetic catalysts still produce undesired side products, due to impure starting materials, functional group interference, or insufficient substrate selectivity.^7,8^ The broad variety of products leads to increased separation and purification costs as well as poor selectivity and therefore reduced sustainability.^9^

Enzymes are nature’s catalysts, exhibiting inherent selectivity for a specific substrate and the formation of its desired corresponding product. From a pool of substrates, a single species is converted into a specific product with maximum selectivity.^10,11^ This selectivity stems from the variation of two key components within these biomaterials, the active center and the macromolecular structure. The three-dimensional macromolecular structure selects the substrate based on its polarity and electronic configuration, while the active center within the enzyme determines the spatial conformation of the substrate through interaction with protruding local amino acid residues. To date, much of the selective catalysis research has aimed to miniaturize and reproduce a synthetic enzymatic active center.^12−14^ However, the macromolecular nature of enzymes plays an essential role in substrate specificity by retaining different functionalities or shielding the active site from water.^12−17^ Recent advances in macromolecular chemistry have enabled intricate catalytic sites to be produced, showing increased substrate selectivity. Yet, the scalability of macromolecular catalytic systems can often be a major challenge due to the complexity of the catalyst and challenging recovery processes.^12,17^

To mimic enzymatic substrate specificity and selectivity, the development of synthetic macromolecular catalytic systems should be targeted, where the macromolecular structure induces a particular reaction selectivity. This polymeric material should contain catalytic centers comparable to enzymatic active sites, where transformations take place. The selected reactive sites should enable the catalysis for a broad range of reactions to create a versatile catalytic center. By controlling the microenvironment around the catalytic centers, the spatial proximity of substrate to catalyst could be controlled, potentially reducing side reactions and therefore inducing selectivity. Additionally, the heterogeneous catalytic system should be readily dispersible or soluble in green solvents such as water while being capable of processing a broad range of reagents with varying polarities. Finally, the catalytic material should be reusable and have the potential for future scalability.

Over the past decade, photocatalysis has proven to be a reliable and valuable synthetic tool, with numerous reported examples of photocatalytic variants for classical catalytic reactions.^18−22^ Through harnessing of visible light, photocatalysis facilitates a large array of different chemical reactions.^18^ Additionally, changes within the photocatalysts’ molecular structure allow enhanced control over the desired reaction through adjustable physicochemical properties, such as redox potential and lifetime.^19−22^ Embedding photocatalysts into macro- or supramolecular structures offers additional opportunities for tunability through morphological control, therefore enabling photocatalyst compartmentalization and enhanced substrate accessability.^23−26^ Homogeneous, nanoscale distributions of photocatalysts within polymersomes,^25^ micelles,^26^ nanoparticles,^27^ and on surfaces^28^ were reported, but the desired implementation of selectivity concepts into these structures is still a subject of on-going research.^23,24,29^

Its versatility has already been demonstrated in a variety of chemical reactions such as water splitting,^30,31^ CO_2_ reduction,^32,33^ organic pollutant degradation,^34^ C–C coupling reactions,^35−37^ C=C bond cleavage,^38−40^ metal reduction,^41^ oxidative coupling of amines,^42^ trifluoromethylation of arenes,^43^ oxidation of sulfides,^42^ free radical polymerizations,^44−46^ dehalogenation of halo ketones,^47^ photodynamic therapy,^48−50^ heterocycle formation,^51^ bacterial treatment,^52^ and enantioselective alpha-alkylation.^53^ However, the scope in selectivity has been limited to date with restricted control given by the structural properties of the photocatalyst. Some selectivity has been achieved due to the inherent selectivity within the photocatalytic mechanism.^54,55^

**Scheme 1:**
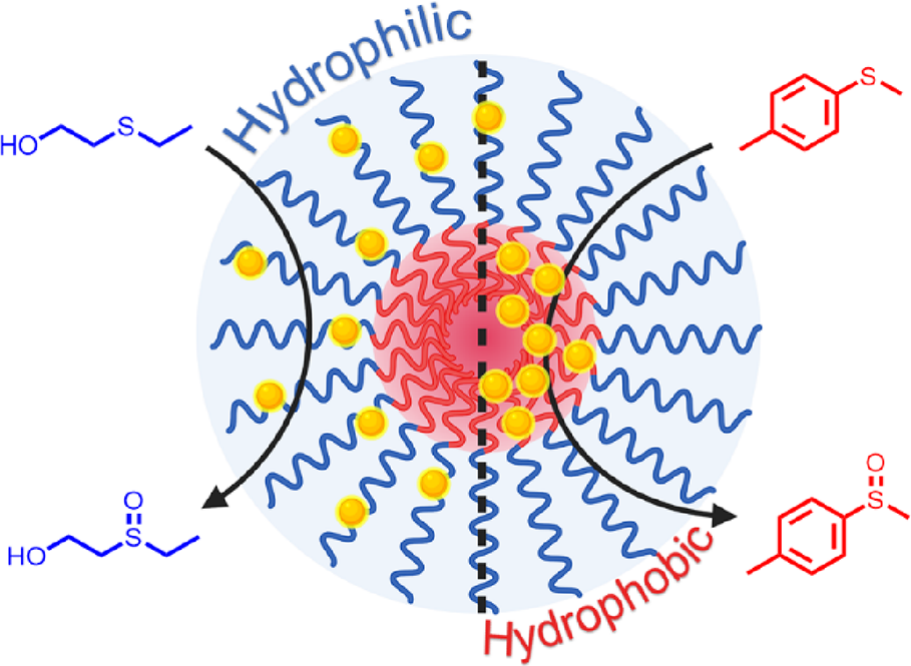
Polymeric Nanoparticles Modified with Covalently Bound Photocatalysts at Different Chain Positions (Yellow Dots), Showing Higher Substrate Conversions Aligning with Respective Substrate Hydrophilicity Blue = hydrophilic, red = hydrophobic.

Here, we report photocatalyst-embedded polymeric micelles, where the location of the photocatalytic center can be tuned (Scheme 1). The variation from a hydrophilic to a hydrophobic microenvironment of the photocatalytic center induces an increase in selectivity and differing reaction rates depending on the polarity of the substrate. This macromolecular photocatalytic system was synthesized using reversible addition-fragmentation chain-transfer (RAFT)-mediated polymerization induced self-assembly (PISA), where a photocatalytic monomer could be readily polymerized into the polymer at a specified location.^56,57^ Previously, photocatalytic self-assembled particles have been reported, where the inclusion of the photocatalytic moiety has been concentrated in either the hydrophilic or hydrophobic portion. Conflicting reports and confusion seem to have arisen over whether inclusion into either phase is preferential.^56−60^ Here, we elucidate some of the dynamics within the photocatalytic material by investigating three model reactions and aim to use these dynamics to invoke preferential reactivity in these reactions. Lastly, the microenvironment of the photocatalyst was further modified through the introduction of a secondary solvent that preferentially swells the self-assembled material, enhancing reagent exchange and reaction conversion.

## Results and Discussion

**Figure 1:**
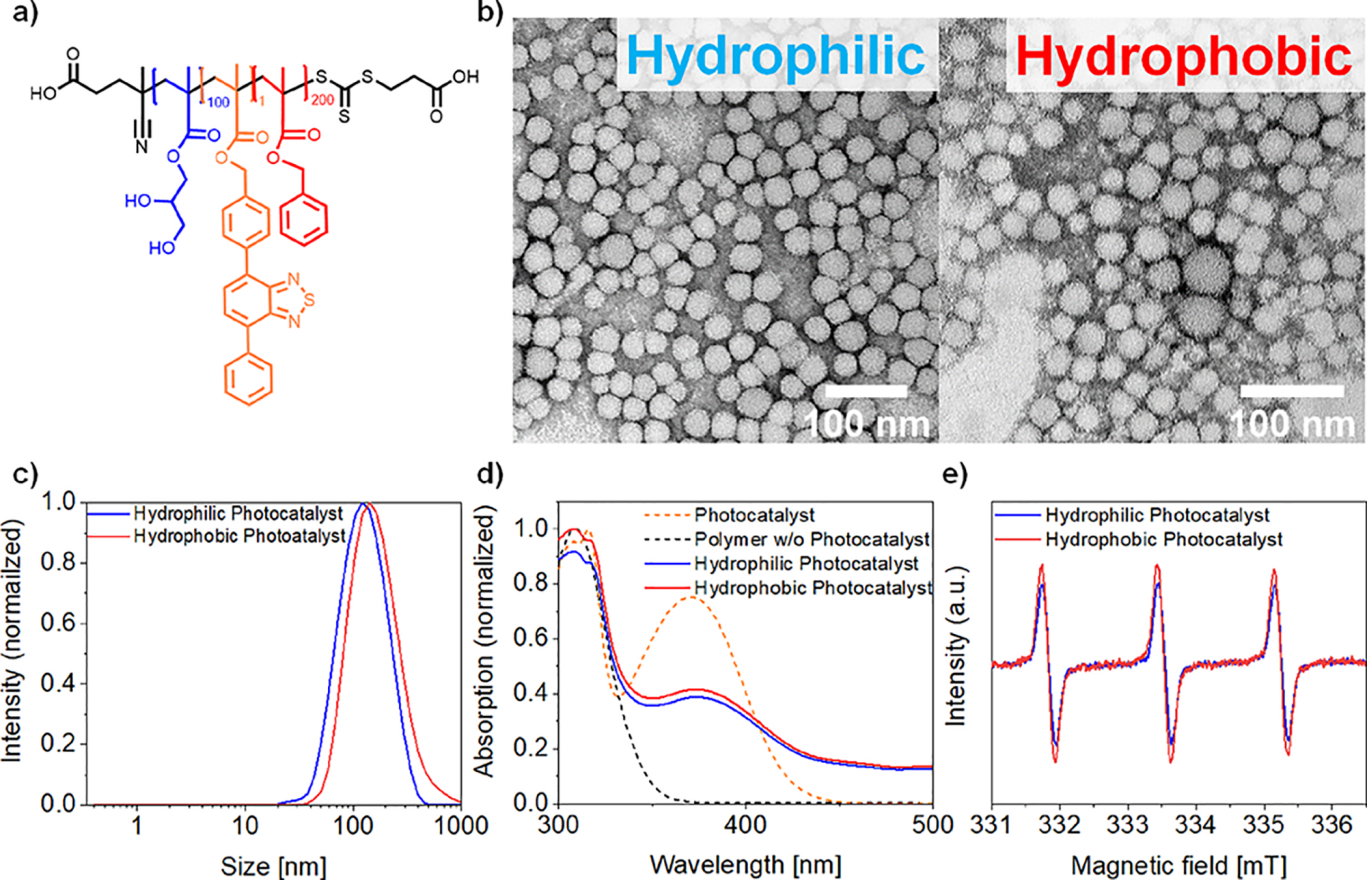
Characterization of the synthesized photocatalytic nanoparticles. (a) Chemical structure of the designed block copolymer. (b) Transmission electron microscopy (TEM) images of the hydrophilic and hydrophobic nanoparticles. (c) Dynamic light scattering (DLS) spectra for both nanoparticle systems in H_2_O. (d) UV/VIS-absorption spectrum of both nanoparticles, showing similar intensities at 373 nm in DMSO. (e) Electron paramagnetic resonance spectra of both nanoparticle systems after 10 min of 465 nm LED irradiation in H_2_O with TEMPO trapping.

Polymer-based, photocatalytic nanoparticles were synthesized by RAFT-PISA (Figure 1a). First, a macromolecular chain transfer agent (mCTA) was prepared by reacting the water-soluble trithiocarbonate 4-((((2-carboxyethyl)thio)carbonothioyl)thio)-4-cyanopentanoic acid (CCCP) with 100 equiv of the hydrophilic monomer glyceryl monomethacrylate (GMA). The required amphiphilicity of the polymer was achieved by blocking off with 200 equiv of the hydrophobic monomer benzyl methacrylate (BzMA), which resulted in dispersion-based polymerization-induced self-assembly (PISA) into homogeneous nanoparticles. Depending on the addition of the photocatalyst 4-(7-phenylbenzo[1,2,5]thiadiazol-4-yl)benzyl methacrylate (BTPMA) during either the hydrophilic mCTA synthesis or during the hydrophobic blocking off, its position within the polymer chain was controlled. For the incorporation of the photocatalyst in both positions colloidal stable, homogeneous, spherical, and polymeric nanoparticles of discrete size and solid content were formed (Figure 1b).^61−63^

Characterization by ^1^H-NMR spectroscopy of the amphiphilic block copolymer systems revealed similar conversion rates and photocatalyst incorporation for the hydrophilic and hydrophobic located photocatalyst polymer (Figures S3–S5). The small differences in conversion rate may be due to the prior copolymerization of the hydrophobic photocatalyst into the hydrophilic block, disfavoring the hydrophilic steric stabilization. Gel permeation chromatography (GPC) analysis gave identical elution times and a polydispersity of 1.5 for both photocatalytic systems, which is higher than typically achieved for RAFT-mediated PISA but probably arising from the inclusion of the photocatalytic monomer (Figure S6). These two techniques allowed the determination of monomer ratios for the polymers, which are P(GMA)_96_-*b*-P(BzMA)_210_-BTPMA_1,08_ for the hydrophilic photocatalyst and P(GMA)_99_-*b*-P(BzMA)_217_-BTPMA_1,07_ for the hydrophobic photocatalyst (Figures S3–S5).

Additionally, negligible differences were observed between the two species after FTIR analysis (Figures S8–S10). The structural morphology and size of the self-assembled polymers were analyzed by transmission electron microscopy (TEM) and dynamic light scattering (DLS) measurements, indicating homogeneous nanoparticle formation with monomodal size distributions. Average size diameters of 40 ± 5 nm spherical nanoparticles were obtained for both species (Figure 1b). DLS analysis gave an average hydrodynamic radius of 140 ± 10 nm (Figure 1c). The DLS measurement revealed a marginally higher hydrodynamic diameter for the hydrophobic located photocatalyst, which is attributed to the slightly higher BzMA conversion within the nanoparticle formation process. The size difference between TEM and DLS may be due to morphological changes upon drying of the nanoparticles for TEM analysis.

UV–Vis absorption spectroscopy measurements of both polymeric nanoparticles were analyzed in dimethyl sulfoxide (DMSO) for disassembly of nanoparticles, showing a small difference of 3% in absorption at the characteristic maximum absorption wavelength (373 nm) of the photocatalyst within the UV-A region (Figure 1d). A slightly higher difference of 7% was observed at the overlapping absorption wavelength of BzMA and the energetically higher UV-B absorption peak of the photocatalyst at around 307 nm. These modest differences suggest a minimal deviation in incorporation of the BTPMA and BzMA, in alignment with NMR analysis (Figures S3–S5). An intense emission peak at around 500 nm was observed for both polymers in DMSO, which is attributed to the expected maximum in the photocatalyst’s emission spectrum (Figures S12 and S13). The generation of singlet oxygen was monitored by electron paramagnetic resonance (EPR) spectroscopy, where the trapping agent 2,2,6,6-tetramethylpiperidinel (TEMP) was used to analyze singlet oxygen generation upon irradiation with 465 nm LEDs (Figure 1e). In both samples, the generation of singlet oxygen was detected; however, the hydrophobic located photocatalyst showed a slightly higher signal. Considering the lipophilicity of the trapping agent, a possible accumulation of the trapping agent around the hydrophobic located photocatalyst may explain an increased formation of TEMPO compared to the hydrophilic located photocatalyst.

**Figure 2:**
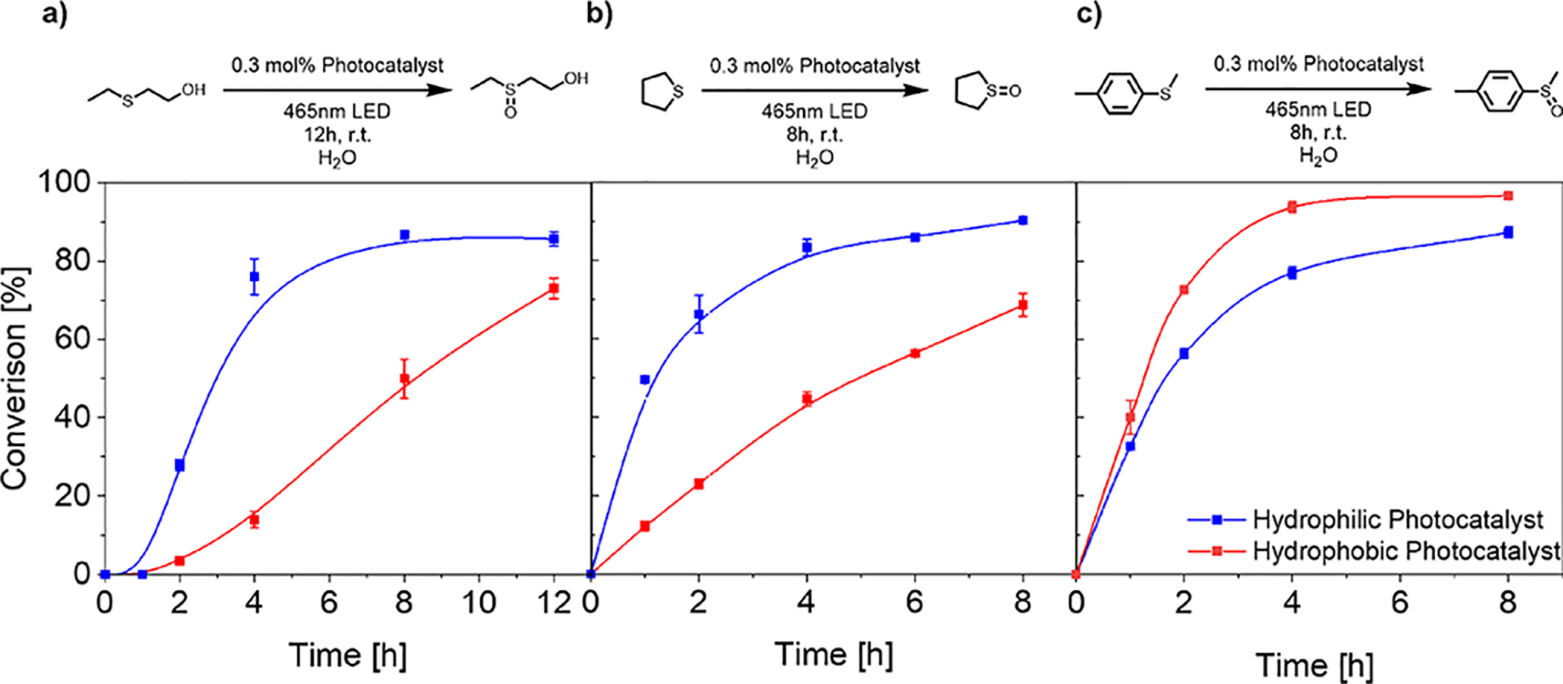
Kinetic profiles of the sulfide oxidation reaction performed by both polymeric, photocatalytic nanoparticles. Ranked from left to right from the most hydrophilic to the most hydrophobic substrates, underlining the dependence on substrate hydrophilicity. (a) Oxidation of 2-(ethylsulfanyl)ethanol. (b) Oxidation of tetrahydrothiophene. (c) Oxidation of methyl-*p*-tolylsulfide. Conditions: Sulfide species (19.96 μmol), photocatalyst polymer (0.096 μmol, 0.3 mol % photocatalyst), dispersed in H_2_O (2 mL, sonicated 20 min), 15 °C, 7.14 W, 465 nm, 16 h. The conversion was calculated from GC measurements by comparison of peak area intensity.

To investigate the impact of the microenvironment on the photocatalytic activity, the nanoparticles’ reactivity towards sulfide oxidation was evaluated. Functional group variation with consideration of their structural and electronic properties compiled a series of substrates with varying log *P* values. Log *P* values give an approximation of membrane permeability and their solvation by analyzing the compounds’ lipophilicity, allowing an estimation of substrate location within the nanoparticle system (Figure S15).^64^Figure 2 shows the kinetic profiles of the sulfide oxidation reaction for three thioethers (increasing in hydrophobicity from left to right) into sulfoxides under identical reaction conditions. A clear difference in the conversion of 2-(ethylthio)ethan-1-ol (log *P* = 0.63), the most hydrophilic substrate, between the hydrophilic and hydrophobic-based photocatalyst was observed (Figure 2a). Specifically, a faster rate of conversion with a maximum difference of 62% after 4 h was observed for the hydrophilic-based photocatalyst compared to the hydrophobic one, with the hydrophilic-located photocatalyst plateauing at 80% after 4 h, while the hydrophobic-located photocatalyst showed a linear progression, reaching 72% after 12 h (product log *P* = −1.32). Conversely, the hydrophobic-based catalyst had only converted around 70% of the hydrophobic substrate after 12 h. A similar trend was observed for tetrahydrothiophene (log *P* = 1.13) (Figure 2b), where the conversion was again faster in the hydrophilic system than in the hydrophobic one (product log *P* = −0.83). Interestingly, in the case of the most hydrophobic substrate methyl *p*-tolyl sulfide (log *P* = 2.96) (Figure 2c), the photocatalyst in the hydrophobic microenvironment outperformed the hydrophilic equivalent. Here, the hydrophobic located photocatalyst showed a faster conversion with a maximum difference of 16% after 4 h compared to the hydrophilic located photocatalyst (product log *P* = 1.18). Interestingly, a linear relationship is observed between the substrate lipophilicity (log *P*) and the substrate conversion difference after 4 h reaction time (Figure S15). Additionally, recyclability of the polymeric, photocatalytic nanoparticles was demonstrated, showing high performance of the recycled material at 95% conversion for 4-(methylthio)toluene after 4 cycles (Figure S16).

The sulfide oxidation reaction mechanism is initiated by a nucleophilic attack of thioethers by the reactive oxygen species, such as singlet oxygen, and is therefore highly dependent on local oxygen concentration.^65^ Oxygen accessibility may be higher in the aqueous environment compared to the particle core. The reactivity of hydrophilic, photocatalytic mCTA was compared to hydrophilic located photocatalytic nanoparticles towards the sulfide oxidation of 4-(methylthio)toluene, showing higher conversion for the mCTA due to an increased oxygen accessibility (Figure S17). When using a hydrophilic substrate, the product formation is favored within the hydrophilic microenvironment due to enhanced spatial proximity of the substrate to the active site and higher oxygen concentration compared to the hydrophobic system. When a more hydrophobic substrate is selected, it will preferentially partition into the hydrophobic phase of the self-assembled particle, increasing the spatial proximity to the hydrophobic active site. This increase in local concentration of the substrate around the hydrophobic active center might surpass the impact of slow oxygen diffusion into the hydrophobic portion of the nanoparticle and the inherently increased reaction rate by strongly electron-donating functional groups, resulting in a slightly higher conversion within the hydrophobic microenvironment. Ideally, the reactivity of 4,7-diphenyl-2,1,3-benzothiadiazole (the small molecule photocatalyst) should be compared to the polymeric photocatalyst under identical reaction conditions, but due to the photocatalyst’s insolubility in water, this is not possible.

**Figure 3:**
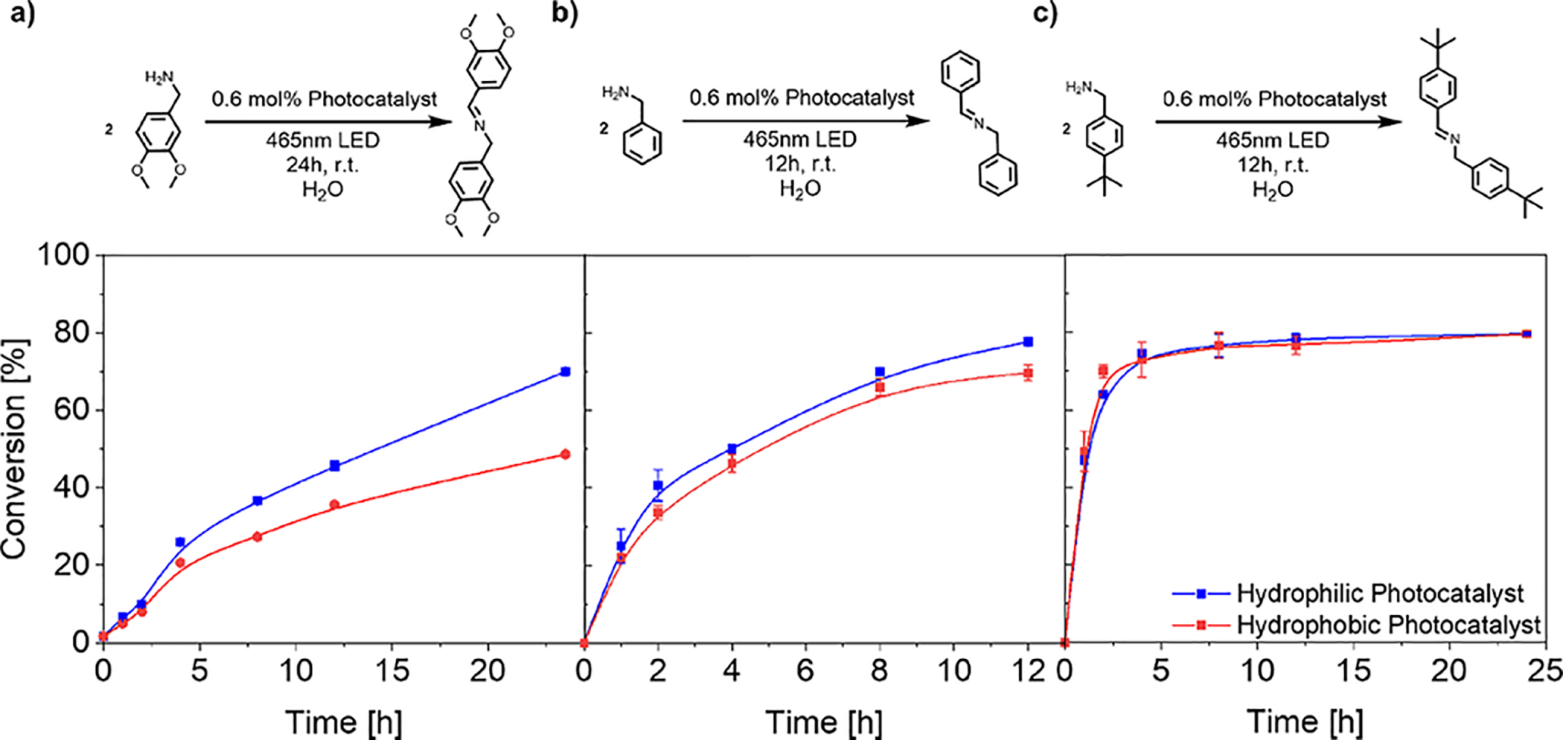
Kinetic profiles of the imine formation reaction performed by both polymeric, photocatalytic nanoparticles. Ranked from left to right from the most hydrophilic to the most hydrophobic substrates, underlining the dependence on substrate hydrophilicity. (a) Reaction of 3,4-dimethoxybenzylamine. (b) Reaction of benzylamine. (c) Reaction of 4-*tert*-butylbenzylamine. Conditions: Benzylic amine species (13.5 μmol), photocatalyst polymer (0.192 μmol, 0.6 mol % photocatalyst), dispersed in H_2_O (2 mL, sonicated 20 min), 15 °C, 7.14 W, 465 nm, 16 h. The conversion was calculated from GC measurements by comparison of peak area intensity.

To investigate the microenvironment impact on aromatic compounds, three substrates of varying log *P* values were investigated towards an oxidative imine formation (Figure 3). The imine formation of 3,4-dimethoxybenzylamine (log *P* = 0.83) was selected as the most hydrophilic substrate. Initially, a similar reaction rate was observed for both photocatalytic microenvironments. However, after 4 h, the reaction rate diverges, with the hydrophilic photocatalyst outperforming the hydrophobic one (product log *P* = 3.47) (Figure 3a). Due to increased substrate hydrophilicity compared to the other substrates, a higher substrate and oxygen accessibility for the oxidation of the amine is expected. Together with the decreased reactivity of the electron-withdrawing functional groups, a slower reaction rate is assumed, which is reflected in the slowly diverging kinetic profile.^66^ A similar but less prominent trend was also observed for the coupling of benzylamine (log *P* = 1.08), where the hydrophilic microenvironment slightly outperformed the hydrophobic one (product log *P* = 3.97) (Figure 3b). Due to its strong electron-donating character, which increases the oxidation reaction rate, the coupling of strongly lipophilic 4-*tert*-butylbenzylamine (log *P* = 2.78) showed no differences between the kinetic profiles (product log *P* = 7.38) (Figure 3c). It was expected that by investigation of hydrophobic functional groups such as *tert*-butyl groups, the hydrophobic-based photocatalyst would outperform its hydrophilic analog. The constant differences in reactivity suggest a limitation of the reaction rate within the hydrophobic microenvironment by hindered diffusional freedom. The self-assembled photocatalytic particles have a fully solvated hydrophilic shell and a precipitated hydrophobic core. Mass transfer into the core is therefore slower than into the corona, affecting reaction kinetics. This appears to be more prevalent for reactions that proceed through reactive oxygen species, where both diffusion of the substrate and oxygen are required. Also, the lifetime of these ROS is variable, depending on the microenvironment, which may also play an important role. With careful consideration of the reaction mechanism, the increased reaction rate of more hydrophobic substrates can be explained by stronger electron-donating character of the substituents, accelerating the formation of the corresponding reactive peroxo species. These factors might account for the marginal observed differences within the discussed imine formation (Figure 3). Since the reaction conditions require singlet oxygen and benzylic amines, which are consistently more lipophilic than the investigated sulfide oxidation substrates, the diffusional exchange between the continuous phase and the hydrophobic core could represent a rate-limiting transfer process.^66^ To further investigate the effect of the nanoparticle core, hydrophilic, photocatalytic mCTA was compared against hydrophilic located photocatalyst nanoparticles, showing higher conversion for the nanoparticle due to increased substrate accessibility by accumulation of benzylic amines within the core (Figure S18).

**Figure 4:**
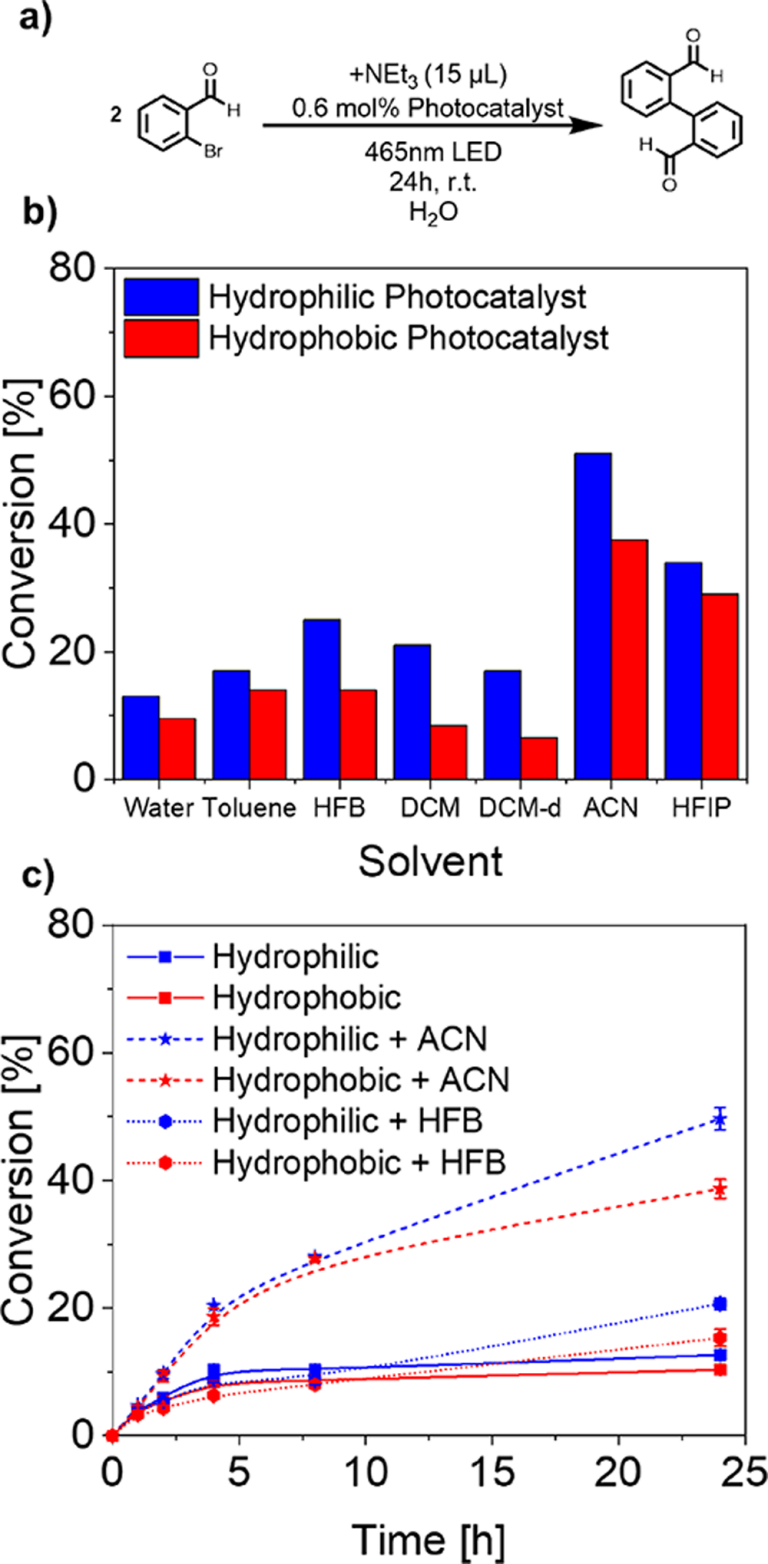
Investigation into the influences of different swelling additives on the chemical reactivity for a dimeric carbon–carbon coupling of 2-bromobenzaldehyde. (a) Reaction scheme. (b) Influences of different swelling solvents. Addition of 0.75 vol % solvent additive to increase conversion. (c) Kinetic profile of polymeric nanoparticles without the additive, with the best performing hydrophilic additive (ACN) and the best performing lipophilic additive (HFB), each performing the reductive dehalogenation. Conditions: 2-bromobenzaldeyhde (12.2 μmol), hydrophilic photocatalyst polymer (0.192 μmol, 0.6 mol % photocatalyst), dispersed in H_2_O (2 mL, sonicated 20 min), 15 °C, 7.14 W, 465 nm, 16 h. The conversion was calculated from GC measurements by comparison of peak area intensity.

Lastly, a reductive dehalogenation of 2-bromobenzaldehyde (log *P* = 2.61) with a subsequent radical–radical coupling (product log *P* = 3.2) was performed in an aqueous dispersion of the photocatalytic nanoparticles with additional 0.75 vol % of triethylamine as a sacrificial agent.^67,68^ Initially, a low total conversion was observed for both the hydrophilic and the hydrophobic photocatalyst, which may be due to the induced spatial division of 2-bromobenzaldehyde, accumulating inside the dense particle core due to its hydrophobic character, while the relatively hydrophilic triethylamine is located within the aqueous solution (Figure 4). To enhance core accessibility and improve phase exchange, 0.75 vol % of additional swelling solvents with varying hydrophilicity and singlet oxygen stabilization were investigated regarding their influence on reactivity (Figure 4b,c).

The addition of hydrophobic toluene showed a small improvement in conversion for both photocatalytic systems by swelling the particle core and increasing interphasic reagent exchange. Hexafluorobenzene (HFB) additionally stabilizes singlet oxygen, resulting in a 2-fold increase for the hydrophilic photocatalyst compared to the additive-free reaction (Figure 4b,c).^69,70^ The hydrophobic photocatalyst showed an identical conversion improvement after addition of HFB compared to the addition of toluene, possibly reasoned by diffusional limitations of the substrate and oxygen into the hydrophobic particle core. To combine water miscibility with singlet oxygen stabilization, the impact of hexafluoroisopropanol (HFIP) was investigated. HFIP gives increased total conversions compared to all hydrophobic additives and exhibiting a lower divergence between the hydrophilic and the hydrophobic photocatalyst. However, addition of water-miscible acetonitrile (ACN) resulted in the highest increase in reactivity for both catalytic systems yielding 5.4-fold and 3.6-fold increases for the hydrophilic and hydrophobic photocatalysts, respectively, when compared to additive-free conditions (Figure 4b,c). Within the presented reaction, solubility-limited reagent exchange between the particle core and the continuous phase seems to constitute the rate-limiting process when compared to the ROS lifetime. Depending on their polarity, partitioning of additives between the particle core and continuous phase occurs, allowing for increased substrate exchange and thereby acting as a phase-transfer agent. This is reflected by the strong conversion increase with water-miscible solvents, compared to hydrophobic solvents. Providing the opportunity of a biphasic reaction environment within a single photocatalytic system represents a powerful tool, regarding reaction control and substrate accessibility, yielding a reaction optimization of a maximal 5.4-fold increase in conversion.

## Conclusions

In conclusion, careful consideration of the microenvironment of photocatalytic active centers can be used to tune the reactivity of polymer photocatalysts. The incorporation of photocatalytic active centers into either hydrophilic or hydrophobic compartments of polymeric micelles results in significantly different reaction rates depending on the substrate. More hydrophilic substrates, which preferentially partition in the continuous phase, were converted significantly faster with the active center in the hydrophilic section. Change of the photocatalyst localization within the nanoparticle microenvironments showed drastically different performance with up to 62% conversion difference observed. More lipophilic substrates showed no to little conversion differences, possibly reasoned by limited mass transfer between the microenvironments combined with the inherent mechanism of the chosen reactions. Lastly, the reaction performance of these photocatalytic particles within a reductive dehalogenation was improved through addition of 0.75 vol % of a secondary swelling solvent, resulting in an up to 5.4-fold increase in reaction conversion. This swelling solvent enhances the accessibility of the core, while also increasing the lifetime of photocatalytically generated reactive oxygen species. We suggest careful consideration of the photocatalysts’ microenvironment as it may strongly influence the photocatalytic activity.

## Experimental Section

### Materials

4,7-Dibromobenzo[1,2,5]thiadiazole (>98%), (4-(4,4,5,5-tetramethyl-1,3,2-dioxaborolan-2-yl)phenyl)methanol (>98%), dimethylformamide (>99.5%), 1,4-dioxane (stab. with BHT, >99%), glycidyl methacrylate (stab. with MEHQ, >99.5%), tetrahydrothiophene (>99%), 2-(ethylthio)ethanol (>98%), 4,4,5,5-tetramethyl-2-phenyl-1,3,2-dioxaborolane (>98%), and 3,4-dimethoxybenzylamine (>97%) were purchased from Tokyo Chemical Industry (TCI). Methacryloyl chloride (stab. with MEHQ,>97%), dimethyl sulfoxide (>99.5%,anhydrous), 2-bromobenzaldehyde (98%), 4-((((2-carboxyethyl)thio)carbonothioyl)thio)-4-cyanopentanoic acid (95%), hexafluorobenzene (99%), hexafluoro-2-propanol (>99%), and 4-*tert*-butylbenzylamine (97%) were purchased from Sigma Aldrich. Tetrakis(triphenylphosphane)palladium (99%) and benzyl amine (99%) were purchased from ACROS Organics. Methyl-*p*-tolylsulfide (97%), benzyl methacrylate (stab. with 4-Methoxy phenol, 98%), and 4,4′-azobis(4-cyanopentanoic acid) (98%) were purchased from Alfa Aesar. Acetonitrile (>99.9%) was purchased from Honeywell. Toluene (>99.8%), ethanol (absolute), and dichloromethane (>99%) were purchased from Fisher Scientific. Na_2_CO_3_ (>99.5%) was purchased from Carl Roth. Deuterated dichloromethane (99.6%) was purchased from Deutero GmbH. Regenerated cellulose dialysis tubing (Sevapor 3, MWCO 3500, 16 mm diameter) was purchased from Serva.

### Methods

^1^H- and ^13^C-NMR spectra were recorded at room temperature on a Bruker AVIII 300 spectrometer, in deuterated solvents purchased from Sigma-Aldrich. Fourier-transform infrared spectroscopy measurements were conducted using a Bruker Vertex 70. Absorption spectra were recorded with an Agilent Cary 60 UV/Vis spectrometer with a xenon light source. Emission spectra were recorded with on a J&M Tidas FL3005SL fluorescence spectrometer with a Perkin Elmer diode array. GPC experiments were performed using a PSS SECcurity2 instrument using DMF with 1 g/L LiBr as the eluent and PMMA as the standard. Particle size distributions were analyzed by 90° dynamic light scattering measurements, which were performed using a Zetasizer Nano S500 and Nano S90. Transmission electron microscopy measurements were conducted using a JEM 1400 by drop-casting on carbonized copper grids. Gas chromatographic analysis was conducted on an Shimadzu GC-2010 plus GC- system equipped with a 7HG-G010-11 Phenomenex column and analyzed using a QP2010 ultra mass spectrometer.

### Synthesis of Photocatalyst BTPMA

#### 4-Bromo-7-phenylbenzo[1,2,5]thiadiazole

Into a 150 mL Schlenk tube with a stir bar, 25 mL of 2 M Na_2_CO_3_ (aq.) solution, 25 mL of toluene, and 9 mL of DMF were added. Subsequently, the solution was degassed with an argon stream for 15 min before adding 4,7-dibromobenzo[1,2,5]thiadiazole (1.5 equiv, 2.16 g, 7.35 mmol), 4,4,5,5-tetramethyl-2-phenyl-1,3,2-dioxaborolane (1 equiv, 1 g, 4.90 mmol), and tetrakis(triphenylphosphane)palladium (0.02 equiv, 113.25 mg, 98.00 μmol) in an argon counter stream. The reaction mixture was heated to 100 °C for 48 h with an attached reflux condenser under heavy stirring. After cooling down to room temperature, 30 mL of Milli-Q water was added followed by dichloromethane extraction (4× 25 mL), washing with brine, and drying over Na_2_SO_4_. After evaporation of all volatiles with the rotary evaporator, the crude mixture was purified using SiO_2_ column chromatography (gradient from 10% DCM:90% petrol ether to 70% DCM:30% petrol ether). A mixture of the product and biphenylbenzothiadiazole was obtained and used without further purification. (960 mg).

^1^H NMR (300 MHz, CDCl_3_, δ): 7.80 (m, 2H, Ar H), 7.60 (s, 2H; Ar H), 7.44 (m, 3H; Ar H).

#### (4-(7-Phenylbenzo[1,2,5]thiadiazol-4-yl)phenyl)methanol

Into a 150 mL Schlenk tube with a stir bar, 10 mL of a 2 M Na_2_CO_3_ (aq.) solution and 10 mL of DMF were added. Subsequently, the solution was degassed with an argon stream for 15 min before adding 4-bromo-7-phenylbenzo[1,2,5]thiadiazole (1.0 equiv, 550 mg, 1.89 mmol), (4-(4,4,5,5-tetramethyl-1,3,2-dioxaborolan-2-yl)phenyl)methanol (1.5 equiv, 663.31 mg, 2.83 mmol), and tetrakis(triphenylphosphane)palladium (0.02 equiv, 43.66 mg, 37.78 μmol) in an argon counter stream. The reaction mixture was heated to 90 °C for 24 h with an attached reflux condenser under heavy stirring. After cooling down to room temperature, 20 mL of Milli-Q water was added followed by dichloromethane extraction (3× 25 mL), washing with brine, and drying over Na_2_SO_4_. After evaporation of all volatiles with the rotary evaporator, the crude mixture was purified using SiO_2_ column chromatography (column deprotonated with 10%NEt_3_:90%EtOAc; gradient from 10% EtOAc:90% petrol ether to 70% EtOAc:30% petrol ether). The product was obtained as yellow crystals (253 mg, 42% yield).

^1^H NMR (300 MHz, CDCl_3_, δ): 8.01 (m, 4H, Ar H), 7.85 (s, 2H; Ar H), 7.59 (m, 4H; Ar H), 7.49 (m, 1H; Ar H) , 4.81 (d, 2H; CH_2_).

^13^C NMR (300 MHz, CDCl_3_, δ): 132.3 (2C; Ar C=N), 129.4 (Ar C), 129.3 (5C; Ar C), 128.1 (4C; Ar C), 126.9 (6C; Ar C), 64.7 (1C, C–OH).

#### 4-(7-Phenylbenzo[1,2,5]thiadiazol-4-yl)benzyl Methacrylate

Into an evacuated 50 mL Schlenk tube with a stir bar, 25 mL DCM (dry) was added. Subsequently, (4-(7-phenylbenzo[1,2,5]thiadiazol-4-yl)phenyl)methanol (1.0 equiv, 150 mg, 1.89 mmol) and triethylamine (15 equiv, 984.99 μL, 28.35 mmol) were added into an argon counter stream. The reaction mixture was stirred for 30 min before slowly adding methacryloyl chloride (1.2 equiv, 55 μL, 565.34 μmol) over 20 min. Afterwards, the mixture was kept under argon and stirring for 16 h. After that, 20 mL of Milli-Q water was added followed by dichloromethane extraction (3× 25 mL), washing with brine, and drying over Na_2_SO_4_. After evaporation of all volatiles with the rotary evaporator, the crude mixture was purified using SiO_2_ column chromatography (column deprotonated with 10 %NEt_3_:90% EtOAc, gradient from 10% EtOAc:90% petrol ether to 70% EtOAc:30% petrol ether). The product was obtained as bright yellow powder (134 mg, 73% yield).

^1^H NMR (300 MHz, CDCl_3_, δ): 7.98 (m, 4H, Ar H), 7.82 (s, 2H; Ar H), 7.61 (m, 4H; Ar H), 7.48 (m, 1H; Ar H), 4.81 (m, 2H; CH_2_), 6.23 (s, 1H; CH_2_), 5.64 (s, 1H; CH_2_), 5.32 (s, 2H; CH_2_), 2.03 (s, 3H; CH_3_).

^13^C NMR (300 MHz, CDCl_3_, δ): 144.7 (1C; C=O), 136.5 (2C; Ar C=N), 129.4 (2C; Ar C), 129.3 (3C; Ar C), 128.5 (3C; Ar C), 128.1 (6C; Ar C), 125.5 (2C; Ar C), 115.1 (1C; C4), 113.8 (1C; C2), 65.9 (1C, C2), 18.1 (1C; C1).

### Synthesis of Photocatalytic Nanoparticles

#### 2,3-Dihydroxypropyl Methacrylate

In a 50 mL round-bottom flask equipped with a stir bar, a 20 wt % solution of glycidyl methacrylate (5 g) in Milli-Q water (20 mL) was gassed with oxygen for 30 min. Afterwards, the biphasic solution was reacted for 16 h at 80 °C under heavy stirring. This crude aqueous solution was used without further purification for the following macro-CTA generation.

#### Hydrophilic Macrochain Transfer Agent (mCTA)

Into a 20 mL screw-cap vial equipped with a stir bar, freshly synthesized 2,3-dihydroxypropyl methacrylate solution (2.5 mL, 20 wt/vol%, aqueous) was transferred. After addition of DMSO (2.5 mL), 4,4′-azobis(4-cyanopentanoic acid) (0.2 equiv, 1.75 mg, 6.24 μmol) and 4-((((2-carboxyethyl)thio)carbonothioyl)thio)-4-cyanopentanoic acid (1 equiv, 9.6 mg, 31.22 μmol), the crude mixture was degassed with N_2_ for 20 min with light pressure. Afterwards, the solution was reacted under heavy stirring for 3 h at 70 °C. After cooling down to room temperature, the solution was dialyzed (3× EtOH:H_2_O, 1:1, exchanged after 6, 12, and 24 h followed by 3× water, 100%, exchanged every day), followed by lyophilization until dry. The product was obtained as a colorless, loose solid.

^1^H NMR (300 MHz, DMSO-*d*_6_, δ): 4.91 (br, 1H, OH), 4.67 (br, 1H; OH), 3.90 (br, 1H;), 3.68 (br, 2H; CH), 3.52 (br, 1H; CH_2_), 3.38 (br, 2H; CH_2_), 1.78 (br, 2H; CH_2_), 0.85 (m, 3H; CH_3_).

#### Copolymerized, Hydrophilic Located Photocatalyst Macrochain Transfer Agent (mCTA)

Into a 20 mL screw-cap vial equipped with a stir bar, freshly synthesized 2,3-dihydroxypropyl methacrylate solution (2.5 mL, 20 wt/vol %, aqueous) was transferred. After addition of DMSO (2.5 mL), 4,4′-azobis(4-cyanopentanoic acid) (0.2 equiv, 1.75 mg, 6.24 μmol), 4-(7-phenylbenzo[1,2,5]thiadiazol-4-yl)benzyl methacrylate (2 equiv, 24.13 mg, 62.43 μmol), and 4-((((2-carboxyethyl)thio)carbonothioyl)thio)-4-cyanopentanoic acid (1 equiv, 9.6 mg, 31.22 μmol), the crude mixture was degassed with N_2_ for 20 min with light pressure. Afterwards, the solution was reacted under heavy stirring for 5 h at 70 °C. After cooling down to room temperature, the solution was dialyzed (3× EtOH:H_2_O, 1:1, exchanged after 6, 12, and 24 h followed by 3× water, 100%, exchanged every day) followed by lyophilization until dry. The product was obtained as a slightly yellow, loose solid.

^1^H NMR (300 MHz, DMSO-*d*_6_, δ): 8.00 (m, 5H; Ar H), 7.41 (m, 6H; Ar H), 4.91 (br, 1H, OH), 4.67 (br, 1H; OH), 3.90 (br, 1H;), 3.68 (br, 2H; CH), 3.52 (br, 1H; CH_2_), 3.38 (br, 2H; CH_2_), 1.78 (br, 2H; CH_2_), 0.85 (m, 3H; CH_3_).

#### Copolymerization of the Photocatalyst and Hydrophobic Block with Hydrophilic mCTA

Into a 20 mL screw-cap vial equipped with a stir bar, hydrophilic mCTA P(GMA)100 (1 equiv, 300 mg, 18.38 μmol) was transferred. After addition of H_2_O (3 mL), DMSO (3 mL), 4,4′-azobis(4-cyanopentanoic acid) (0.2 equiv, 1.03 mg, 3.68 μmol), 4-(7-phenylbenzo[1,2,5]thiadiazol-4-yl)benzyl methacrylate (2 equiv, 14.20 mg, 36.76 μmol), and benzyl methacrylate (200 equiv, 647.68 mg, 3.68 mmol), the crude mixture was degassed with N_2_ for 20 min with light pressure. Afterwards, the solution was reacted under heavy stirring for 24 h at 70 °C, forming an opaque dispersion. After cooling down to room temperature, the solution was dialyzed (3× EtOH:H_2_O, 1:1, exchanged after 6, 12, and 24 h followed by 3× water, 100%, exchanged every day) followed by lyophilization until dry. The product was obtained as a slightly yellow, loose solid.

^1^H NMR (300 MHz, DMSO-*d*_6_, δ): 7.52 (m, 11H; Ar H), 7.25 (br, 5H; Ar H), 4.86 (br, 1H, OH), 4.67 (br, 1H; OH), 3.93 (br, 1H;), 3.68 (br, 2H; CH), 3.52 (br, 1H; CH_2_), 3.38 (br, 2H; CH_2_), 1.78 (br, 2H; CH_2_), 0.80 (m, 3H; CH_3_).

#### Copolymerization of the Hydrophobic Block with Hydrophilic, Photocatalytic mCTA

Into a 20 mL screw-cap vial equipped with a stir bar, hydrophilic mCTA P(GMA)100 (MaBTPh)1 (1 equiv, 300 mg, 17.95 μmol) was transferred. After addition of H_2_O (3 mL), DMSO (3 mL), 4,4′-azobis(4-cyanopentanoic acid) (0.2 equiv, 1.01 mg, 3.59 μmol), and benzyl methacrylate (200 equiv, 632.70 mg, 3.59 mmol), the crude mixture was degassed with N_2_ for 20 min with light pressure. Afterwards, the solution was reacted under heavy stirring for 24 h at 70 °C, forming an opaque dispersion. After cooling down to room temperature, the solution was dialyzed (3× EtOH:H2O, 1:1, exchanged after 6, 12, and 24 h followed by 3× water, 100%, exchanged every day) followed by lyophilization until dry. The product was obtained as a slightly yellow, loose solid.

^1^H NMR (300 MHz, DMSO-*d*_6_, δ): 7.52 (m, 11H; Ar H), 7.25 (br, 5H; Ar H), 4.86 (br, 1H, OH), 4.67 (br, 1H; OH), 3.93 (br, 1H;), 3.68 (br, 2H; CH), 3.52 (br, 1H; CH_2_), 3.38 (br, 2H; CH_2_), 1.78 (br, 2H; CH_2_), 0.80 (m, 3H; CH_3_).

### Photoreactor Setup

All photochemical reactions were performed within the customized photoreactor depicted in Figure S1 bearing reaction vial slots, each provided with 6 blue light LEDs (Tru Components HighPower, 1.4 W per LED, λ = 460–470 nm, see Figure S2). LEDs and reaction vials were constantly kept at 15 °C using the built-in water cooling system. Additionally, constant stirring at 450 rpm with PTFE-coated stir bars was ensured.

### General Photocatalytic Procedure

Photocatalytic polymer stock solution was prepared freshly before every reaction. Therefore, the polymer was dissolved in Milli-Q water (10 mg polymer/300 μL water, 75.3 μg photocatalyst) and sonicated for 20 min. After cooling down, the solution was used straight away.

### Photocatalytic Sulfide Oxidation

For each reaction, polymer stock solution (300 μL, 10 mg polymer, 75.3 μg photocatalyst) was added into a 4 mL screw-cap vial equipped with a stir bar followed by addition of water (Milli-Q, 1.7 mL) and the sulfide substrate to give a 10 mM solution (0.3 mol % photocatalyst). Under constant stirring at 450 rpm and cooling to 15 °C, the vials were placed into a photoreactor slot and irradiated with blue light for 24 h. Afterwards, each sample was analyzed according to the GC/MS procedure.

### Photocatalytic Imine Formation

For each reaction, polymer stock solution (300 μL, 10 mg polymer, 75.3 μg photocatalyst) was added into a 4 mL screw-cap vial equipped with a stir bar followed by addition of water (Milli-Q, 1.7 mL) and the amine substrate to give a 5 mM solution (0.6 mol % photocatalyst). Under constant stirring at 450 rpm, and cooling to 15 °C, the vials were placed into a photoreactor slot and irradiated with blue light for 24 h. Afterwards, each sample was analyzed according to the GC/MS procedure.

### Photocatalytic C–C Bond Coupling

For each reaction, polymer stock solution (300 μL, 10 mg polymer, 75.3 μg photocatalyst) was added into a 4 mL screw-cap vial equipped with a stir bar followed by addition of water (Milli-Q, 1.7 mL), triethylamine (10 equiv, 15 μL), solvent additive (15 μL), and the halogen substrate to give a 5 mM solution (0.6 mol % photocatalyst). Under constant stirring at 450 rpm and cooling to 15 °C, the vials were placed into a photoreactor slot and irradiated with blue light for 24 h. Afterwards, each sample was analyzed according to the GC/MS procedure.

## Supporting Information Available

Enhanced general information, experimental procedures, characterization and analytical data (PDF)
