# Supplementary material for: Tunable Photocatalytic Selectivity by Altering the Active Center Microenvironment of an Organic Polymer Photocatalyst
Source: ACS Appl Mater Interfaces. 2023 Jan 3;15(2):2891–900. doi: 10.1021/acsami.2c17607 (PMC9869337; doi:10.1021/acsami.2c17607)
Supplement: Supplementary file 1 — am2c17607_si_001.pdf [file am2c17607_si_001.pdf]

Supporting Information

Tunable Photocatalytic Selectivity by Altering the Active Center Microenvironment of an Organic Polymer Photocatalyst

Julian Heuer,[a] Thomas Kuckhoff,[a] Rong Li,[a] Katharina Landfester [a] and Calum T. J. Ferguson [a,b]*

[a] Max Planck Institute for Polymer Research, Ackermannweg 10, 55128 Mainz, Germany

E-mail: ferguson@mpip-mainz.mpg.de

[b] School of Chemistry, University of Birmingham, Edgbaston, Birmingham, B15 2TT

E-mail: c.ferguson.1@bham.ac.uk

Table of Contents

1. General Experimental Information 3

2. General Analytical Techniques 3

3. Photophysical Analytical Techniques 3

4. Macromolecular Analytical Techniques 3

5. Purification Techniques 4

6. Solvents 4

7. Photoreactor setup 4

8. GC-MS Analysis 5

9. General Synthetic Procedures 6

9.1 Photocatalytic Sulfide Oxidation 6

9.2 Photocatalytic Imine Formation 6

9.3 Photocatalytic C-C Bond Coupling 6

10. NMR Analysis of the Polymeric Nanoparticles 7

11. Macromolecular Characterization 9

12. FTIR Analysis 10

13. Photophysical Characterization 12

14. Log P-Value Comparison 14

15. Recyclability Test 15

16. Comparison of Photocatalytic mCTA against Photocatalytic Nanoparticles 15

17. TEM Analysis 17

18. Experimental Section/Methods 19

19. Investigation of the Photocatalytic Behaviour According to Reaction Condition Variation by Means of Sulfide Oxidation Reaction.[a] 26

20. Investigation of the Photocatalytic Behaviour According to Reaction Condition Variation by Means of Imine Formation Reaction.[a] 27

21. Investigation of the Photocatalytic Behaviour According to Reaction Condition Variation by Means of Aromatic, Radical C-C Coupling Reaction.[a] 28

22. Appendix 29

1. General Experimental Information

All organic synthesis for the photocatalysts were performed in oven-dried glassware under argon, unless otherwise stated. Polymerization reactions were conducted in 20 mL screw-top vials with a PTFE-membrane cap. Reactions investigating the catalytic activity of the substrates were performed in 4 mL screw-top vials. Reaction temperatures are referred to the ones of the heating/cooling media (aluminium heating block, water cooling), unless otherwise stated. Reagents were purchased from Sigma-Aldrich, Merck, ACROS Organics, Alfa Aesar, TCI and used without further purification. Reactions were conducted, using a PTFE-coated, egg-shaped stir bar at around 1900 rpm. Solvents were removed by rotary evaporation under reduced pressure, heating the solution with a water bath at 40-60 °C. Residual high boiling solvents were removed in vacuo (<1 mbar) at room temperature. Formed compounds were characterized by ^{1}H-NMR, ^{13}C- NMR, APCI-MS and GC-MS. Throughout all experiments Millipore quality water (Milli-Q-Synthesis 230 V/50 Hz, Milli-Q Q-Gard®2, 18.2 MΩ cm) was used, unless stated otherwise.

2. General Analytical Techniques

^{1}H- and ^{13}C-NMR spectra were recorded at room temperature on a Bruker AVIII 300 spectrometer (^{1}H: 400.13 MHz; ^{13}C: S 4 100.62 MHz), in deuterated solvents (> 99.5 Deuteration) purchased from Sigma-Aldrich, stored at 4 °C ($CDCl_3$, CD_2Cl_2, DMSO-d6, D_2O). Chemical shifts (δ) for ^{1}H and ^{13}C NMR spectra were referenced against TMS (tetramethylsilane) and are given in parts per million (ppm). First order multiplicities in ^{1}H NMR signals were reported using the following abbreviations: s = singlet, d = doublet, t = triplet, q = quartet, p = quintet, h =sextet; m = multiplet, br = broad signal. Data processing of NMR spectra was done with MestReNova 14.2.3.

APCI-MS were recorded on an Advion ASAP (Advion Inc., Ithaca) with DART injection. Considering $[M+H]^+$, $[M+NH_4]^+$, $[M+Na]^+$ or $[M+K]^+$ for positive Ionization methods.

Fourier-transform infrared spectroscopy measurements were conducted using a Bruker Vertex 70 (Bruker Inc. , Billerica), using a well dispersed, homogenous mixture of 2 mg sample together with 200 mg of KBr (dried and spectroscopic grade) to from stable KBr discs with a Perkin Elmer hydraulic press (15 tons pressure, 4 min), aided by a high vacuum pump. Data processing of obtained FTIR spectra was done with Bruker OPUS 7.8 software.

3. Photophysical Analytical Techniques

Absorption spectra were recorded with an Agilent Cary 60 UV/Vis spectrometer with xenon light source, using response time 0.04 s, 1.5 nm spectral bandwidth in 0.5 nm intervals and baseline correction. All measurements were conducted in 10x10 mm VWR ES-quartz cuvettes (dimensions: 12.5×12.5×45 mm) fitted with PTFE caps.

Emission spectra were recorded with on a J&M Tidas FL3005SL fluorescence spectrometer with a Perkin Elmer diode array (300-1100 nm). Data analysis was conducted by fitting with a Gaussian function and normalization using Origin 2019b (V. 9.65).

EPR (Electron Paramagnetic Resonance) was measured on a Magnettech Miniscope MS200 spectrometer at room temperature, microwave frequency: 9.391 GHz, microwave power: 10 mW, modulation amplitude: 9.8 G, field modulation: 0.2 mT at 100 kHz, scan time: 60 s.

4. Macromolecular Analytical Techniques

GPC experiments were performed using an PSS SECcurity^2 instrument consisting of a pump, auto sampler and column oven .A column set consisting of 3 columns: GRAM 1000 Å , GRAM 1000Å and GRAM 100Å (PSS Standards Service GmbH, Mainz, Germany), all of 300 x 8 mm

and 10 μm average particle size were used at a flow rate of 1.0 mL/min and a column temperature of 60 °C. As eluent DMF with 1 g/L LiBr was used. The samples having 1 mg/ml concentration were filtered prior to measurement through 0.45 μm PTFE filter. The injection volume was 100 μL. Detection was accomplished with a RI detector and UV detector at 270 nm wavelength.

Data acquisition and evaluation was performed using PSS WINGPC UniChrom (PSS Polymer Standards Service GmbH, Mainz, Germany). Calibration was carried out by using poly(methyl methacrylate) provided by PSS Polymer Standards Service GmbH (Mainz, Germany).

Particle size distributions were analysed by 90° dynamic light scattering (DLS) measurements, which were performed using a Zetasizer Nano S500 and Nano S90(Malvern® Pananalytical Mdt., Worcestershire). Therefore, aqueous particle dispersions were measured in 10x10 mm disposable, semi-micro polystyrene cuvettes (1.5-3 mL volume). Data acquisition was performed by Zetasizer Software (Version: 7.2, Malvern®).

Transmission electron microscopy measurements were conducted using a JEM 1400 (JEOL ®, Tokyo), equipped with a 120 kV tungsten emitter, Fishione® high angle annular darkfield detector combined with an on axis Gatan® US1000 2k CCD camera. Data acquisition was conducted using Gatan® Microscopy Suite (Version:2.11.1404, DigitalMicrograph, Gatan®). Sample preparation was performed by drop-casting on carbonized copper grids (300 mesh, C-layer 10-20 nm), followed by staining with uranyl acetate (30 s, 4% aqueous solution) and solvent deduction with filter paper.

5. Purification Techniques

Flash column chromatography was performed on Machery-Nagel 60 M silica gel (40-63 microns) under pressure gradients using the Biotage® Isolera One Purification system, within Biotage® KP-Sil cartridges of differing size. Product elution was analysed by the internal UV-detector (200 -800 nm). Thin layer chromatography was conducted using Machery-Nagel Alugram® SIL G/UV_{254} pre-coated aluminium sheets and was visualized by UV light (254 nm or 366 nm), Iodine chamber or stained with basic potassium permanganate solution (1.5 g $KMnO_4$, 10 g K_2CO_3, 1.25 mL 10% NaOH in 200 mL of distilled water). All dialysis purifications were performed using Spectra/Por® 1 to 4 Spectra/Por® 3 - MWCO 3500, 18 mm, regenerated cellulose (Carl Roth, Germany), hydrating membranes for 15 min in Milli-Q water (200 mL), closing the membranes with knots.

6. Solvents

Dry solvents DMSO, DMF and toluene were bought from ACROS Organics, containing H_2O <50 ppm, stored under activated molecular sieves of 3 Å pore diameter and transferred within an argon counter stream. P.a. grade triethylamine was purchased from Sigma-Aldrich, with a purity of ≥99.5%, stored under identical conditions to dry solvents. Solvents for flash column chromatography (EtOAc, Chloroform, n-Hexane, CH_2Cl_2 and MeOH) were purchased in technical grade and used without further purification.

7. Photoreactor Setup

All photochemical reactions were performed within the customized photoreactor depicted in Figure S 1. Bearing reaction vial slots, each provided with 6 blue light LEDs (Tru Components™ HighPower, 1.4 W per LED, λ=460-470 nm, see Figure S 2). LEDs and reaction vials were constantly kept at 15 °C using the built-in water cooling system. Additionally, constant stirring at 450 rpm with PTFE-coated stir bars was ensured.

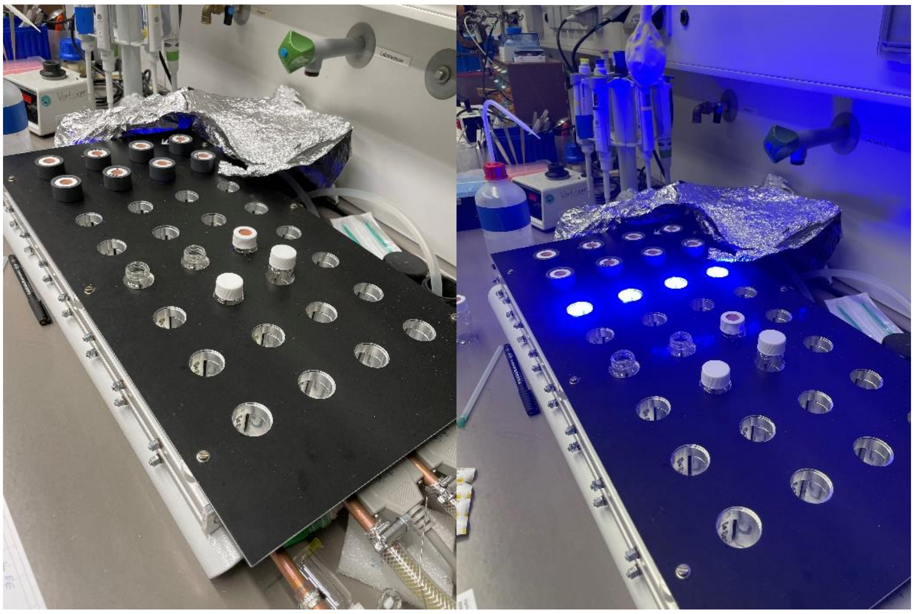

Figure S 1: Customized photoreactor setup, showing all vial slots turned off **(left)** and 4 vial slots turned on **(right)**.

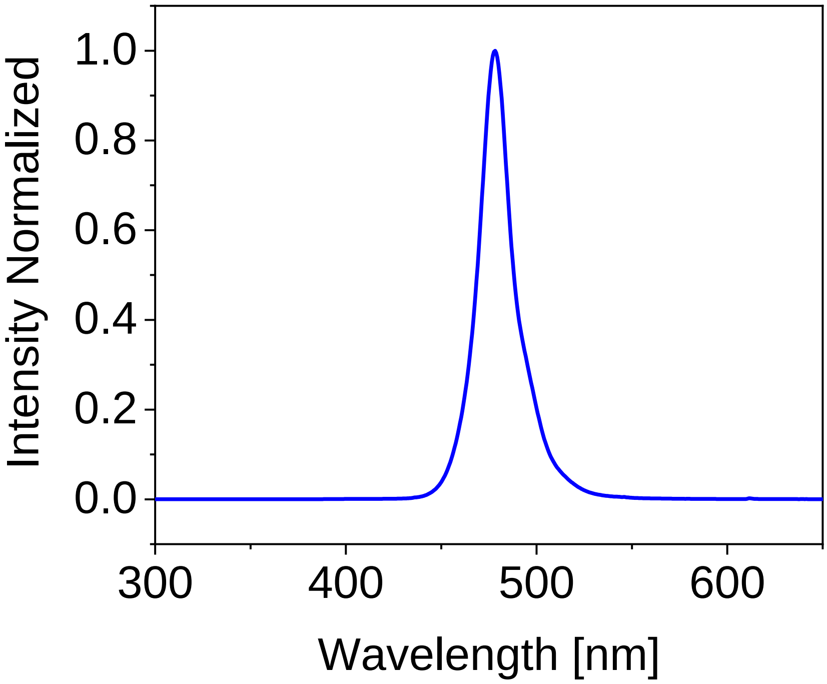

Figure S 2: Measured emission spectra of the used Tru Components™ HighPower-LEDs

8. GC-MS Analysis

Gas chromatographic analysis was conducted via on an Shimadzu GC-2010 plus GC- system equipped with a 7HG-G010-11 Phenomenex column (0.25 mm x 30 m (diameter:length), film thickness: 0.25 µm) and analysed using QP2010 ultra mass spectrometer. For peak separation two methods were used: 1) for small molecules with difficult separation (tetrahydrothiophene , 2-(ethylsulfanyl)ethanol and methyl p-tolyl sulfide) and 2) for larger molecules with longer retention time (3,4-dimethoxybenzylamine, benzylamine and 4-tert-butylbenzylamine).

1) Splitless injection, injection temperature 250 °C, column oven 50 °C. Temperature gradient: 50 °C hold 2 min to 160 °C with a rate of 15 °C/min hold for 2 min to 280° C with a rate of 20 °C/min hold for 8 min. Total flow at 23.1 mL/min, 42.0 kPa Pressure, 0.86 mL/min column flow.
2) Splitless injection, injection temperature 280 °C, column oven 100 °C. Temperature gradient: 100 °C to 280 °C with a rate of 10 °C/min hold for 5 min Total flow at 19.6 mL/min, 43.6 kPa Pressure, 0.70 mL/min column flow.
Preparation of GC/MS samples were performed by addition of 200 µL of crude solution mixture into 2 mL acetonitrile, followed by drying with Mg_2SO_4 and then transferred into 2 mL GC vials, rewashing Mg_2SO_4 with 1 mL acetonitrile. GC-MS spectra were analysed with GCMSolution Postrun Analysis (Version: 4.45 SP1).

9. General Synthetic Procedures

Photocatalytic polymer stock solution was prepared freshly before every reaction. Therefore, the polymer was dissolved in Milli-Q water (10 mg polymer/300 µL water, 75.3 µg photocatalyst) and sonicated for 20 min. After cooling down, the solution was used straight away.

9.1 Photocatalytic Sulfide Oxidation

For each reaction, polymer stock solution (300 µL, 10 mg polymer, 75.3 µg photocatalyst) was given into a 4 mL screw-cap vial, equipped with a stir bar. Followed by addition of water (Milli-Q, 1.7 mL) and the sulfide substrate to give a 10 mM solution (0.3 mol% photocatalyst). Under constant stirring at 450 rpm, and cooling to 15 °C, the vials were placed into a photoreactor slot and irradiated with blue light for 24 h. Afterwards, each sample was analysed according to the GC/MS procedure.

9.2 Photocatalytic Imine Formation

For each reaction, polymer stock solution (300 µL, 10 mg polymer, 75.3 µg photocatalyst) was given into a 4 mL screw-cap vial, equipped with a stir bar. Followed by addition of water (Milli-Q, 1.7 mL) and the amine substrate to give a 5 mM solution(0.6 mol% photocatalyst). Under constant stirring at 450 rpm, and cooling to 15 °C, the vials were placed into a photoreactor slot and irradiated with blue light for 24 h. Afterwards, each sample was analysed according to the GC/MS procedure.

9.3 Photocatalytic C-C Bond Coupling

For each reaction, polymer stock solution (300 µL, 10 mg polymer, 75.3 µg photocatalyst) was given into a 4 mL screw-cap vial, equipped with a stir bar. Followed by addition of water (Milli-Q, 1.7 mL), triethylamine (10 Eq., 15 µL), solvent additive (15µL) and the halogen substrate to give a 5 mM solution(0.6 mol% photocatalyst). Under constant stirring at 450 rpm, and cooling to 15 °C, the vials were placed into a photoreactor slot and irradiated with blue light for 24 h. Afterwards, each sample was analysed according to the GC/MS procedure.

10. NMR Analysis of the Polymeric Nanoparticles

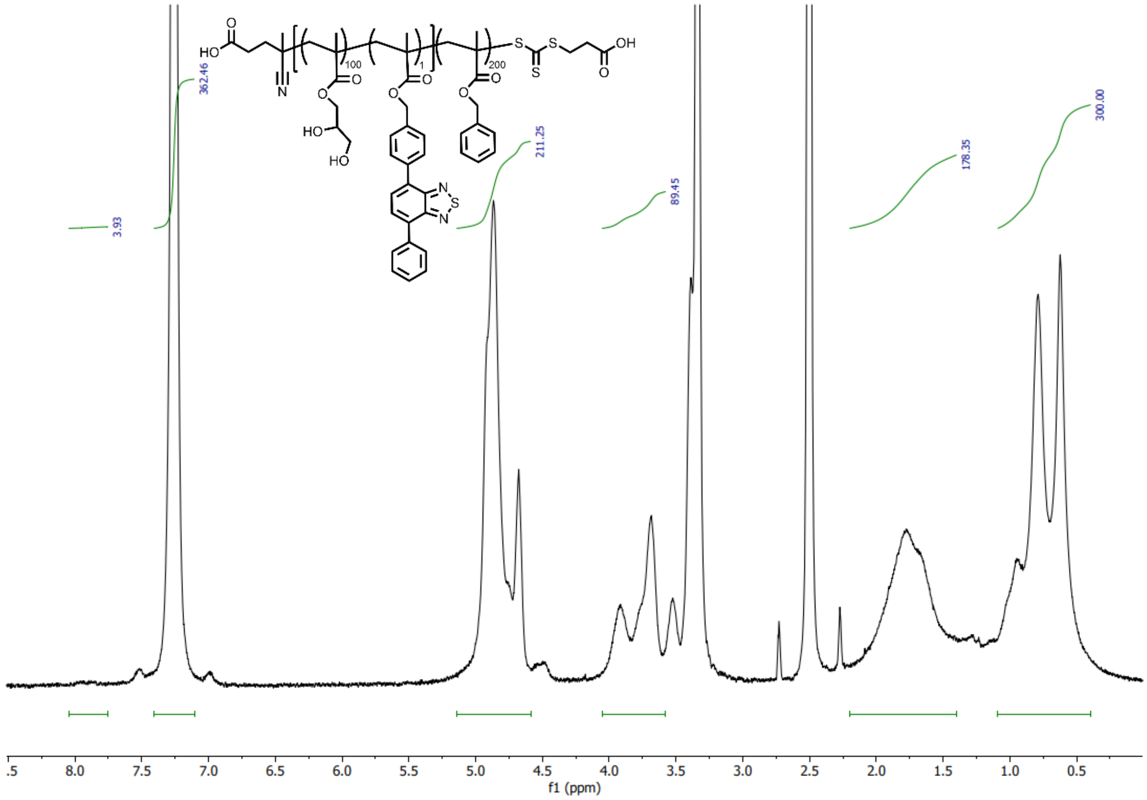

Figure S 3: ^{1}H-NMR spectrum of the amphiphilic block-copolymer with hydrophilic photocatalyst $P(GMA)_{100}(BTP)_{1}P(BzMA)_{200}$.

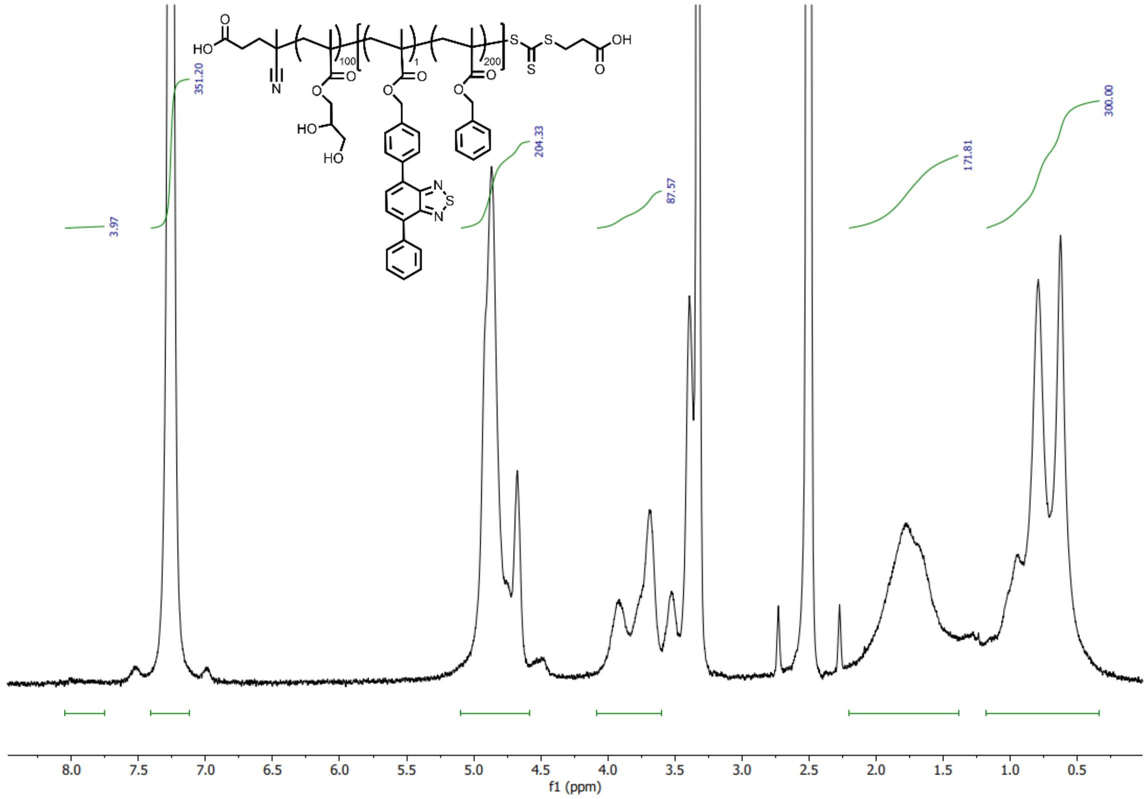

Figure S 4: ^{1}H-NMR spectrum of the amphiphilic block-copolymer with hydrophobic photocatalyst $P(GMA)_{100}(BTP)_{1}P(BzMA)_{200}$.

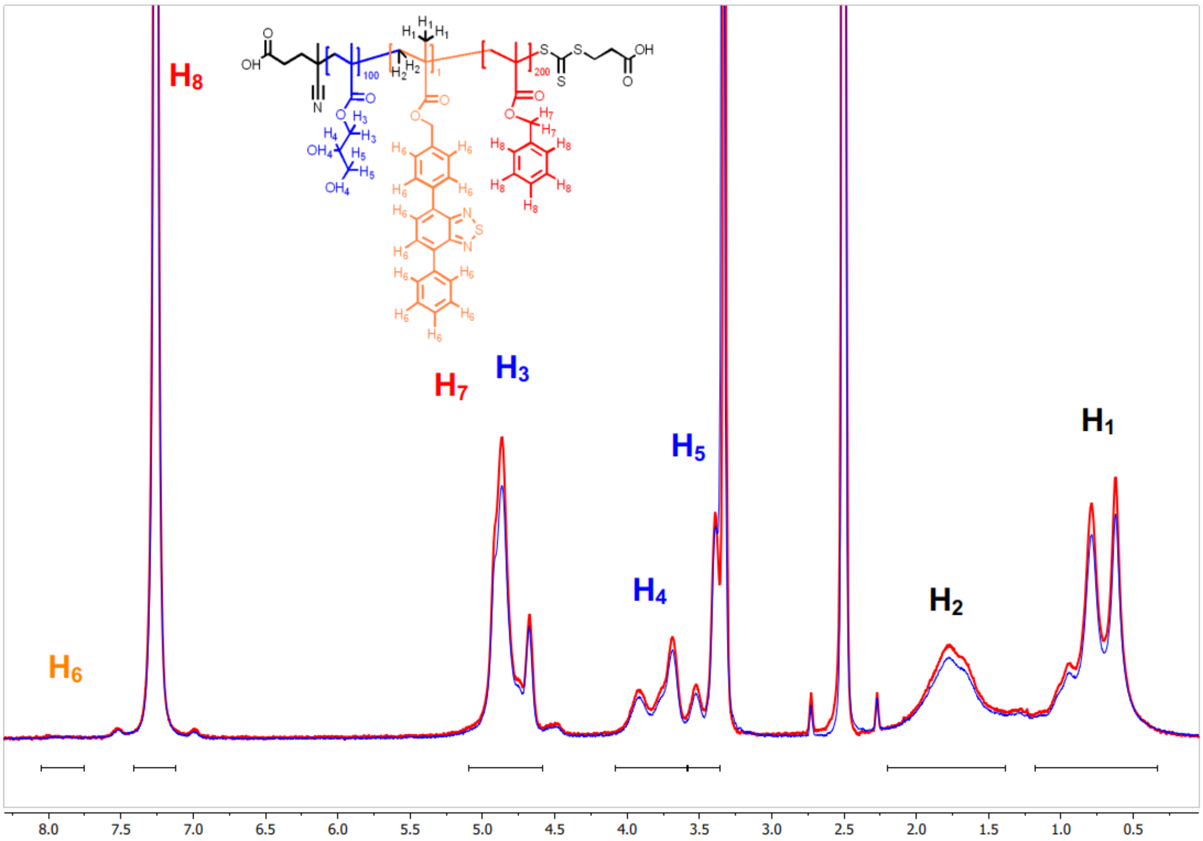

Figure S 5: Superimposed 1H-NMR spectra of the amphiphilic block-copolymer with hydrophobic photocatalyst (red) and hydrophilic photocatalyst (blue).

11. Macromolecular Characterization

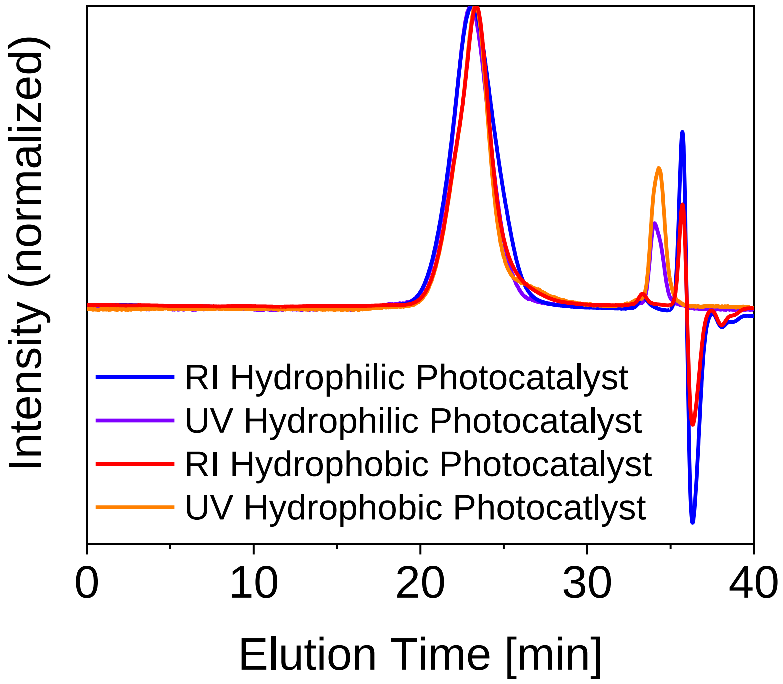

Figure S 6: Comparison of the GPC elution diagram of both photocatalytic polymers. Using DMF as eluent with PMMA standard.

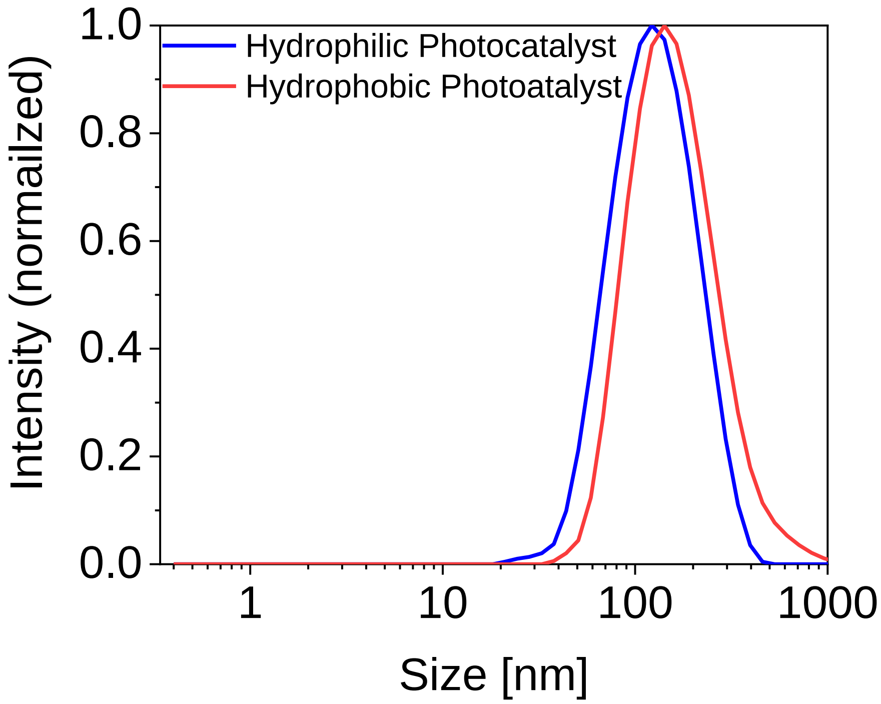

Figure S 7: Dynamic light scattering measurements of both photocatalytic nanoparticles. Comparison of both systems shows minimal differences in size. Particle distributions (PD) is 1.5 for hydrophilic and hydrophobic localised photocatalyst.

12. FTIR Analysis

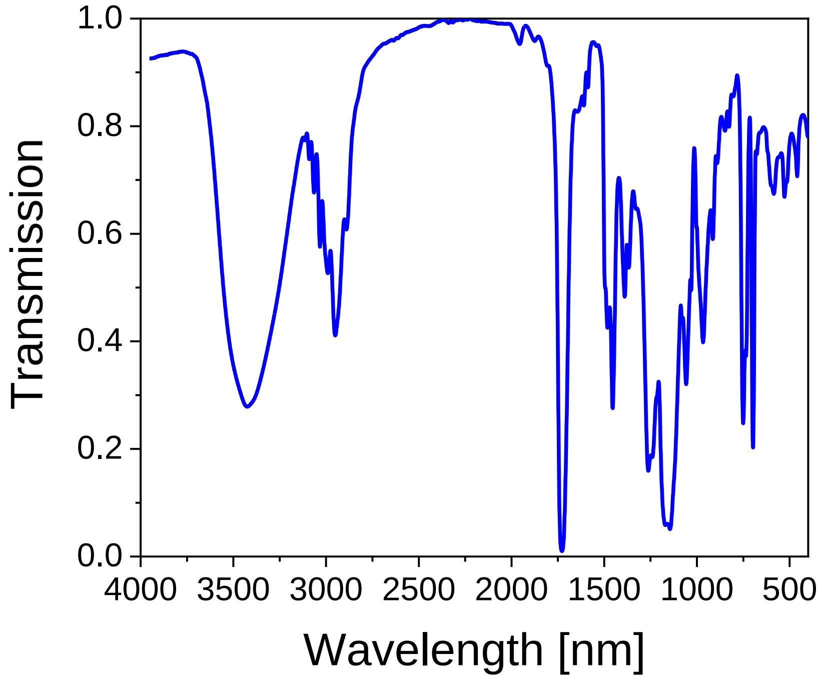

Figure S 8: FTIR-spectrum of the hydrophilic located photocatalyst polymer.

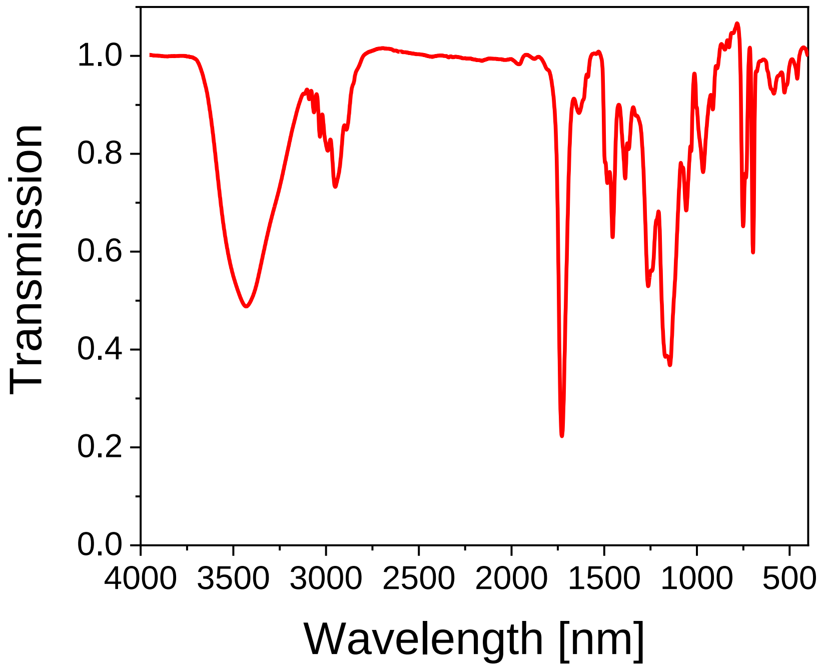

Figure S 9: FTIR-spectrum of the hydrophobic located photocatalyst polymer

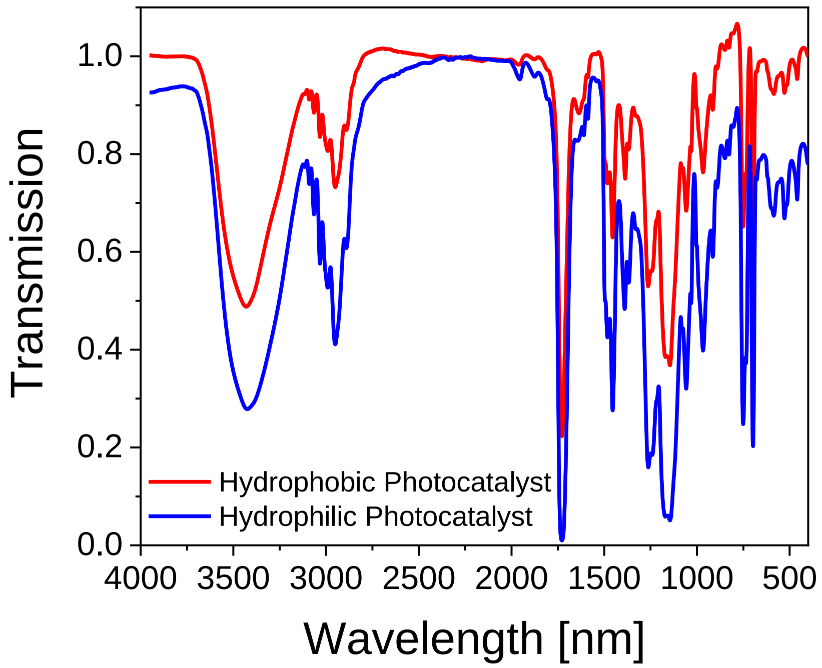

Figure S 10: Comparison of the hydrophilic and hydrophobic located photocatalyst polymer.

13. Photophysical Characterization

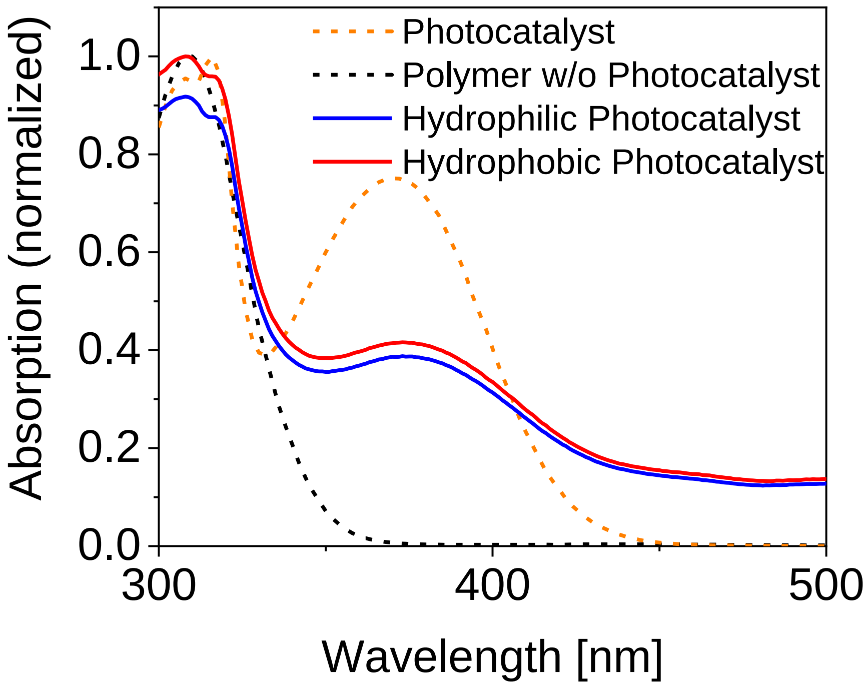

Figure S 11: UV/VIS-absorption spectra, comparing the photophysical activity of both nanoparticle systems. Showing minimal differences of 3 % between the systems at 373 nm.

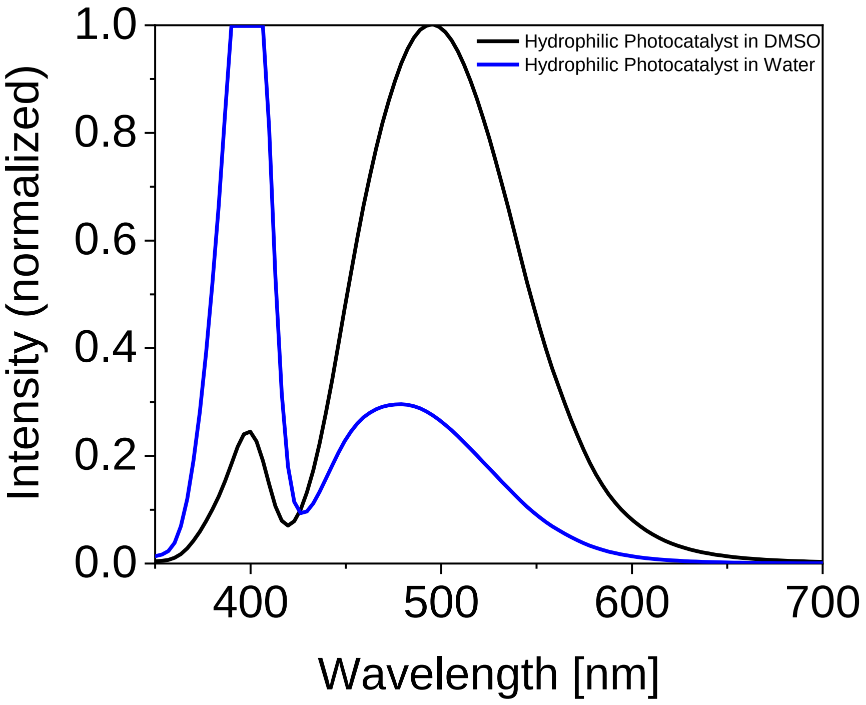

Figure S 12: UV/VIS-emission spectra for the hydrophilic localised photocatalyst nanoparticle. Comparison between water and DMSO as solvent reveal high scattering peak at 400 nm in water and higher photocatalyst emission in DMSO at 501 nm.

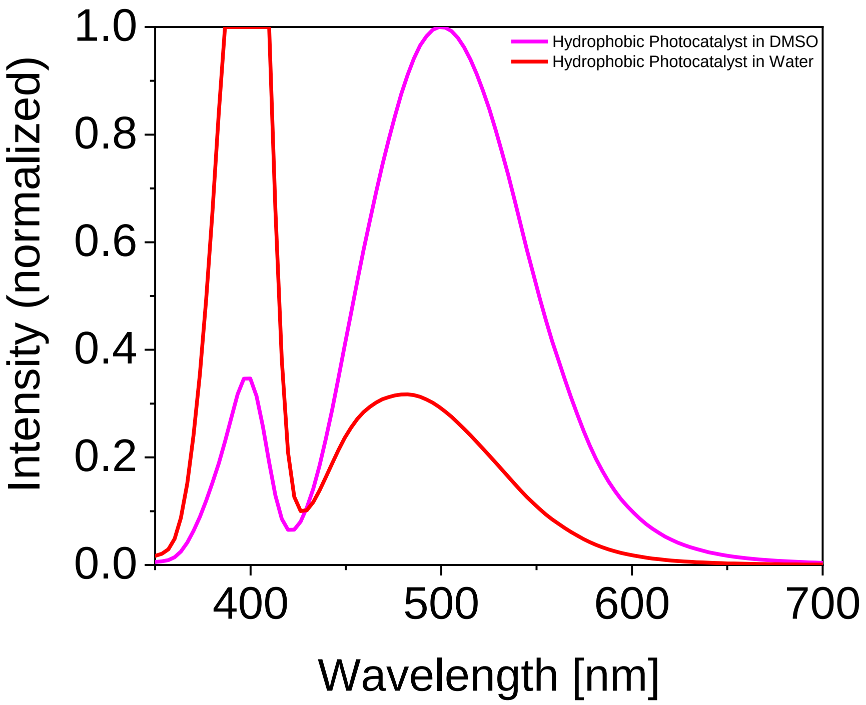

Figure S 13: UV/VIS-emission spectra for the hydrophobic localised photocatalyst nanoparticle. Comparison between water and DMSO as solvent reveal high scattering peak at 400 nm in water and higher photocatalyst emission in DMSO at 501 nm.

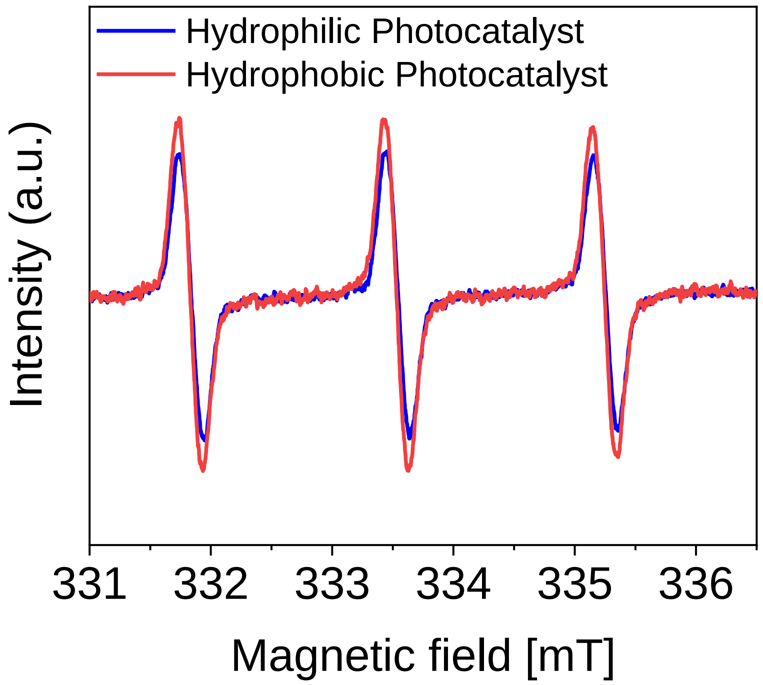

Figure S 14: EPR spectra, comparing the singlet oxygen generation of both nanoparticle systems.

14. Log P-Value Comparison

Table S 1: Calculated Log P values for investigated compounds and corresponding products.

Starting Material	Log P	Product	Log P
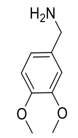	0,83	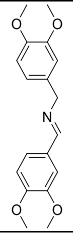	3,47
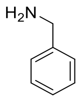	1,08	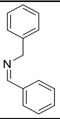	3,97
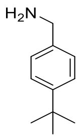	2,78	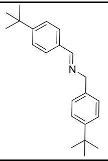	7,38

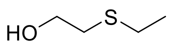	0,63	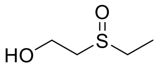	-1,32
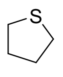	1,13	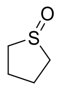	-0,83
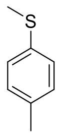	2,96	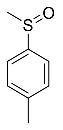	1,18
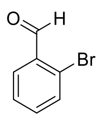	2,61	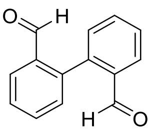	3,2

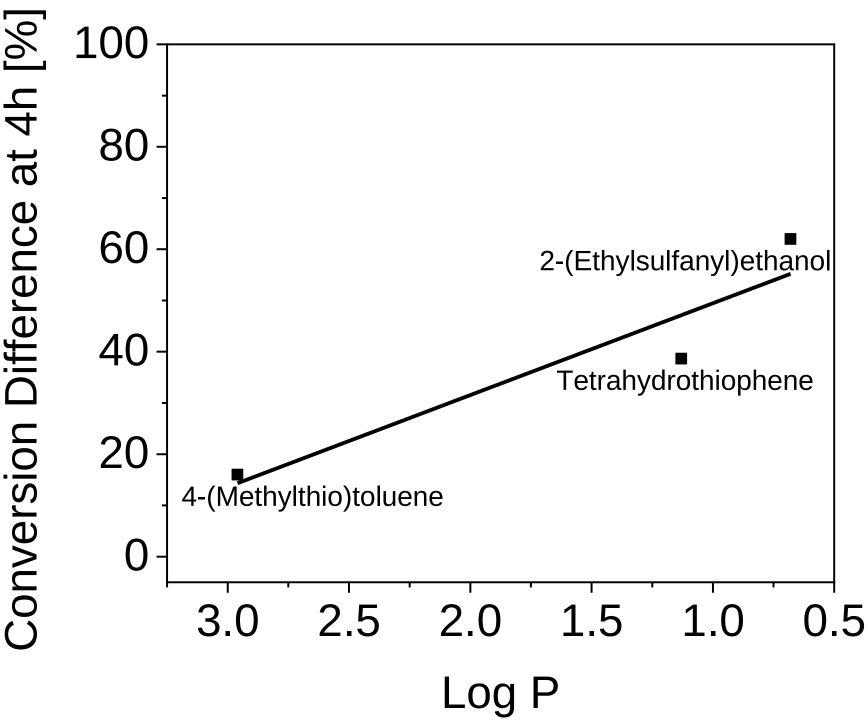

Figure S 15: Log P-values of sulfide oxidation starting materials against conversion rate after 4 h reaction time. (Log P Values obtained by ChemDraw 20.1, using the methodolgy of [1])

15. Recyclability Test

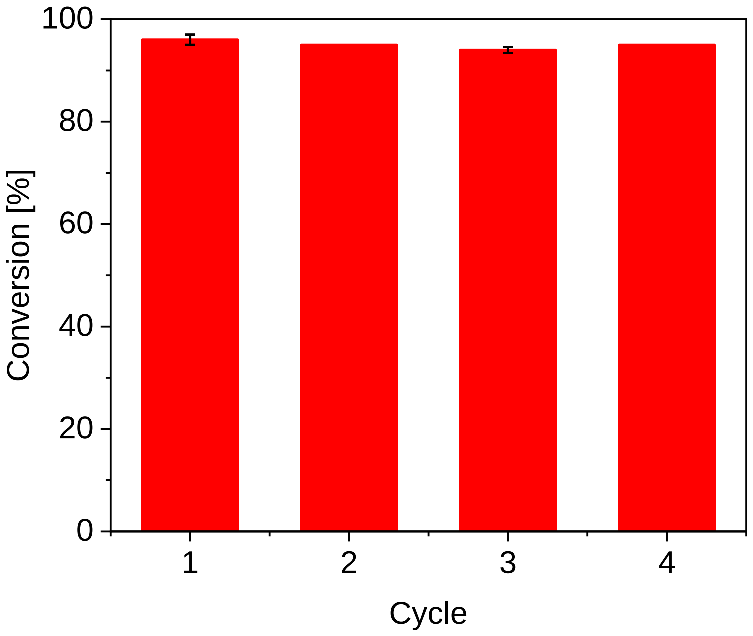

Figure S 16: Recyclability investigation for the photooxidation of 4-(methylthio)toluene to the corresponding sulfoxide. The reaction was performed under standard conditions (general procedure 9.1) in triplicates.

16. Comparison of Photocatalytic mCTA against Photocatalytic Nanoparticles

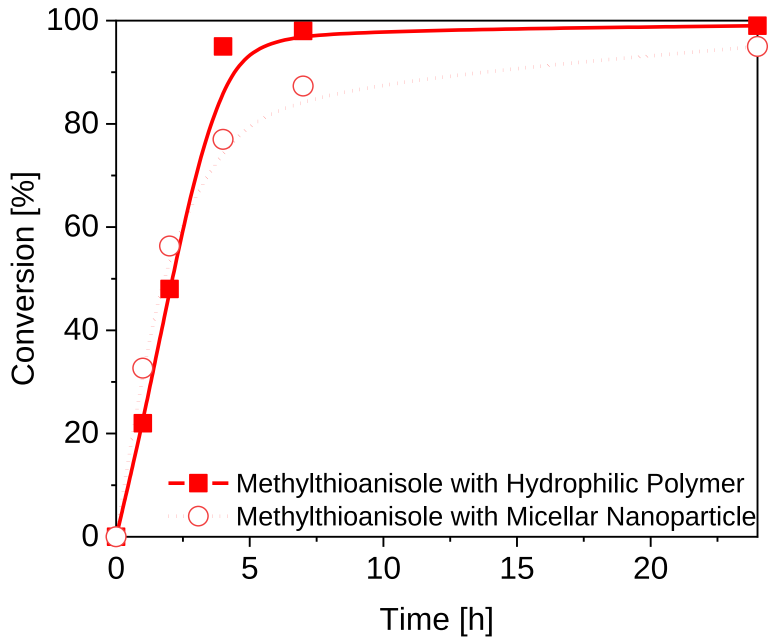

Figure S 17: Comparison of reactivity towards the sulfide oxidation of 4-(methylthio)toluene of photocatalytic, hydrophilic polymer (mCTA) and photocatalytic, amphiphilic polymeric nanoparticles.

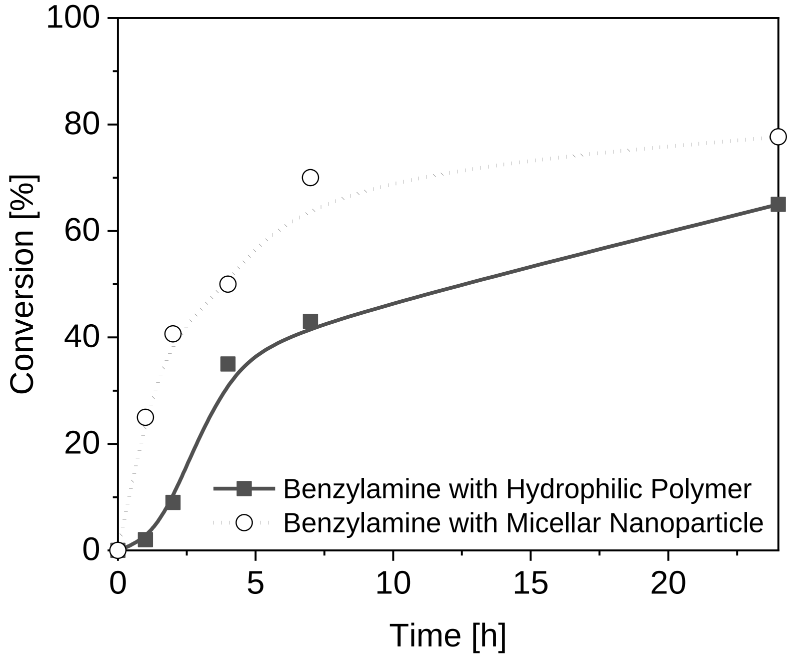

Figure S 18: Comparison of reactivity towards the imine formation of benzylamine of photocatalytic, hydrophilic polymer (mCTA) and photocatalytic, amphiphilic polymeric nanoparticles.

17. TEM Analysis

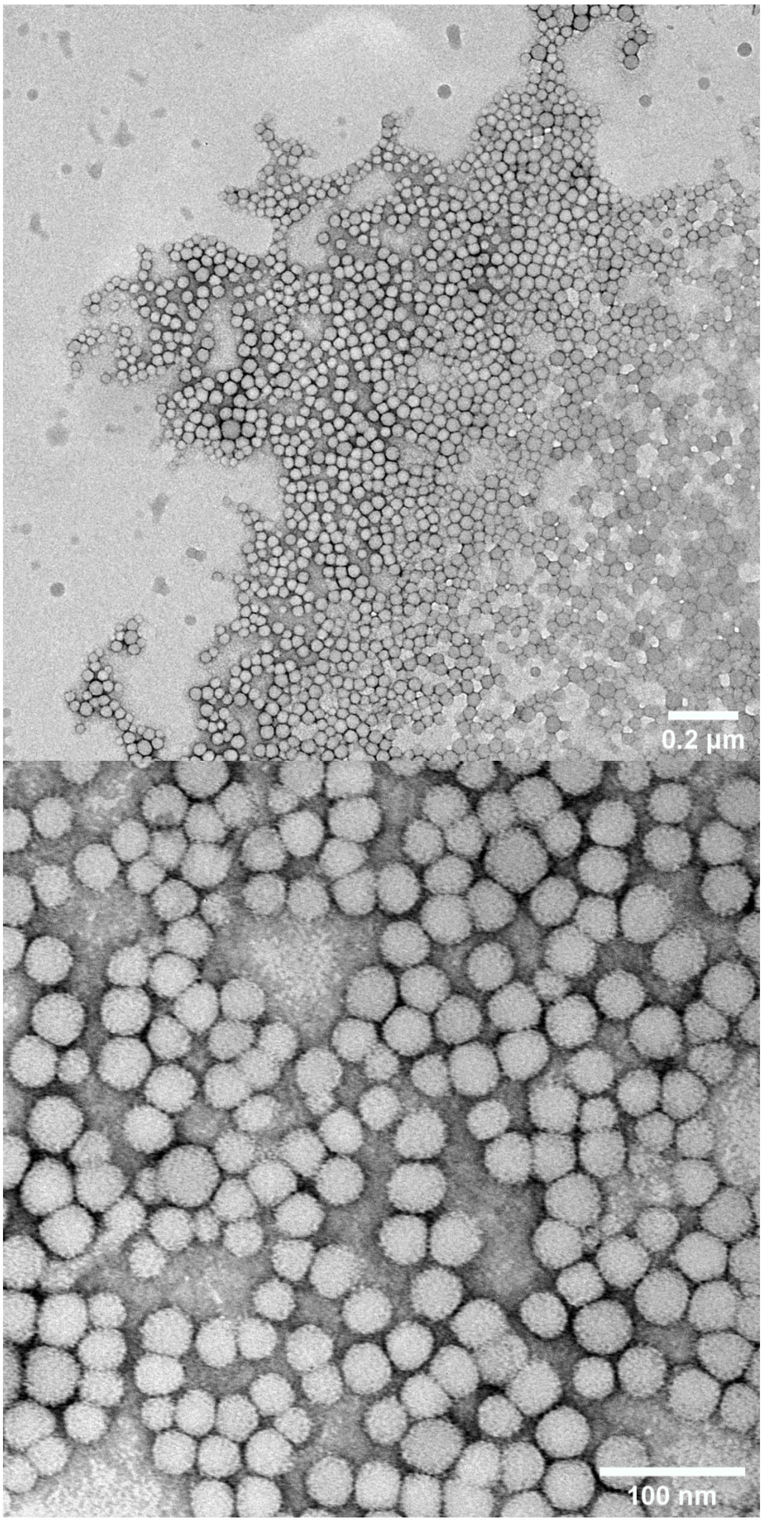

Figure S 19: TEM images of the hydrophilic localised photocatalyst, showing the particles at different magnification.

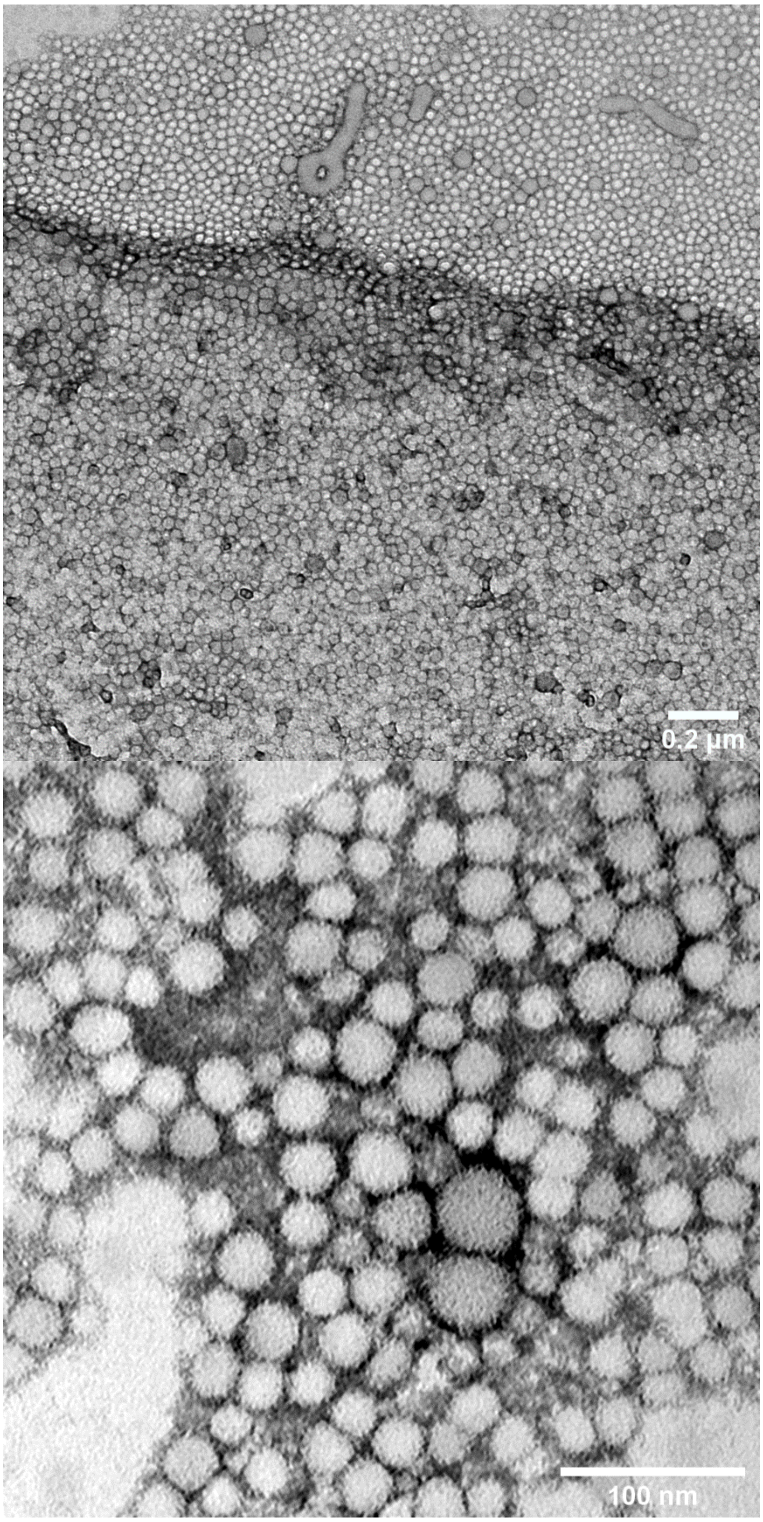

Figure S 20: TEM images of the hydrophobic localised photocatalyst, showing the particles at different magnification.

18. Experimental Section/Methods

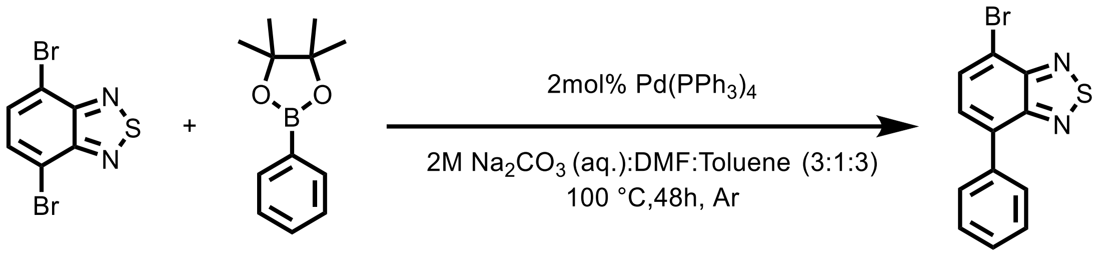

4-bromo-7-phenylbenzo[1,2,5]thiadiazole: Into a 150 ml Schlenk tube with a stir bar, 25 ml of a 2M Na_2CO_3(aq.) solution, 25 ml of Toluene and 9 ml of DMF were added inside. Subsequently, the solution was degassed with an argon stream for 15min, before adding 4,7-dibromobenzo[1,2,5]thiadiazole (1.5 Eq., 2.16 g, 7.35 mmol) and 4,4,5,5-tetramethyl-2-phenyl-1,3,2-dioxaborolane (1 Eq., 1 g, 4.90 mmol) and Tetrakis(triphenylphosphane)palladium (0.02 Eq., 113.25 mg, 98.00 µmol) in an argon counter stream. The reaction mixture was heated to 100 °C for 48 h with an attached reflux condenser under heavy stirring. After cooling down to room temperature, 30 ml of Milli-Q water were added, followed by dichloromethane extraction (4x 25 ml), washing by brine and drying over Na_2SO_4. After evaporation of all volatiles with the rotary evaporator, the crude mixture was purified using SiO_2 column chromatography (Gradient from 10% DCM: 90% Petrolether to 70% DCM: 30% Petrolether). A mixture of the product and biphenylbenzothiadiazole was obtained and used without further purification. (960 mg).

^{1}H NMR (300 MHz, $CDCl_3$, δ): 7.80 (m, 2H, Ar H), 7.60 (s, 2H; Ar H), 7.44 (m, 3H; Ar H);

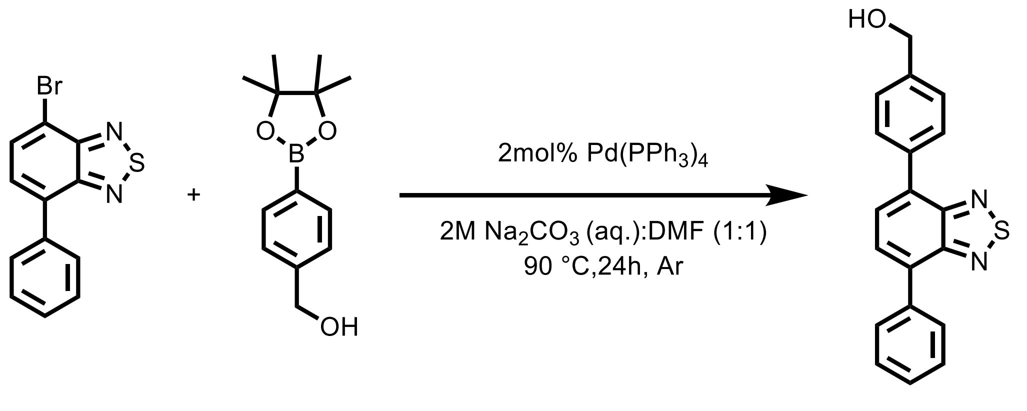

(4-(7-phenylbenzo[1,2,5]thiadiazol-4-yl)phenyl)methanol: Into a 150 ml Schlenk tube with a stir bar, 25 ml of a 2M Na_2CO_3(aq.) solution, 25 ml of Toluene and 9 ml of DMF were added inside. Subsequently, the solution was degassed with an argon stream for 15min, before adding 4-bromo-7-phenylbenzo[1,2,5]thiadiazole (1.0 Eq., 550 mg, 1.89 mmol) and (4-(4,4,5,5-tetramethyl-1,3,2-dioxaborolan-2-yl)phenyl)methanol (1.5 Eq., 663.31 mg, 2.83 mmol) and Tetrakis(triphenylphosphane)palladium (0.02 Eq., 43.66 mg, 37.78 µmol) in an argon counter stream. The reaction mixture was heated to 90 °C for 24 h with an attached reflux condenser under heavy stirring. After cooling down to room temperature, 20 ml of Milli-Q water were added, followed by dichloromethane extraction (3x 25 ml), washing by brine and drying over Na_2SO_4. After evaporation of all volatiles with the rotary evaporator, the crude mixture was purified using SiO_2 column chromatography (Column deprotonated with 10%NEt_3: 90%EtOAc; Gradient from 10% EtOAc: 90% Petrolether to 70% EtOAc: 30% Petrolether). The product was obtained as yellow crystals (253 mg, 42% yield).

^{1}H NMR (300 MHz, $CDCl_3$, δ): 8.01 (m, 4H, Ar H), 7.85 (s, 2H; Ar H), 7.59 (m, 4H; Ar H), 7.49 (m, 1H; Ar H), 4.81 (d, 2H; CH_2);

^{13}C NMR (101 MHz, $CDCl_3$, δ): 132.3 (2C; Ar C=N), 129.4 (Ar C), 129.3 (5C; Ar C), 128.1 (4C; Ar C), 126.9 (6C; Ar C), 64.7 (1C, C-OH);

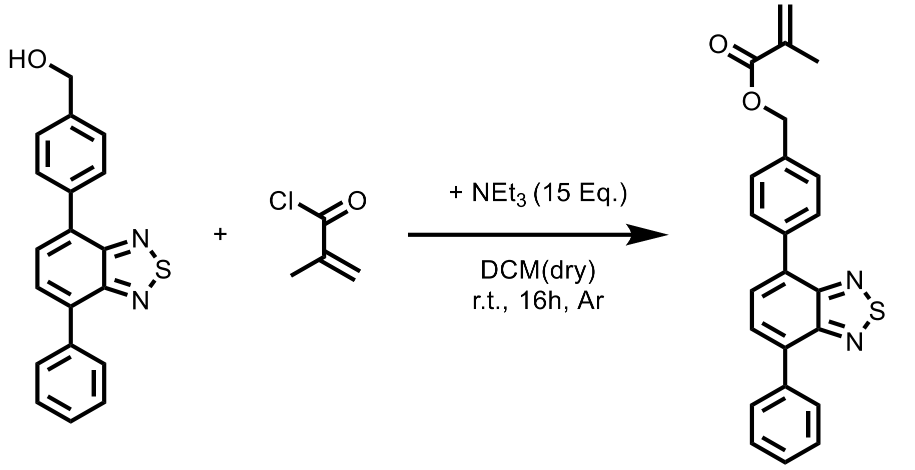

4-(7-phenylbenzo[1,2,5]thiadiazol-4-yl)benzyl methacrylate: Into an evacuated 50 ml Schlenk tube with a stir bar, 25 ml DCM (dry) were added inside. Subsequently, (4-(7-phenylbenzo[1,2,5]thiadiazol-4-yl)phenyl)methanol (1.0 Eq., 150 mg, 1.89 mmol) and Triethylamine (15 Eq., 984.99 µL, 28.35 mmol) were added in an argon counter stream. The reaction mixture was stirred for 30 min, before slowly adding methacryloyl chloride (1.2 Eq., 55 µL, 565.34 µmol) over 20 min. Afterwards, the mixture was kept under argon and stirring for 16 h. After that, 20 ml of Milli-Q water were added, followed by dichloromethane extraction (3x 25 ml), washing by brine and drying over Na_2SO_4. After evaporation of all volatiles with the rotary evaporator, the crude mixture was purified using SiO_2 column chromatography (Column deprotonated with 10%NEt_3: 90%EtOAc; Gradient from 10% EtOAc: 90% Petrolether to 70% EtOAc: 30% Petrolether). The product was obtained as bright yellow powder (134 mg, 73% yield).

^{1}H NMR (300 MHz, $CDCl_3$, δ): 7.98 (m, 4H, Ar H), 7.82 (s, 2H; Ar H), 7.61 (m, 4H; Ar H), 7.48 (m, 1H; Ar H), 4.81 (m, 2H; CH_2), 6.23 (s, 1H; CH_2), 5.64 (s, 1H; CH_2), 5.32 (s, 2H; CH_2), 2.03 (s, 3H; CH_3);

^{13}C NMR (101 MHz, $CDCl_3$, δ): 144.7 (1C; C=O), 136.5 (2C; Ar C=N), 129.4 (2C; Ar C), 129.3 (3C; Ar C), 128.5 (3C; Ar C), 128.1 (6C; Ar C), 125.5 (2C; Ar C), 115.1 (1C; C4), 113.8 (1C; C2), 65.9 (1C, C2), 18.1 (1C; C1);

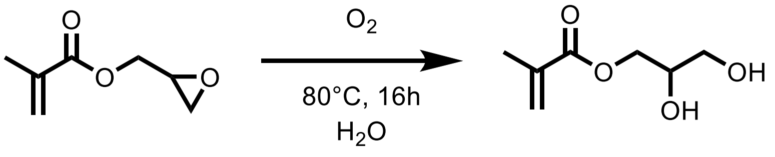

2,3-dihydroxypropyl methacrylate: In a 50 ml roundbottom flask equipped with a stir bar, a 20 wt% solution of glycidyl methacrylate (5g) in Milli-Q water(20 ml) were gassed with oxygen for 30 min. Afterwards the biphasic solution was reacted for 16 h at 80 °C under heavy stirring. This crude aqueous solution was used without further purification for the following macro-CTA generation.

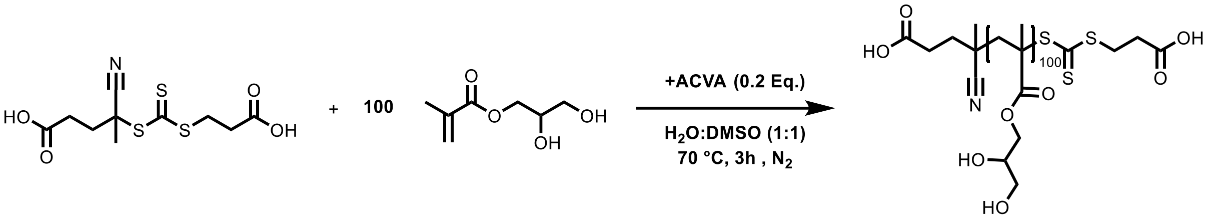

Hydrophilic macro-chain transfer agent (mCTA): Into a 20 mL screw-cap vial equipped with a stir bar, freshly synthesized 2,3-dihydroxypropyl methacrylate solution (2.5 mL, 20 wt/vol%, aqueous) was transferred. After addition of DMSO (2.5 mL), 4,4'-azobis(4-cyanopentanoic acid) (0.2 Eq., 1.75 mg, 6.24 µmol) and 4-(((2-carboxyethyl)thio)carbonothioyl)thio)-4-cyanopentanoic acid (1 Eq., 9.6 mg, 31.22 µmol), the crude mixture was degassed with N_2 for 20 min with a light pressure. Afterwards, the solution was reacted under heavy stirring for 3 h at 70 °C. After cooling down to room temperature, the solution was dialyzed (3x EtOH:H_2O, 1:1, exchange after 6 h, 12 h and 24 h, followed by 3x Water, 100%, exchanged every day),followed by lyophilization until dry. The product was obtained as a colorless, loose solid.

^{1}H NMR (300 MHz, DMSO-d_6, δ): 4.91 (br, 1H, OH), 4.67 (br, 1H; OH), 3.90 (br, 1H;), 3.68 (br, 2H; CH), 3.52 (br, 1H; CH_2), 3.38 (br, 2H; CH_2), 1.78 (br, 2H; CH_2), 0.85 (m, 3H; CH_3);

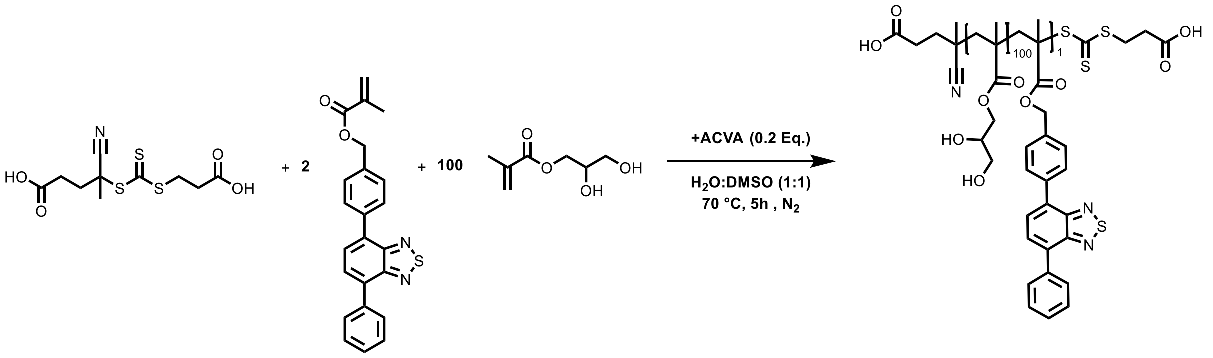

Copolymerized, hydrophilic located photocatalyst macro-chain transfer agent (mCTA): Into a 20 mL screw-cap vial equipped with a stir bar, freshly synthesized 2,3-dihydroxypropyl methacrylate solution (2.5 mL, 20 wt/vol%, aqueous) was transferred. After addition of DMSO (2.5 mL), 4,4'-azobis(4-cyanopentanoic acid) (0.2 Eq., 1.75 mg, 6.24 µmol), 4-(7-phenylbenzo[1,2,5]thiadiazol-4-yl)benzyl methacrylate (2 Eq., 24.13 mg, 62.43 µmol) and 4-((((2-carboxyethyl)thio)carbonothioyl)thio)-4-cyanopentanoic acid (1 Eq., 9.6 mg, 31.22 µmol), the crude mixture was degassed with N_2 for 20 min with a light pressure. Afterwards, the solution was reacted under heavy stirring for 5 h at 70 °C. After cooling down to room temperature, the solution was dialyzed (3x EtOH:H_2O, 1:1, exchange after 6 h, 12 h and 24 h, followed by 3x Water, 100%, exchanged every day) followed by lyophilization until dry. The product was obtained as a slightly yellow, loose solid.

^{1}H NMR (300 MHz, DMSO-d_6, δ): 8.00 (m, 5H; Ar H), 7.41 (m, 6H; Ar H), 4.91 (br, 1H, OH), 4.67 (br, 1H; OH), 3.90 (br, 1H;), 3.68 (br, 2H; CH), 3.52 (br, 1H; CH_2), 3.38 (br, 2H; CH_2), 1.78 (br, 2H; CH_2), 0.85 (m, 3H; CH_3);

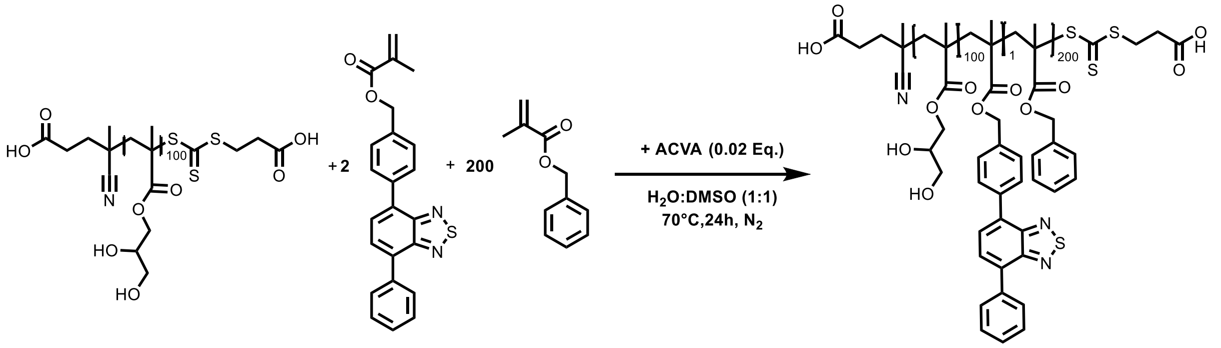

Copolymerization of photocatalyst and hydrophobic block with hydrophilic mCTA:

Into a 20 mL screw-cap vial equipped with a stir bar, hydrophilic mCTA $P(GMA)_{100}$ (1 Eq., 300 mg, 18.38 µmol) was transferred. After addition of H_2O (3 mL) and DMSO (3 mL), 4,4'-azobis(4-cyanopentanoic acid) (0.2 Eq., 1.03 mg, 3.68 µmol), 4-(7-phenylbenzo[1,2,5]thiadiazol-4-yl)benzyl methacrylate (2 Eq., 14.20 mg, 36.76 µmol) and Benzyl methacrylate (200 Eq., 647.68 mg, 3.68 mmol) the crude mixture was degassed with N_2 for 20 min with a light pressure. Afterwards, the solution was reacted under heavy stirring for 24 h at 70 °C, forming an opaque dispersion. After cooling down to room temperature, the solution was dialyzed (3x EtOH:H_2O, 1:1, exchange after 6 h, 12 h and 24 h, followed by 3x Water, 100%, exchanged every day), followed by lyophilization until dry. The product was obtained as a slightly yellow, loose solid.

^{1}H NMR (300 MHz, DMSO-*d*$_6$, δ): 7.52 (m, 11H; Ar H), 7.25 (br, 5H; Ar H), 4.86 (br, 1H, OH), 4.67 (br, 1H; OH), 3.93 (br, 1H;), 3.68 (br, 2H; CH), 3.52 (br, 1H; CH_2), 3.38 (br, 2H; CH_2), 1.78 (br, 2H; CH_2), 0.80 (m, 3H; CH_3);

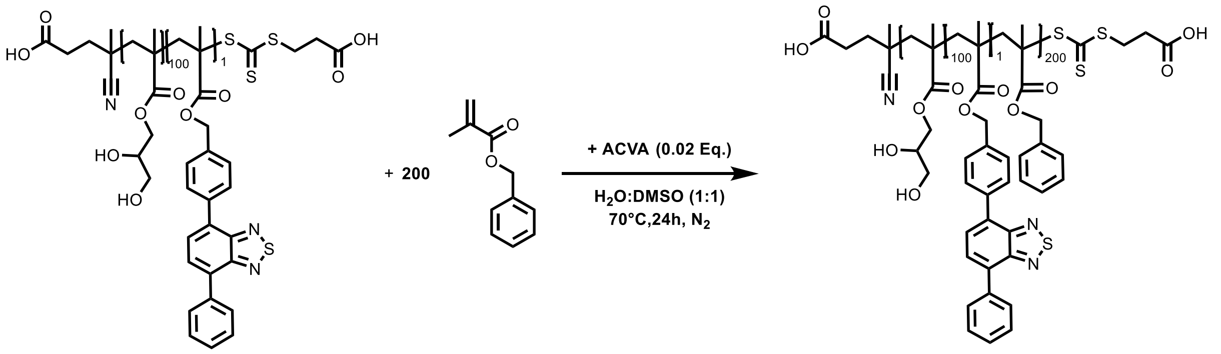

Copolymerization of the hydrophobic block with hydrophilic, photoctalytic mCTA:

Into a 20 mL screw-cap vial equipped with a stir bar, hydrophilic mCTA $P(GMA)_{100}$ $(MaBTPh)_1$(1 Eq., 300 mg, 17.95 µmol) was transferred. After addition of H_2O (3 mL) and DMSO (3 mL), 4,4'-azobis(4-cyanopentanoic acid) (0.2 Eq., 1.01 mg, 3.59 µmol) and Benzyl methacrylate (200 Eq., 632.70 mg, 3.59 mmol) the crude mixture was degassed with N_2 for 20 min with a light pressure. Afterwards, the solution was reacted under heavy stirring for 24 h at 70 °C, forming an opaque dispersion. After cooling down to room temperature, the solution was dialyzed (3x EtOH:H_2O, 1:1, exchange after 6 h, 12 h and 24 h, followed by 3x Water, 100%, exchanged every day), followed by lyophilization until dry. The product was obtained as a slightly yellow, loose solid.

^{1}H NMR (300 MHz, DMSO-*d*$_6$, δ): 7.52 (m, 11H; Ar H), 7.25 (br, 5H; Ar H), 4.86 (br, 1H, OH), 4.67 (br, 1H; OH), 3.93 (br, 1H;), 3.68 (br, 2H; CH), 3.52 (br, 1H; CH_2), 3.38 (br, 2H; CH_2), 1.78 (br, 2H; CH_2), 0.80 (m, 3H; CH_3);

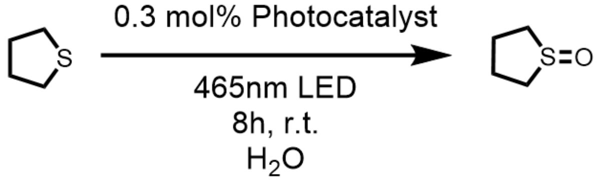

Tetrahydrothiophene 1-oxide

The synthesis of tetrahydrothiophene 1-oxide was performed according to the general procedure for photocatalytic sulfide oxidations, explained in SI section 9.1. The purification of the compound was performed by flash column chromatography (Hexane: EtOAc, 9:1) with potassium permanganate staining.

^{1}H NMR (300 MHz, $CDCl_3$, δ): 2.96 (m, 2H, CH_2), 2.15 (m, 2H; CH_2);

^{13}C NMR (101 MHz, $CDCl_3$, δ): 51.2 (2C; C2), 22.8 (2C; C2);

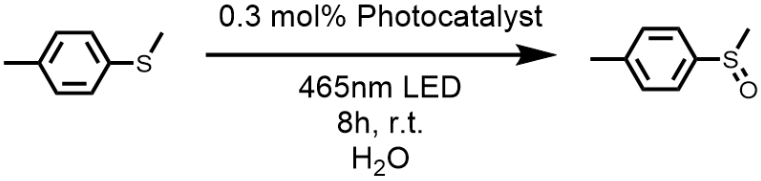

1-methyl-4-(methylsulfinyl)benzene

The synthesis of 1-methyl-4-(methylsulfinyl)benzene was performed according to the general procedure for photocatalytic sulfide oxidations, explained in SI section 9.1. The purification of the compound was performed by flash column chromatography (Hexane: EtOAc, 8:2).

^{1}H NMR (300 MHz, $CDCl_3$, δ): 7.46 (m, 2H, Ar H), 7.28 (m, 2H, Ar H), 2.65 (s, 3H; CH_3), 2.35 (s, 3H; CH_3);

^{13}C NMR (101 MHz, $CDCl_3$, δ): 130.1 (2C; Ar C), 123.7 (2C; Ar C), 118.2 (2C; Ar C), 43.9 (1C; C1), 23.4 (1C; C1);

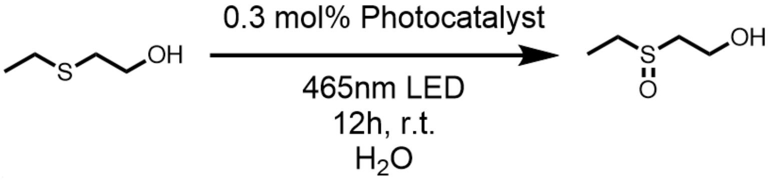

2-(ethylsulfinyl)ethan 1-ol

The synthesis of 2-(ethylsulfinyl)ethan 1-ol was performed according to the general procedure for photocatalytic sulfide oxidations, explained in SI section 9.1. The purification of the compound was performed by flash column chromatography (Hexane: EtOAc, 8:2).

^{1}H NMR (300 MHz, $CDCl_3$, δ): 4.04 (t, 2H, CH_2), 3.13 (t, 2H, CH_2), 3.04 (m, 2H, CH_2), 2.44 (s, 1H; OH), 1.35 (m, 3H, CH_3);

^{13}C NMR (101 MHz, $CDCl_3$, δ): 56.4 (1C; C2), 54.1 (1C; C2), 48.9 (1C; C2), 6.5 (1C; C1);

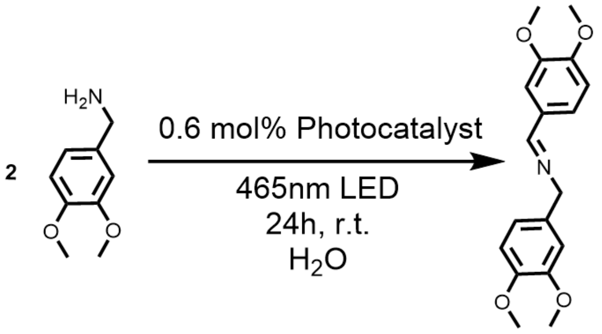

N-(3,4-dimethoxybenzyl)-1-(3,4-dimethoxyphenyl)methanimine

The synthesis of N-(3,4-dimethoxybenzyl)-1-(3,4-dimethoxyphenyl)methanimine was performed according to the general procedure for photocatalytic imine formation, explained in SI section 9.2. The purification of the compound was performed by flash column chromatography (Hexane: EtOAc, 6:4).

^{1}H NMR (300 MHz, $CDCl_3$, δ): 8.27 (s, 1H, N=CH), 7.47 (m, 1H, Ar H), 7.19 (m, 1H, Ar H), 6.85 (m, 4H; Ar H), 4.73 (s, 2H, CH_2), 4387 (dd, 12H, CH_3);

^{13}C NMR (101 MHz, $CDCl_3$, δ): 161.3 (1C; C=N), 151.4 (1C; Ar H), 149.3 (1C; Ar H), 149.0 (1C; Ar H), 148.2 (1C; Ar H), 132.1 (1C; Ar H), 129.46 (1C; Ar H), 123.3 (1C; Ar H), 120.2 (1C; Ar H), 111.5 (1C; Ar H), 111.3 (1C; Ar H), 110.4 (1C; Ar H), 108.9 (1C; Ar H), 64.7 (1C; CH_2) , 55.9 (4C; CH_3);

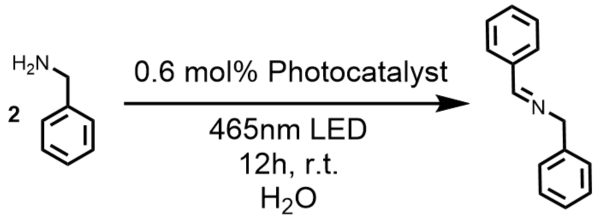

N-benzyl-1-phenylmethanimine

The synthesis of N-benzyl-1-phenylmethanimine was performed according to the general procedure for photocatalytic imine formation, explained in SI section 9.2. The purification of the compound was performed by flash column chromatography (Hexane: EtOAc, 6:4).

^{1}H NMR (300 MHz, $CDCl_3$, δ): 8.26 (s, 1H, N=CH), 7.67 (m, 2H, Ar H), 7.29 (m, 2H, Ar H), 7.22 (m, 4H; Ar H), 7.16 (m, 2H, Ar H), 4.70 (s, 2H, CH_2);

^{13}C NMR (101 MHz, $CDCl_3$, δ): 162.0 (1C; C=N), 139.4 (1C; Ar H), 136.3 (1C; Ar H), 130.8 (1C; Ar H), 128.7 (2C; Ar H), 128.5 (2C; Ar H), 128.35 (2C; Ar H), 128.1 (2C; Ar H), 127.1 (1C; Ar H), 65.1 (1C; CH_2);

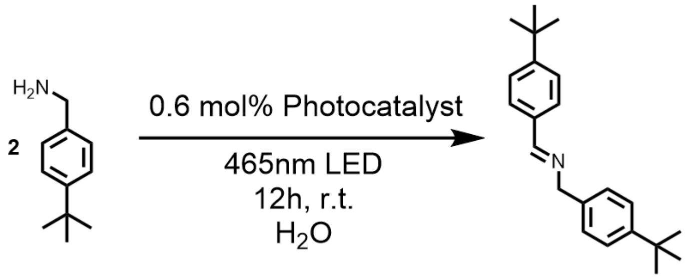

N-(4-(tert-butyl)benzyl)-1-(4-(tert-butyl)phenyl)methanimine

The synthesis of N-(4-(tert-butyl)benzyl)-1-(4-(tert-butyl)phenyl)methanimine was performed according to the general procedure for photocatalytic imine formation, explained in SI section 9.2. The purification of the compound was performed by flash column chromatography (Hexane: EtOAc, 6:4).

^{1}H NMR (300 MHz, $CDCl_3$, δ): 8.26 (s, 1H, N=CH), 7.62 (m, 2H, Ar H), 7.36 (m, 2H, Ar H), 7.27 (m, 2H; Ar H), 7.19 (m, 2H, Ar H), 4.70 (s, 2H, CH_2), 1.23 (m, 18H, CH_3);

^{13}C NMR (101 MHz, $CDCl_3$, δ): 161.7 (1C; C=N), 154.1 (1C; Ar H), 149.8 (1C; Ar H), 136.4 (1C; Ar H), 133.7 (1C; Ar H), 128.1 (1C; Ar H), 127.7 (3C; Ar H), 125.5 (4C; Ar H), 64.8 (1C; CH_2), 34.9 (1C; C4), 34.4 (1C; C4), 31.2 (6C; C1);

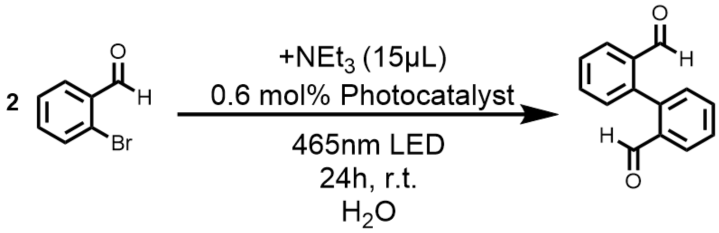

[1,1'-biphenyl]-2,2'-dicarbaldehyde

The synthesis of [1,1'-biphenyl]-2,2'-dicarbaldehyde was performed according to the general procedure for photocatalytic radical C-C coupling, explained in SI section 9.3. The purification of the compound was performed by flash column chromatography (Hexane: EtOAc, 6:4).

^{1}H NMR (300 MHz, $CDCl_3$, δ): 9.76 (s, 2H; COH), 7.97 (m, 2H; Ar H), 7.55 (m, 4H; Ar H), 7.29 (m, 2H; Ar H);

^{13}C NMR (101 MHz, $CDCl_3$, δ): 191.0 (2C; C=O), 141.2 (2C; Ar), 134.6 (2C; Ar), 133.4 (2C; Ar), 131.7 (2C; Ar), 128.8 (2C; Ar), 128.5 (2C; Ar);

19. Investigation of the Photocatalytic Behaviour According to Reaction Condition Variation by Means of Sulfide Oxidation Reaction.[a]

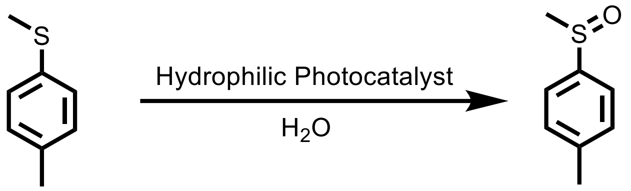

No	Reaction Condition Variation	Conversion [%][b]
1	-	91
2	Degassed by N_2	28
3	No light	0
4	No photocatalyst	0
5	Non-photocatalytic polymer	0
6	0.6 mol% photocatalyst + 10 mM substrate	89
7	0.3 mol% photocatalyst + 50 mM substrate	83
8	Reaction time 8 h	89
9	Additional Acetonitrile (15 µL)	89
10	Additional Acetonitrile (20 µL)	89
11	Additional NEt_3 (15 µL)	78
12	Additional NEt_3 + Acetonitrile (each 15µL)	51
13	Additional Hexafluoroisopropanol (15 µL)	90
14	Additional Hexafluorobenzene (15 µL)	90
15	Additional *deuterated*-Dichloromethane (15 µL)	89
16	Additional Toluene (15 µL)	89
17	Reduced light source (4.08 W total, 0.68 W per LED)	89

[a] **Conditions:** Methyl *p*-tolyl sulfane (20 µmol), hydrophilic photocatalyst polymer (0.096 µmol, 0.3 mol% photocatalyst), dispersed in H_2O (2 mL, sonicated 20 min), 15 °C, 7.14 W, 465 nm, 16 h. [b] Calculated from GC measurements by comparison of peak intensity.

20. Investigation of the Photocatalytic Behaviour According to Reaction Condition Variation by Means of Imine Formation Reaction.[a]

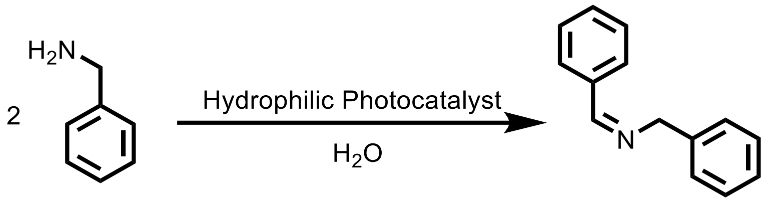

No	Reaction Condition Variation	Conversion [%][b]
1	-	79
2	Degassed by N_2	78
3	No light	<1%
4	No photocatalyst	6
5	Non-photocatalytic polymer	1
6	0.3 mol% photocatalyst + 10 mM substrate	35
7	0.3 mol% photocatalyst + 50 mM substrate	29
8	Reaction time 8 h	71
9	Additional Acetonitrile (15 µL)	76
10	Additional Acetonitrile (20 µL)	69
11	Additional NEt_3 (15 µL)	10
12	Additional NEt_3 + Acetonitrile (each 15µL)	16
13	Additional Hexafluoroisopropanol (15 µL)	11
14	Additional Hexafluorobenzene (15 µL)	62
15	Additional *deuterated*-Dichloromethane (15 µL)	63
16	Additional Toluene (15 µL)	41
17	Reduced light source (4.08 W total, 0.68 W per LED)	56

[a] **Conditions:** Benzyl amine (13.5 µmol), hydrophilic photocatalyst polymer (0.192 µmol, 0.6 mol% photocatalyst), dispersed in H_2O (2 mL, sonicated 20 min), 15 °C, 7.14 W, 465 nm, 16 h. [b] Calculated from GC measurements by comparison of peak intensity.

21. Investigation of the Photocatalytic Behaviour According to Reaction Condition Variation by Means of Aromatic, Radical C-C Coupling Reaction.[a]

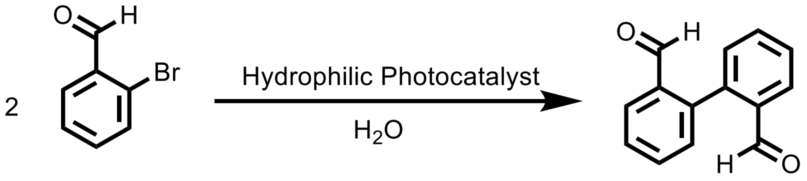

No	Reaction Condition Variation	Conversion [%][b]
1	-	0
0	Degassed by N_2	0
3	No light	0
4	No photocatalyst	0
5	Non-photocatalytic polymer +NEt_3 (15µL)	0
6	0.6 mol% photocatalyst + 10 mM substrate	0
7	0.3 mol% photocatalyst + 50 mM substrate	0
8	Reaction time 8 h	0
9	Additional Acetonitrile (15 µL)	4
10	Additional Acetonitrile (20 µL)	5
11	Additional NEt_3 (15 µL)	13
12	Additional NEt_3 + Acetonitrile (each 15µL)	51
13	Additional NEt_3 + Hexafluoroisopropanol (15 µL)	34
14	Additional NEt_3 + Hexafluorobenzene (15 µL)	25
15	Additional NEt_3 + *deuterated*-Dichloromethane (15 µL)	18
16	Additional NEt_3 + Toluene (15 µL)	16
17	Reduced light source (4.08 W total, 0.68 W per LED)	0

[a] **Conditions:** 2-Bromobenzaldehyde (20 µmol), hydrophilic photocatalyst polymer (0.096 µmol, 0.3 mol% photocatalyst), dispersed in H_2O (2 mL, sonicated 20 min), 15 °C, 7.14 W, 465 nm, 16 h. [b] Calculated from GC measurements by comparison of peak intensity.

22. Appendix

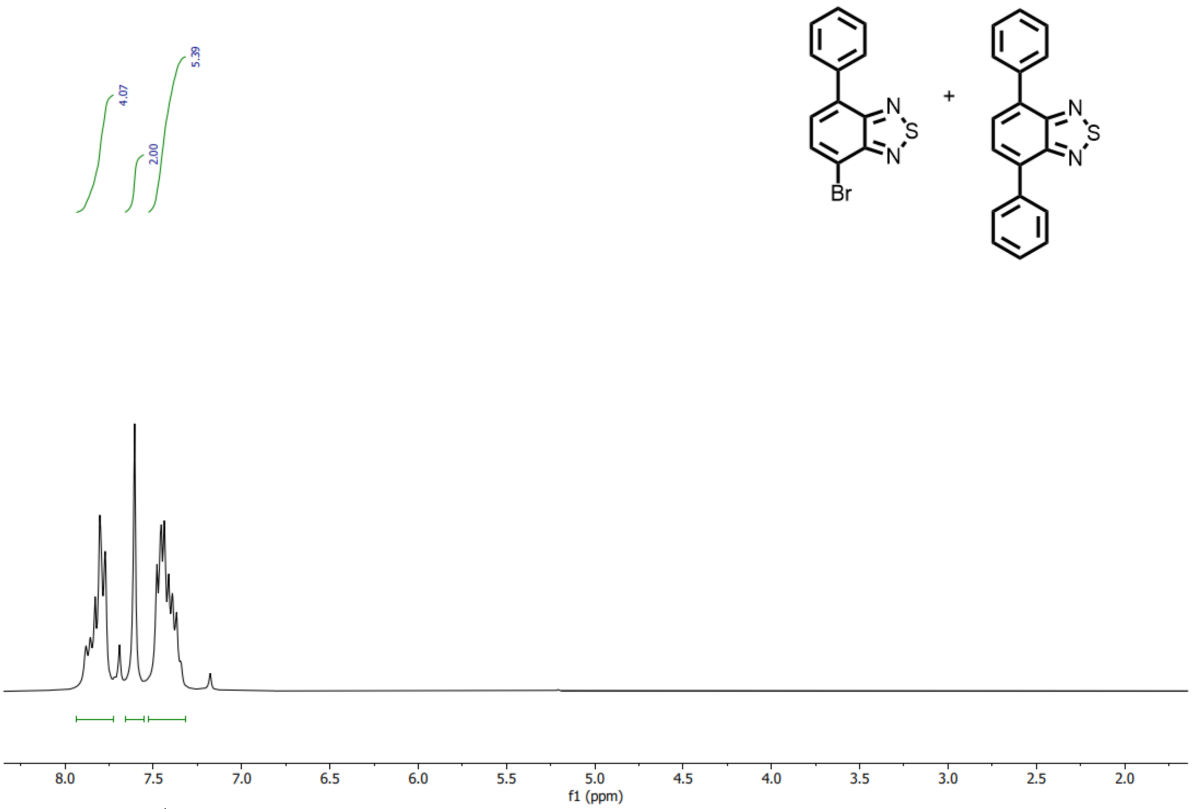

Figure S 21: ^{1}H-NMR spectrum of the reaction for 4-bromo-7-phenylbenzo[1,2,5]thiadiazole, giving a crude mixture of the above presented structures.

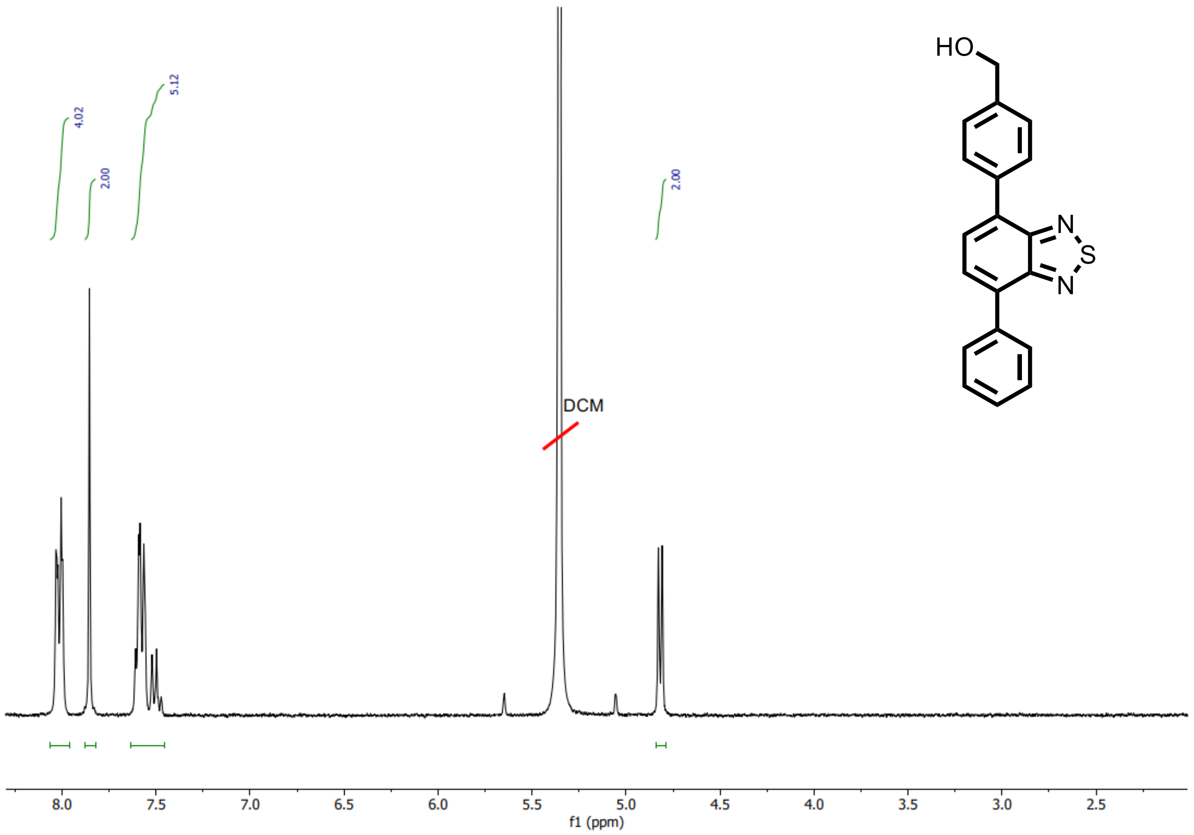

Figure S 22: ^{1}H-NMR spectrum of (4-(7-phenylbenzo[1,2,5]thiadiazol-4-yl)phenyl)methanol.

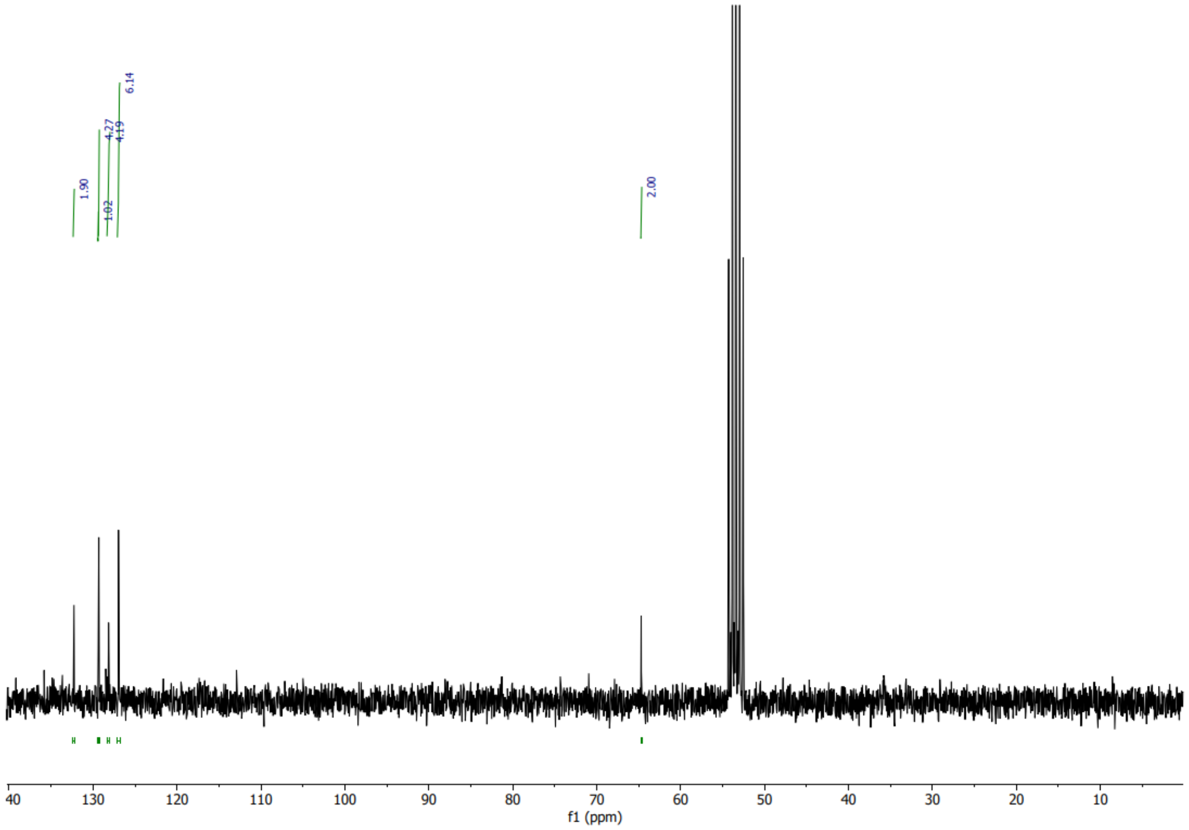

Figure S 23: ^{13}C-NMR spectrum of (4-(7-phenylbenzo[1,2,5]thiadiazol-4-yl)phenyl)methanol.

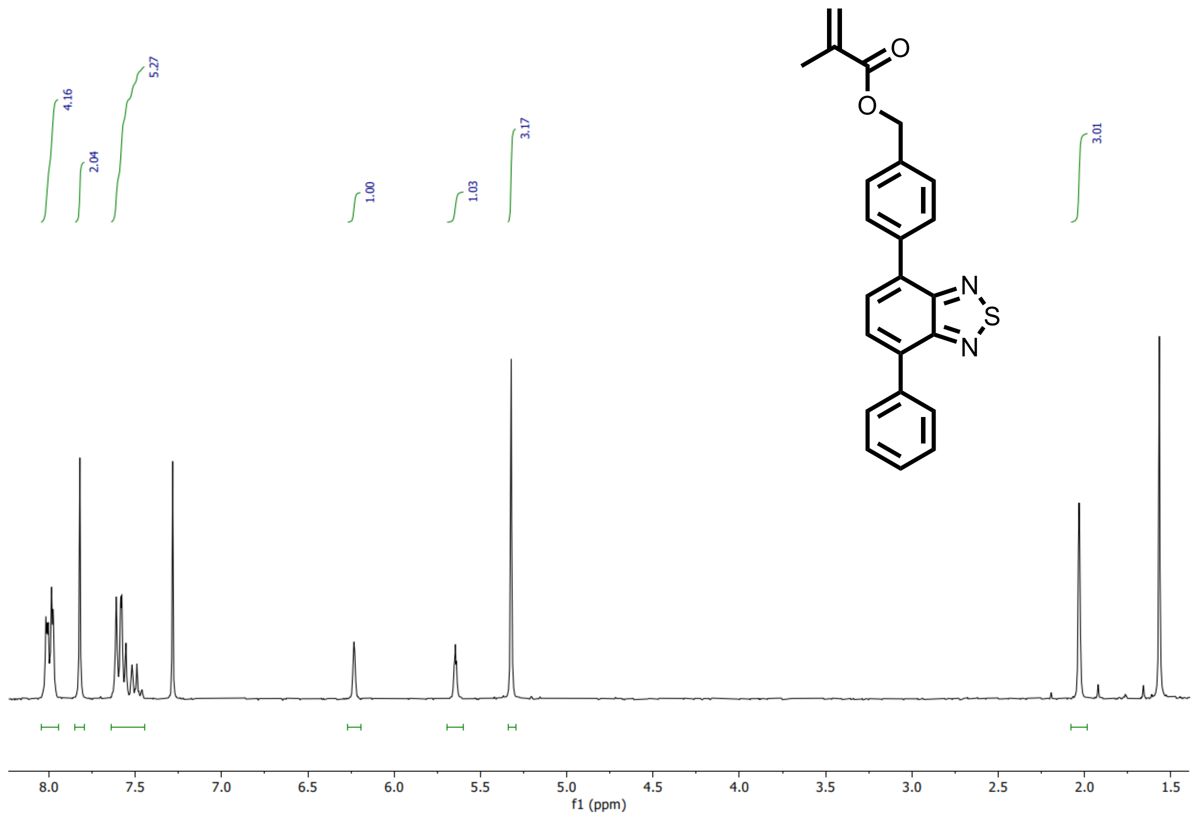

Figure S 24: ^{1}H-NMR spectrum of 4-(7-phenylbenzo[1,2,5]thiadiazol-4-yl)benzyl methacrylate.

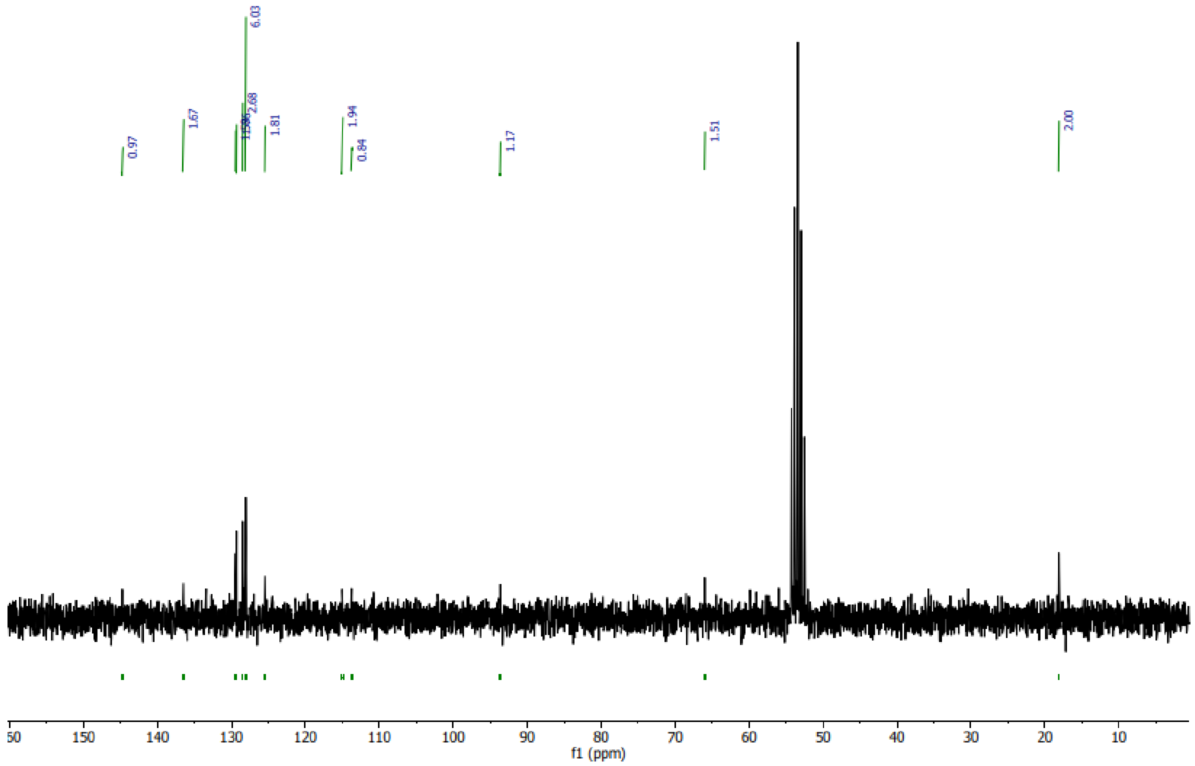

Figure S 25: ^{13}C-NMR spectrum of 4-(7-phenylbenzo[1,2,5]thiadiazol-4-yl)benzyl methacrylate.

Polymers

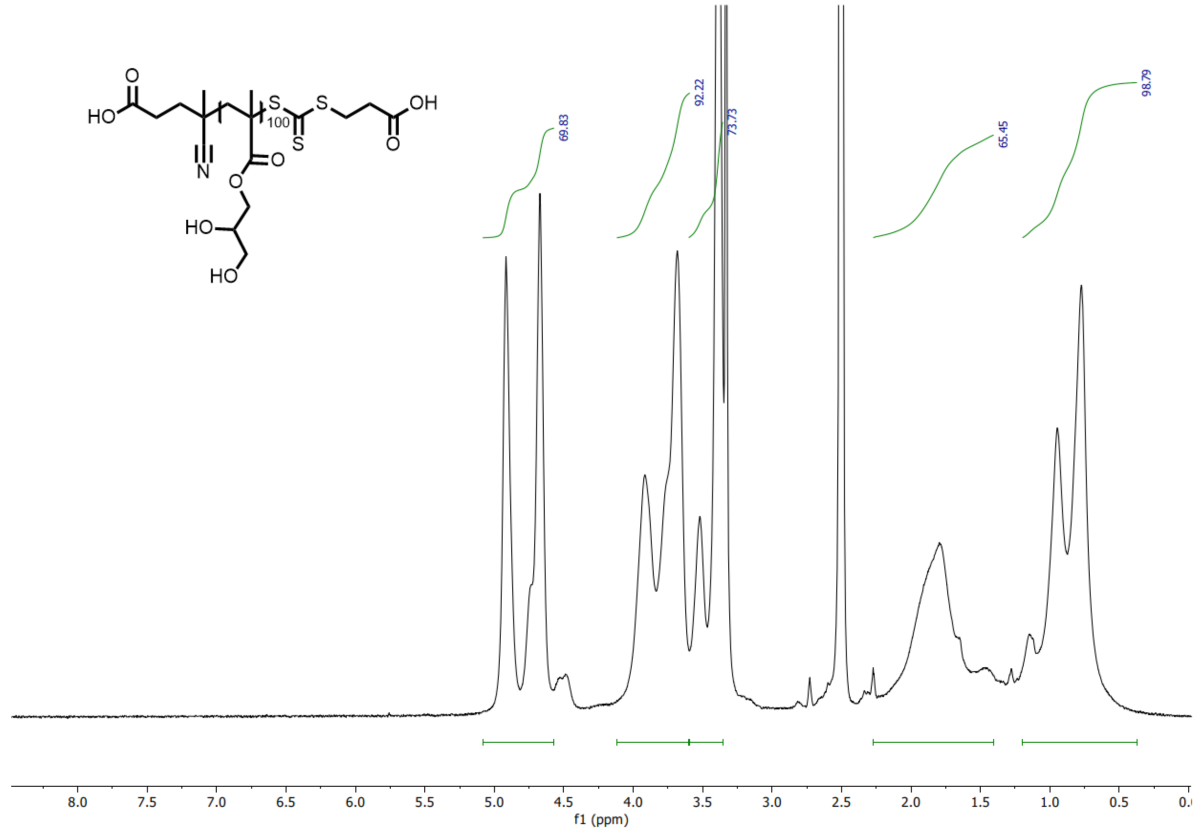

Figure S 26: ^{1}H-NMR spectrum of the hydrophilic mCTA P(GMA)$_{100}$.

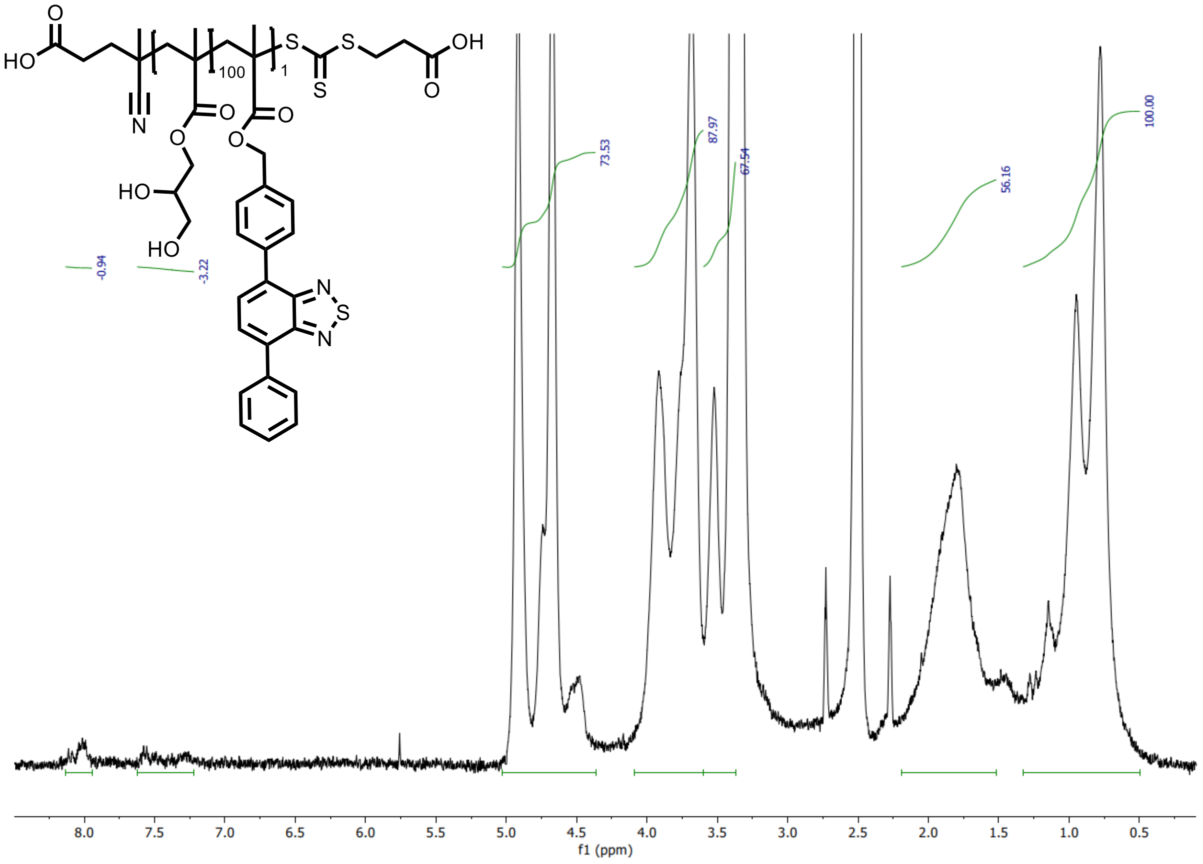

Figure S 1: ^{1}H-NMR spectrum of the hydrophilic mCTA with photocatalyst P(GMA)$_{100}$(BTP)$_{1}$.

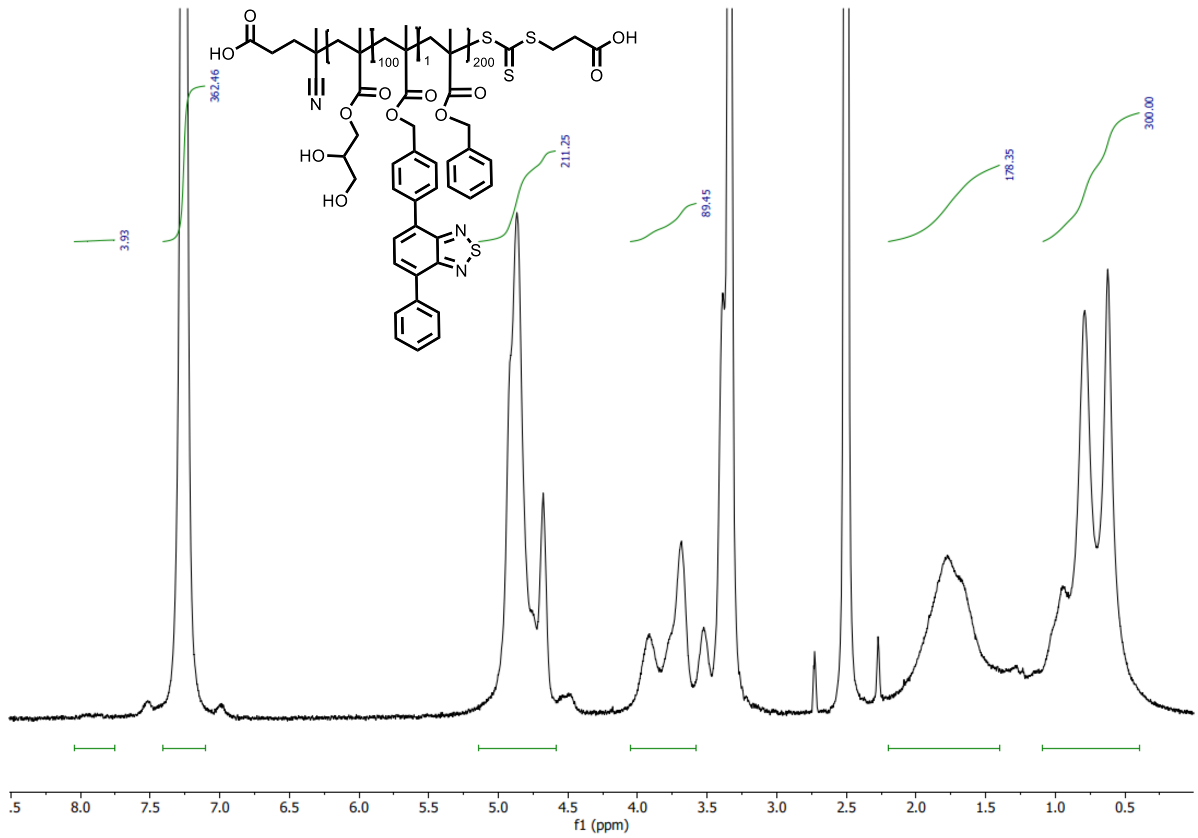

Figure S 28: ^{1}H-NMR spectrum of the amphiphilic block-copolymer with hydrophilic photocatalyst P(GMA)$_{100}$(BTP)$_1$P(BzMA)$_{200}$.

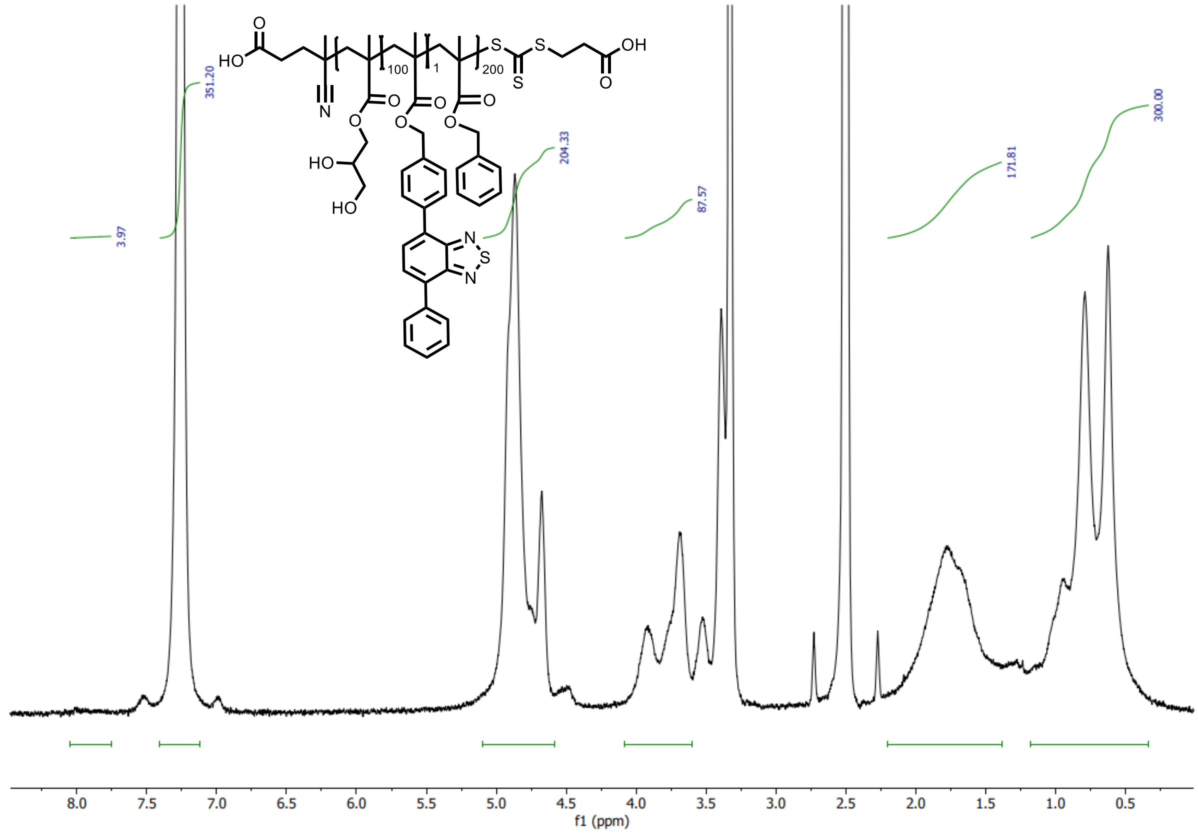

Figure S 29: ^{1}H-NMR spectrum of the amphiphilic block-copolymer with hydrophobic photocatalyst P(GMA)$_{100}$(BTP)$_1$P(BzMA)$_{200}$.

Substrate Conversions

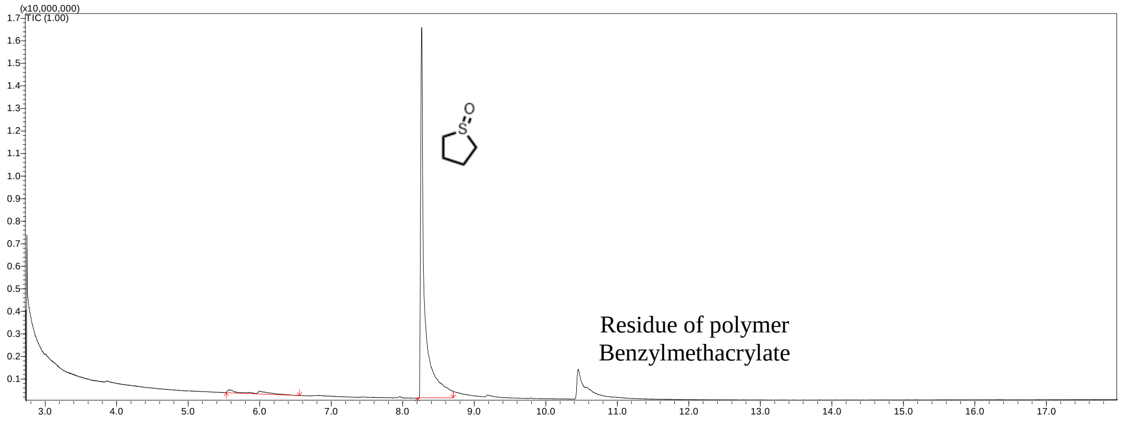

Figure S 30: GC/MS retention time diagram, showing the formation of tetrahydrothiophene 1-oxide and trace amounts of the starting material tetrahydrothiophene.

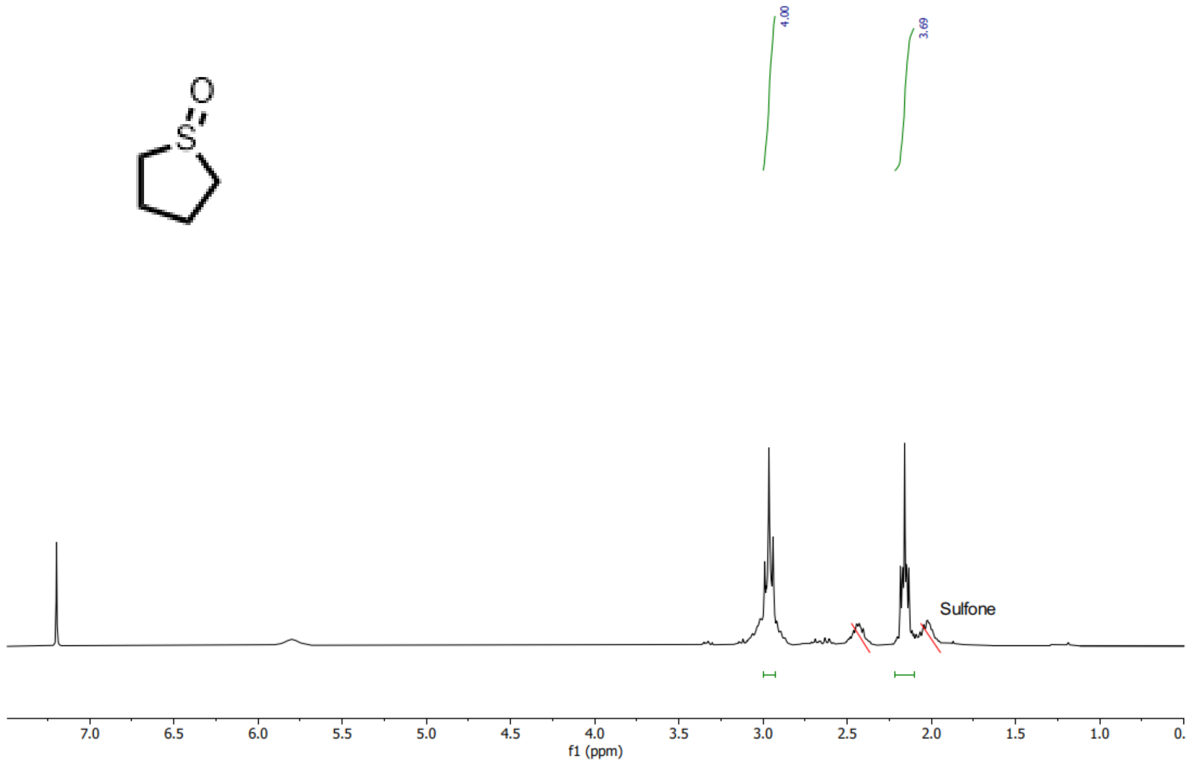

Figure S 31: ^{1}H-NMR spectrum of tetrahydrothiophene 1-oxide.

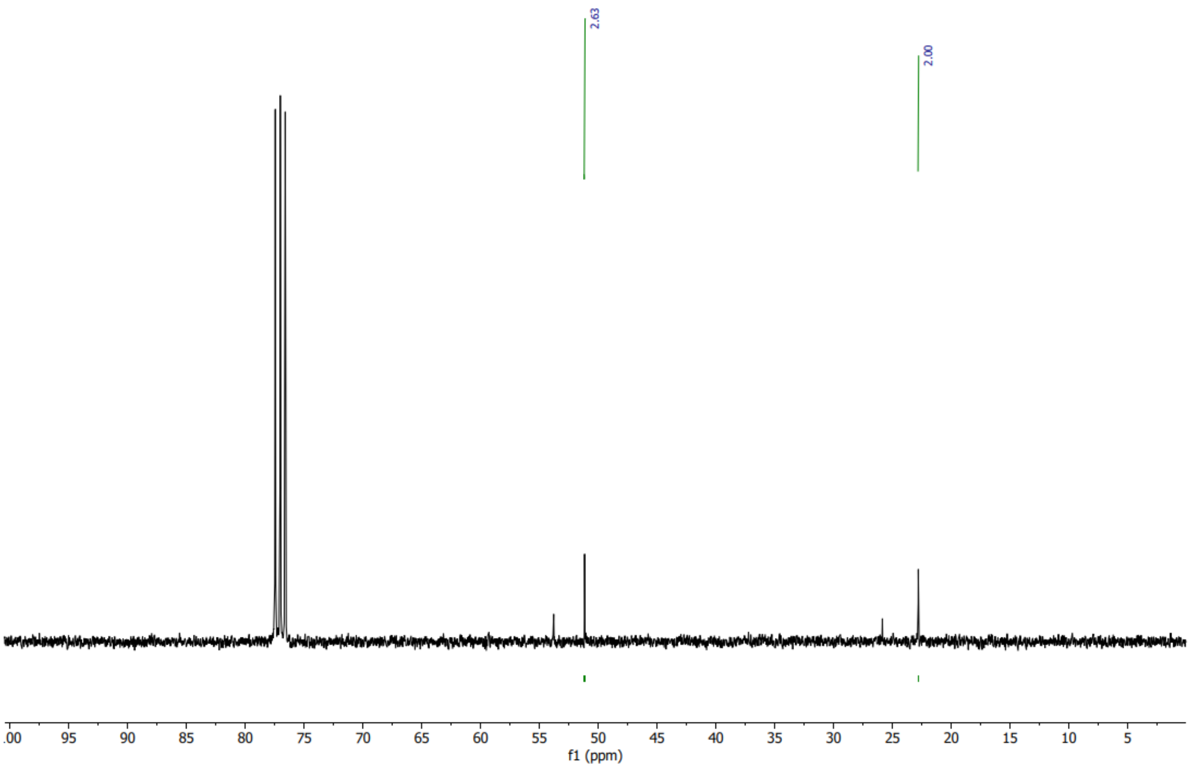

Figure S 32: ^{13}C-NMR spectrum of tetrahydrothiophene 1-oxide.

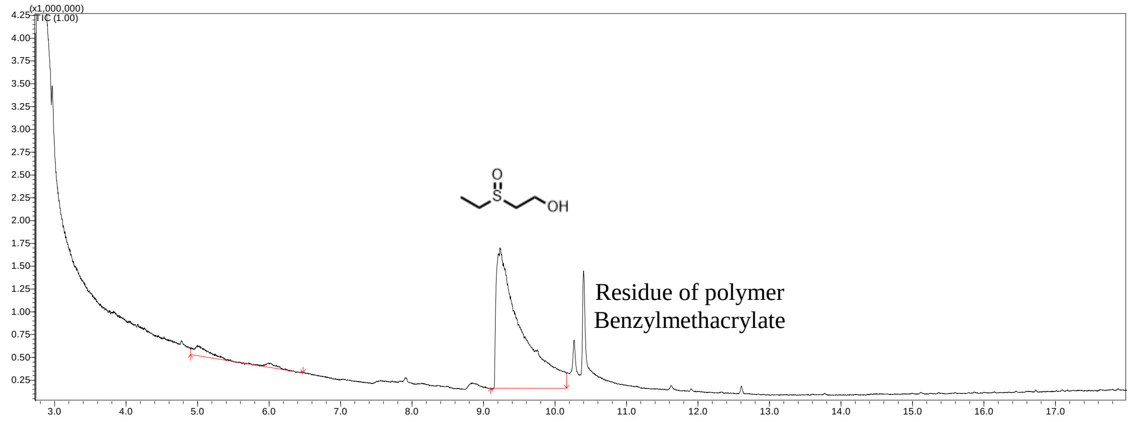

Figure S 33: GC/MS retention time diagram, showing the formation of 2-(ethylsulfinyl)ethan 1-ol and trace amounts of the starting material 2-(ethylthio)ethan-1-ol.

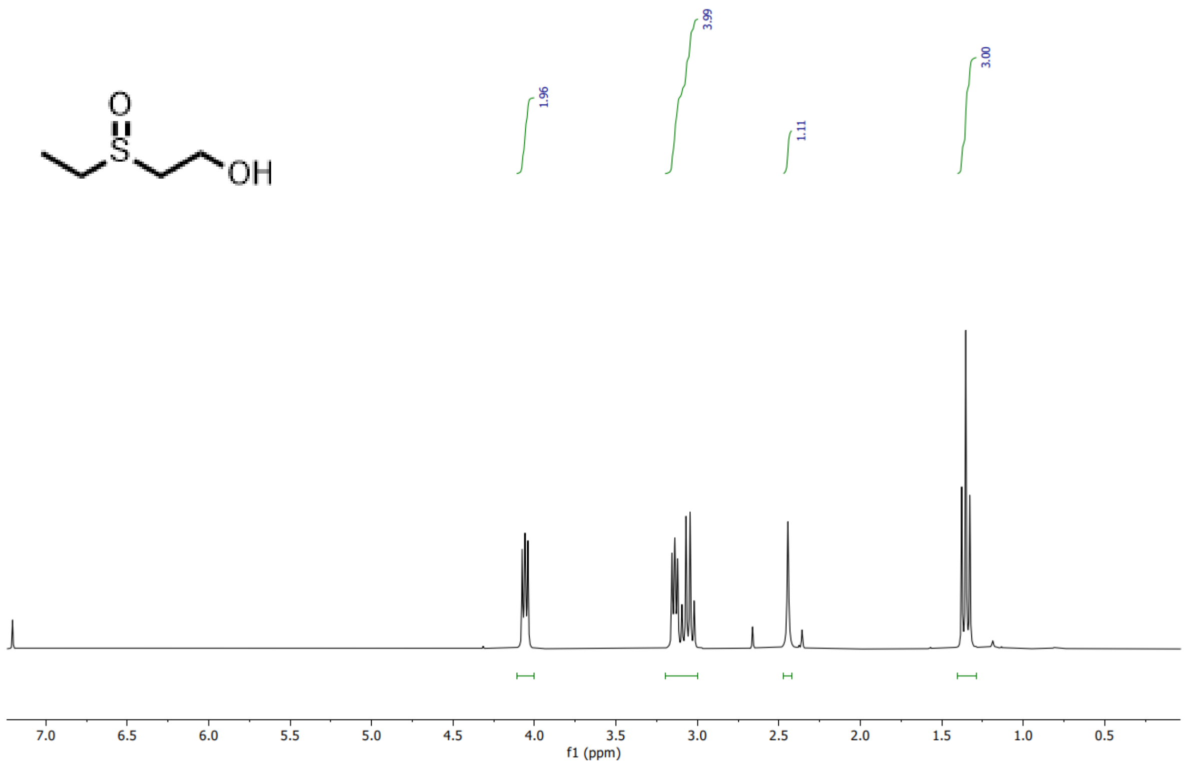

Figure S 34: ^{1}H-NMR spectrum of 2-(ethylsulfinyl)ethan 1-ol.

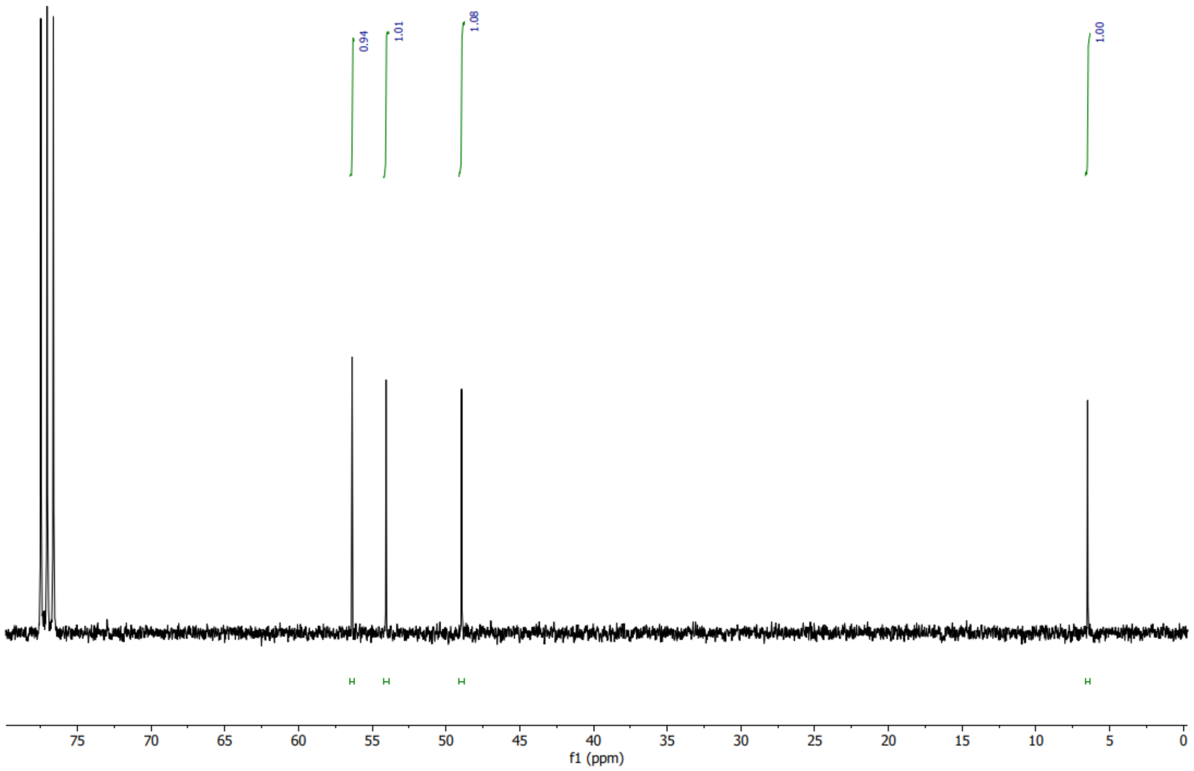

Figure S 35: ^{13}C-NMR spectrum of 2-(ethylsulfinyl)ethan 1-ol.

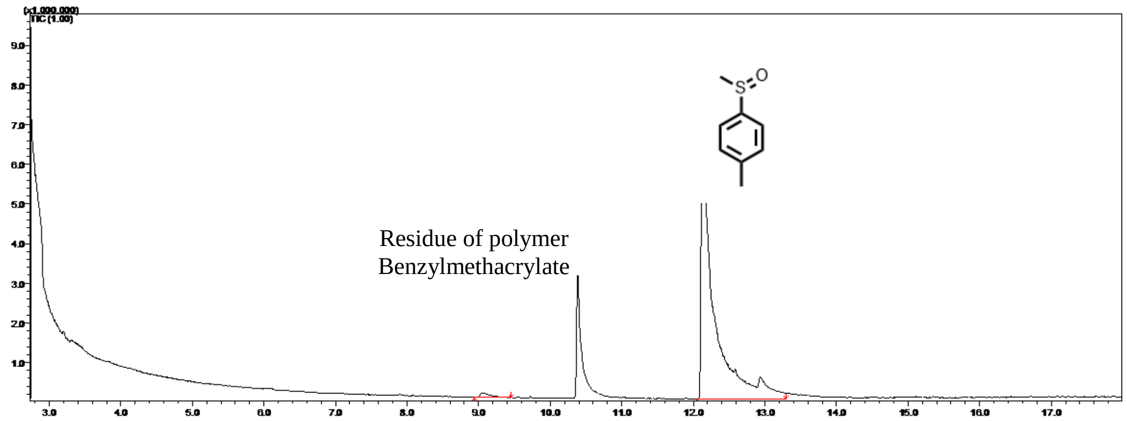

Figure S 36: GC/MS retention time diagram, showing the formation of 1-methyl-4-(methylsulfinyl)benzene and trace amounts of the starting material methyl(p-tolyl)sulfane.

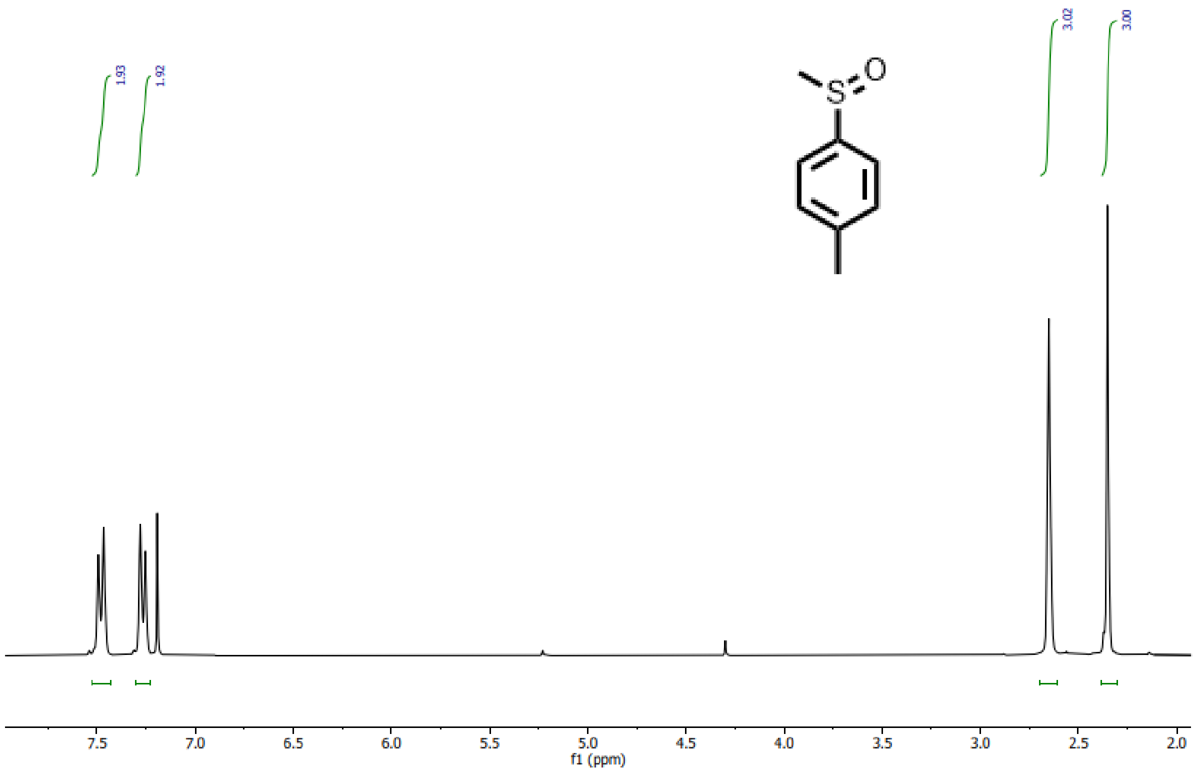

Figure S 37: ^{1}H-NMR spectrum of 1-methyl-4-(methylsulfinyl)benzene.

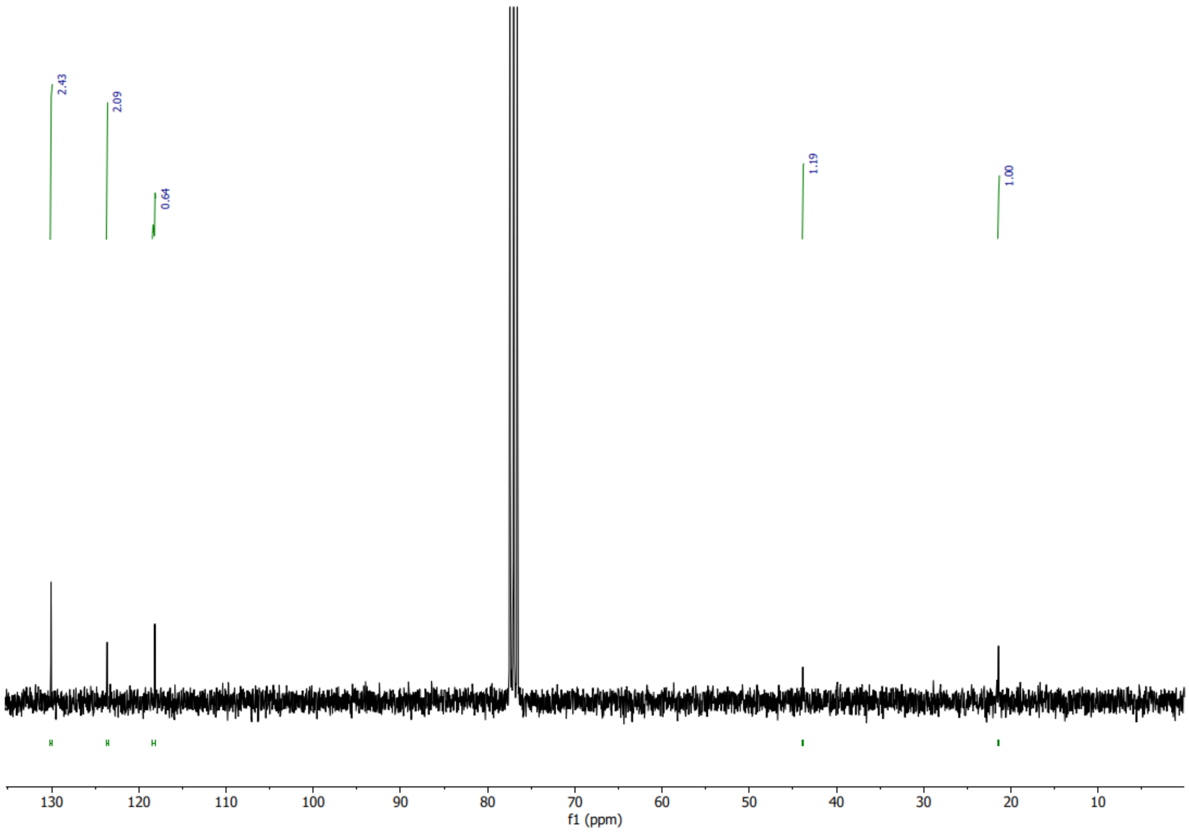

Figure S 38: ^{13}C-NMR spectrum of 1-methyl-4-(methylsulfinyl)benzene.

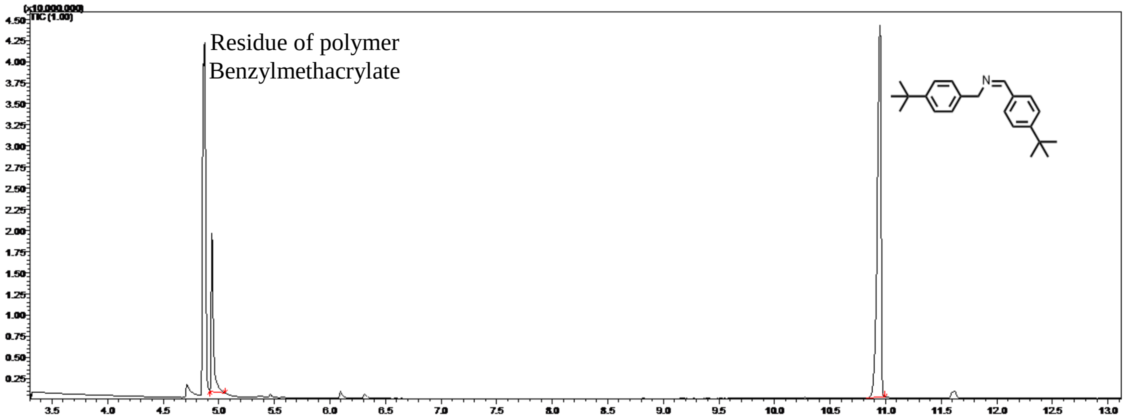

Figure S 39: GC/MS retention time diagram, showing the formation of N-(4-(tert-butyl)benzyl)-1-(4-(tert-butyl)phenyl)methanimine and trace amounts of the starting material (4-(tert-butyl)phenyl)methanamine.

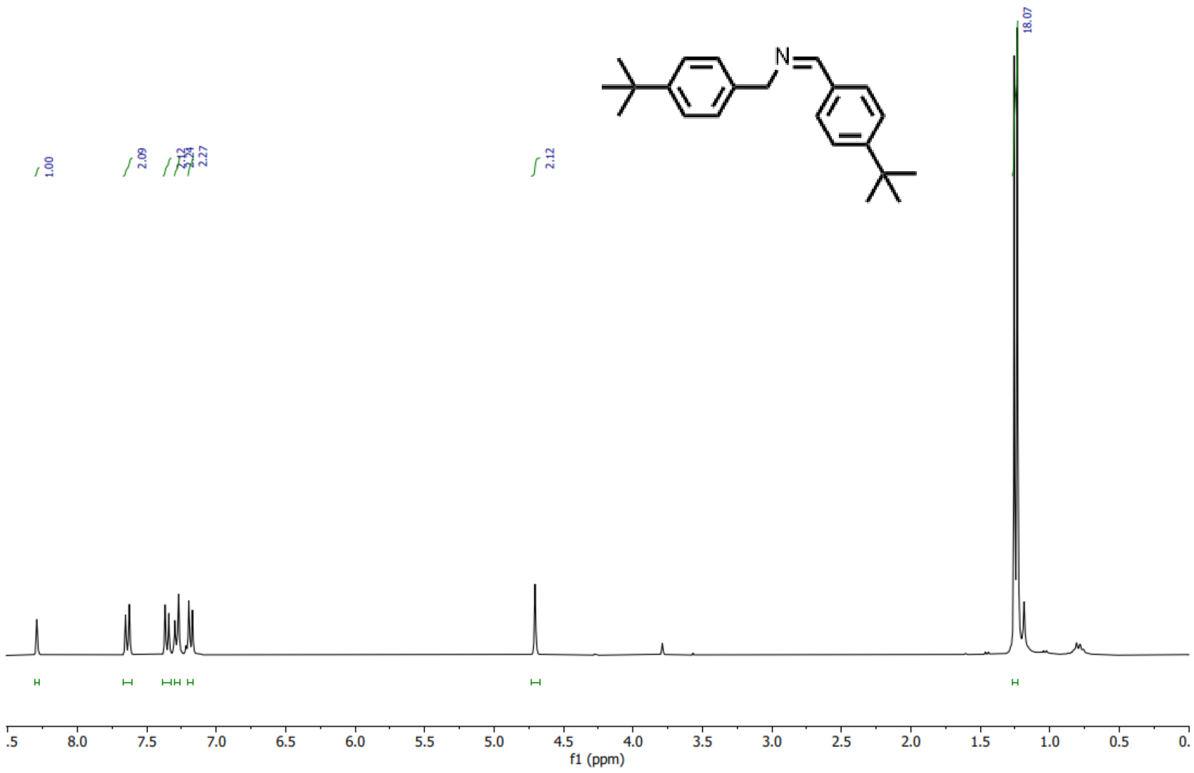

Figure S 40: ^{1}H-NMR spectrum of N-(4-(tert-butyl)benzyl)-1-(4-(tert-butyl)phenyl)methanimine.

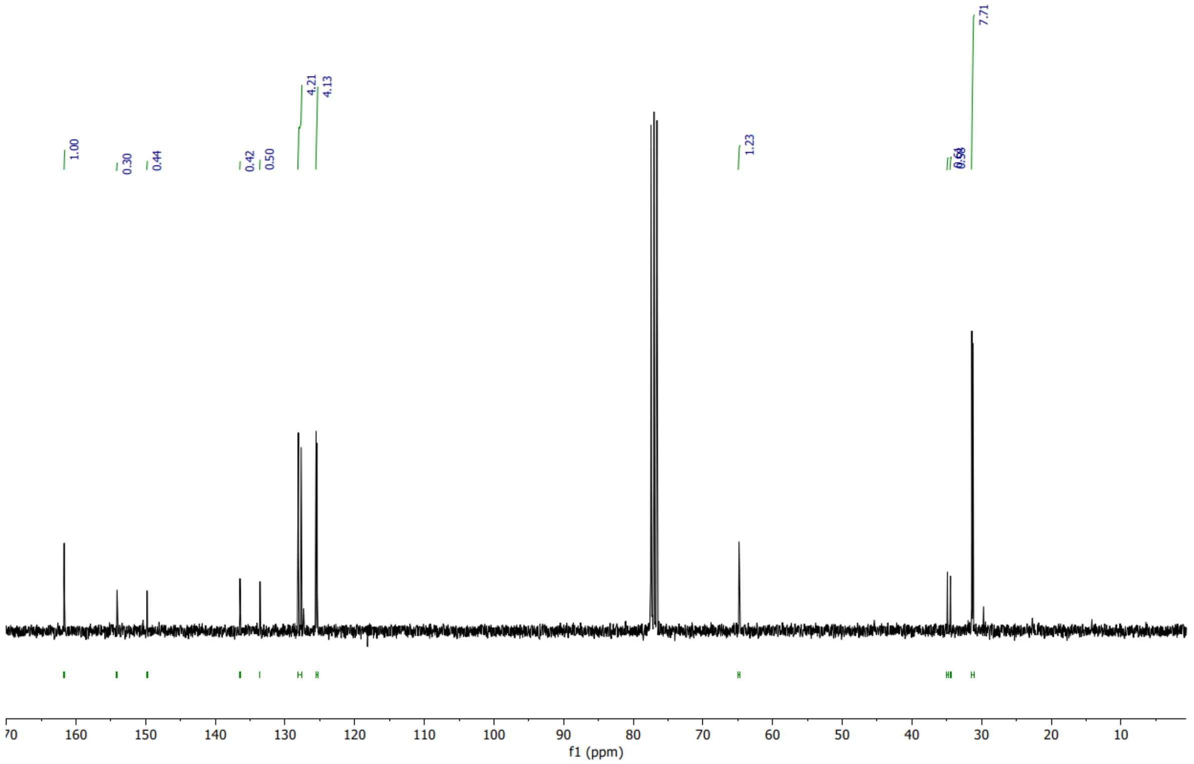

Figure S 41: ^{13}C-NMR spectrum of N-(4-(tert-butyl)benzyl)-1-(4-(tert-butyl)phenyl)methanimine.

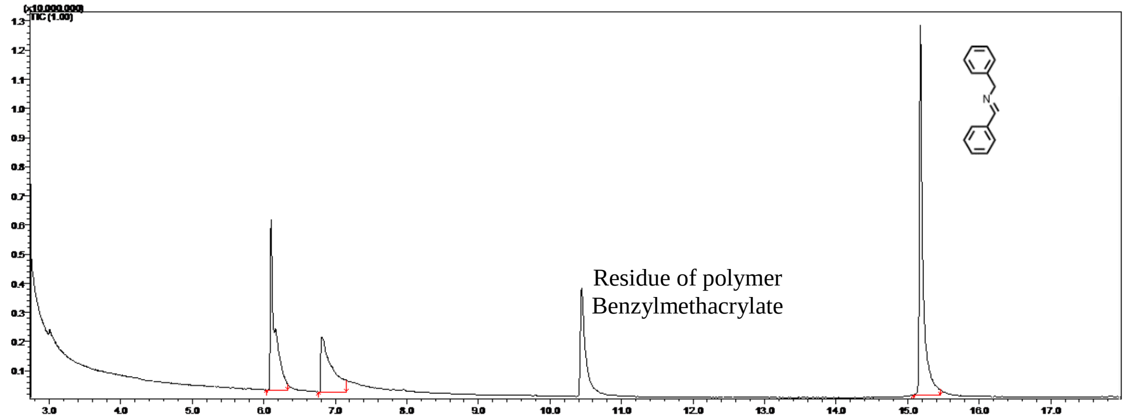

Figure S 42: GC/MS retention time diagram, showing the formation of N-benzyl-1-phenylmethanimine and trace amounts of the starting material benzyl amine.

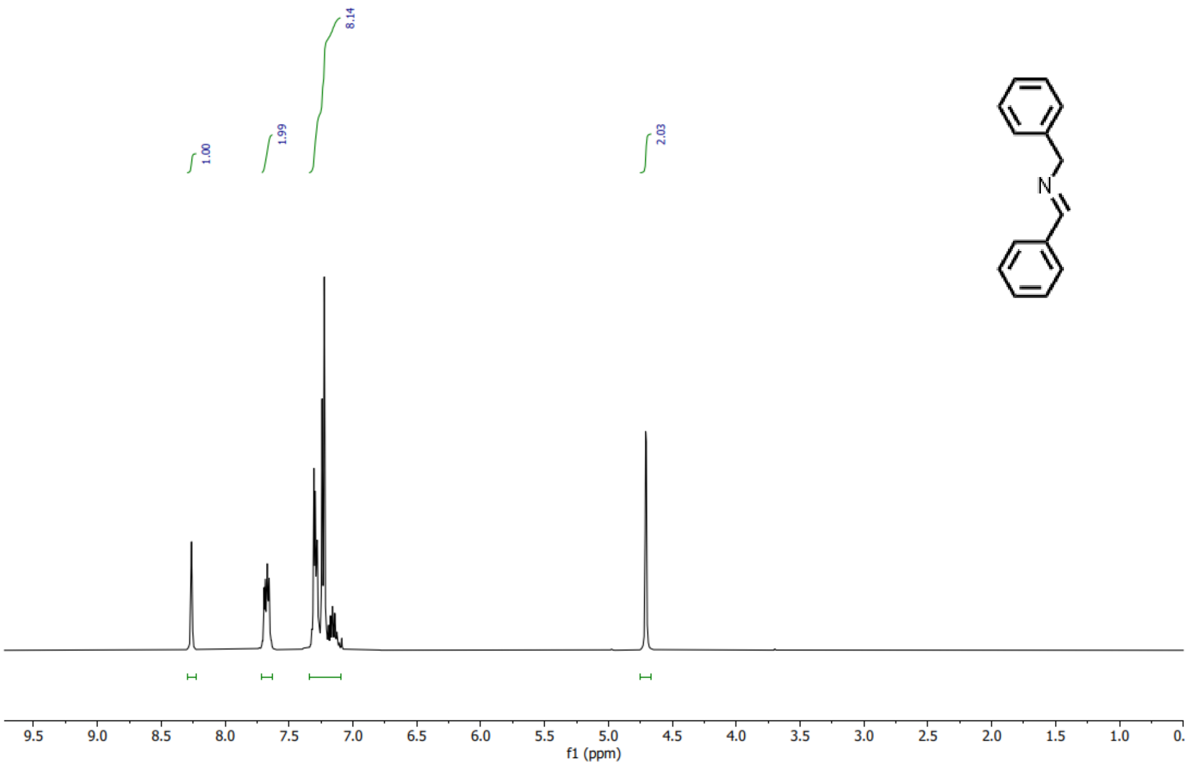

Figure S 43: ^{1}H-NMR spectrum of N-benzyl-1-phenylmethanimine.

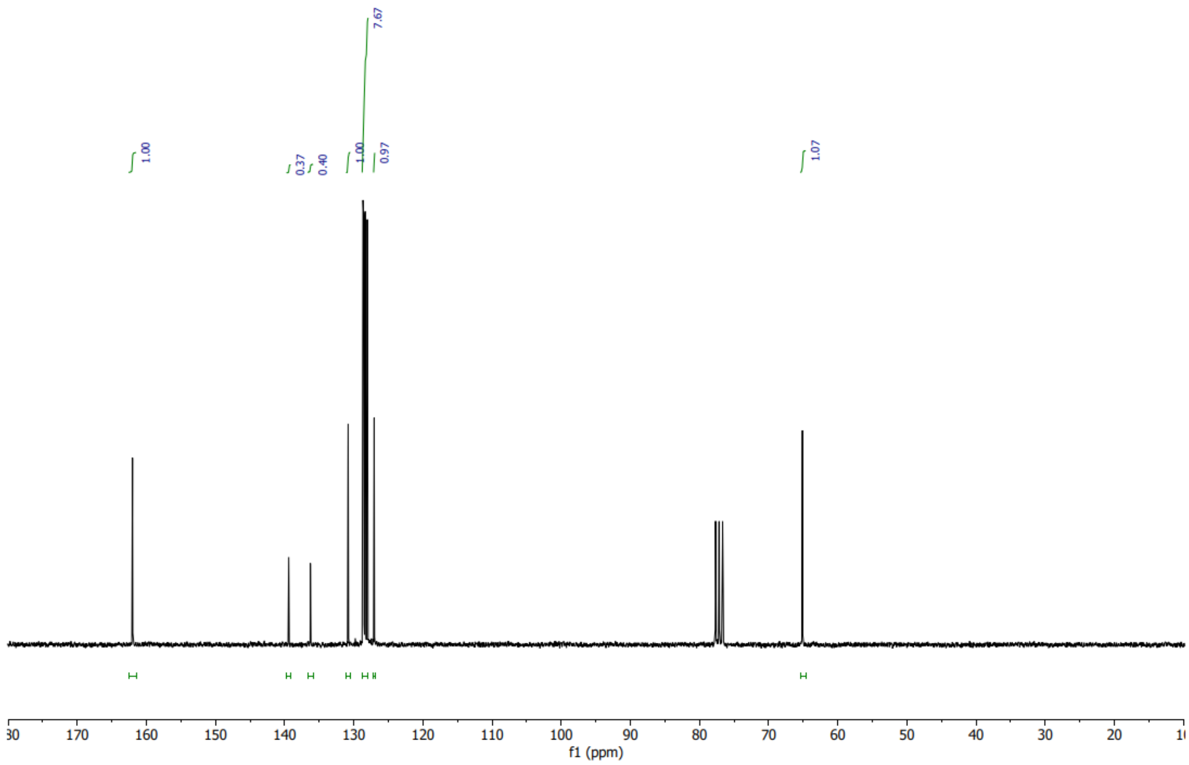

Figure S 44: ^{13}C-NMR spectrum of N-benzyl-1-phenylmethanimine.

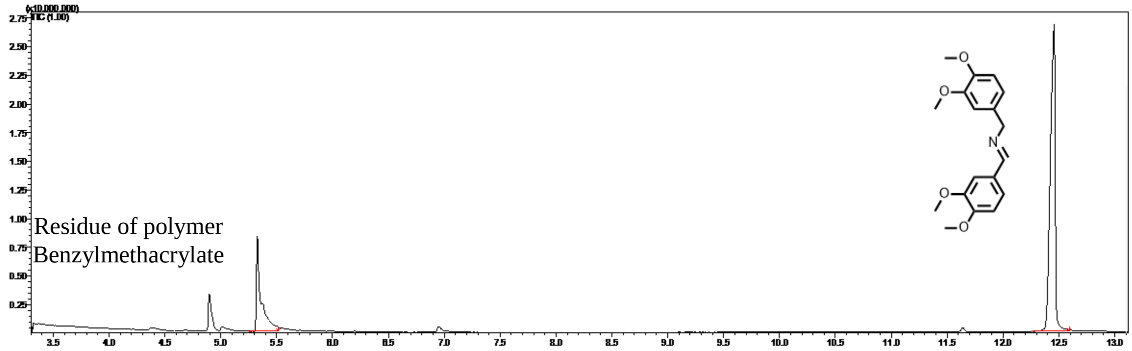

Figure S 45: GC/MS retention time diagram, showing the formation of (3,4-dimethoxyphenyl)methanamine and trace amounts of the starting material N-(3,4-dimethoxybenzyl)-1-(3,4-dimethoxyphenyl)methanimine.

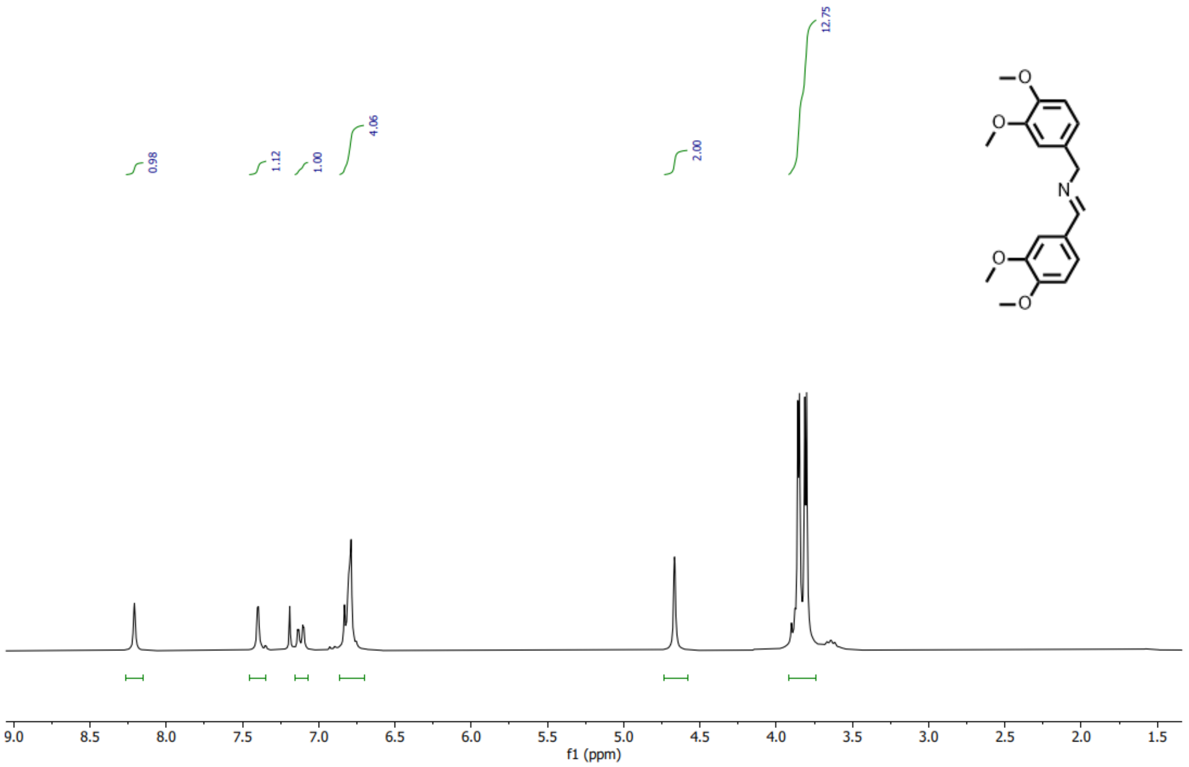

Figure S 46: ^{1}H-NMR spectrum of (3,4-dimethoxyphenyl)methanimine.

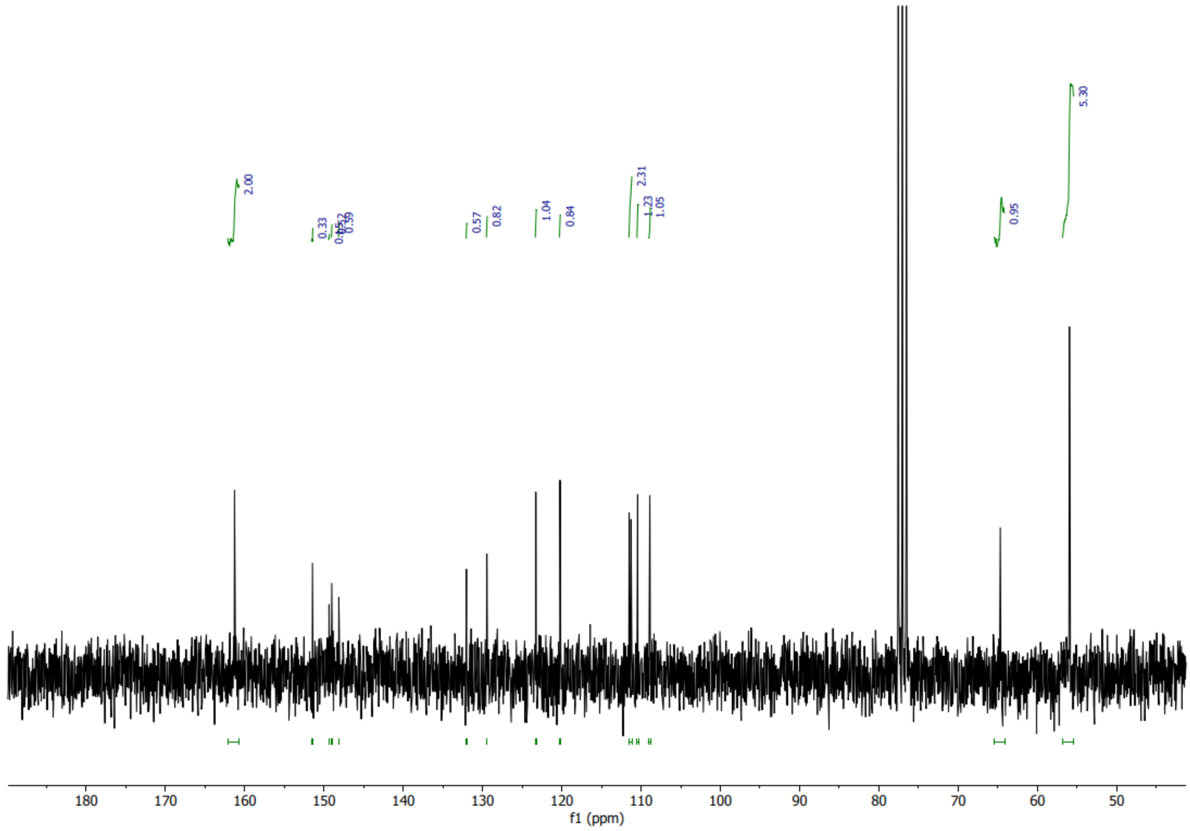

Figure S 47: ^{13}C-NMR spectrum of (3,4-dimethoxyphenyl)methanimine.

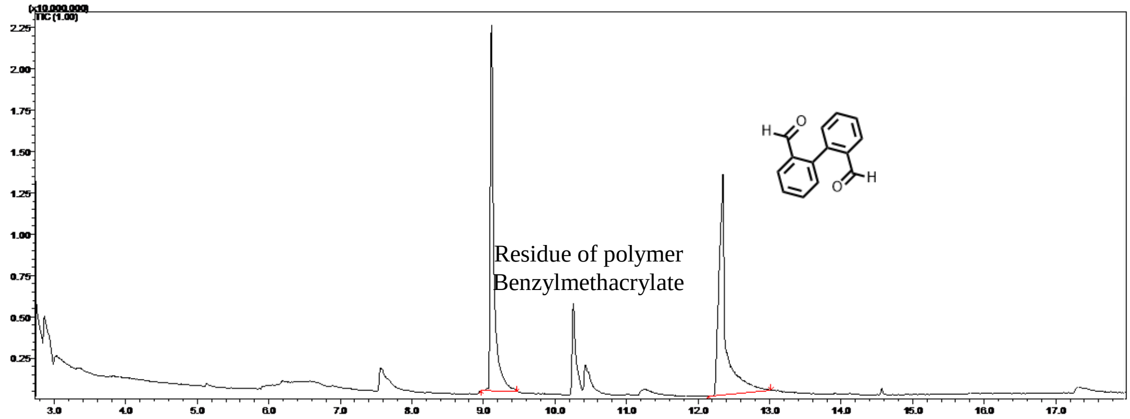

Figure S 48: GC/MS retention time diagram, showing the formation of [1,1'-biphenyl]-2,2'-dicarbaldehyde and trace amounts of the starting material 2-bromobenzaldehyde.

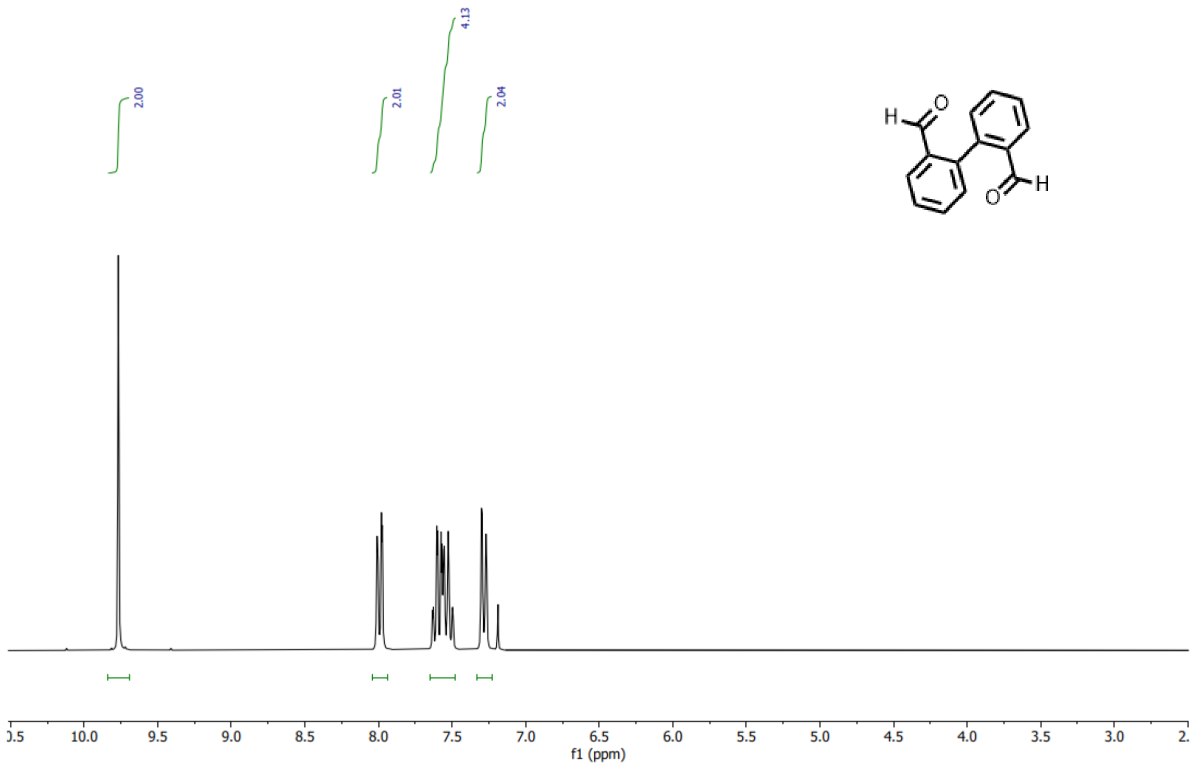

Figure S 49: ^{1}H-NMR spectrum of [1,1'-biphenyl]-2,2'-dicarbaldehyde.

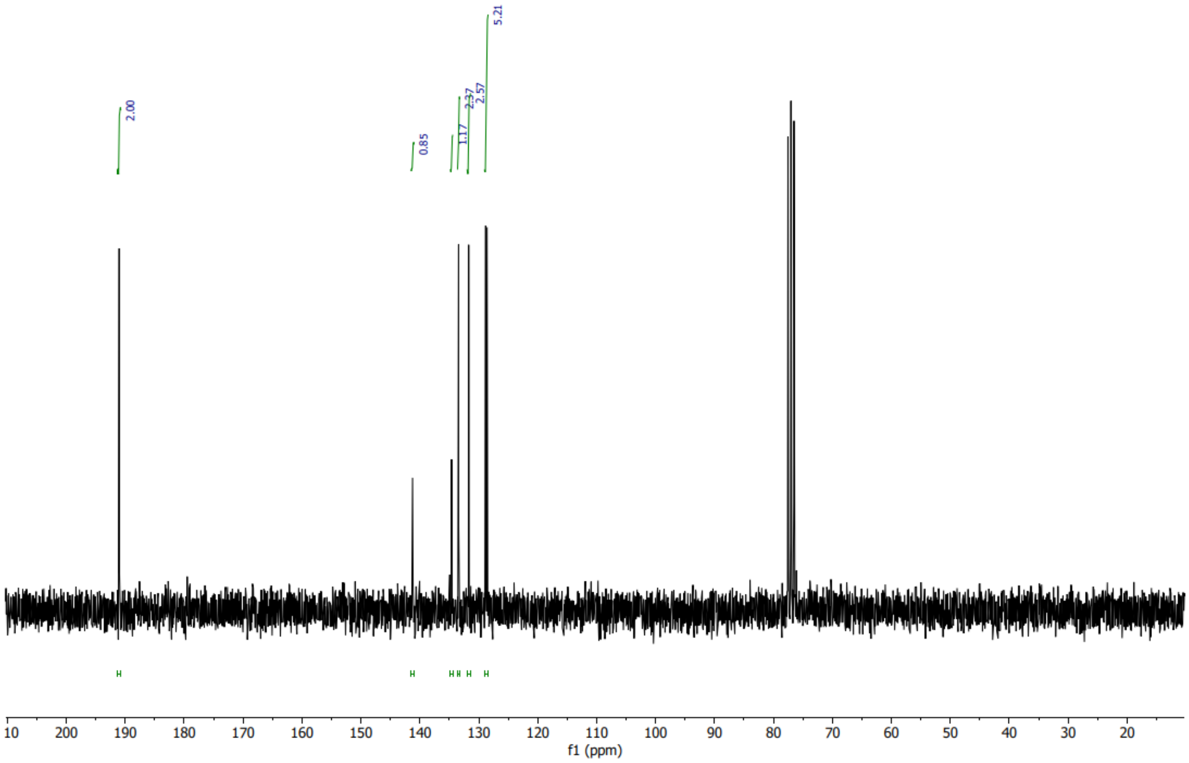

Figure S 50: ^{13}C-NMR spectrum of [1,1'-biphenyl]-2,2'-dicarbaldehyde.

References

[1] Ghose, A. K.; Crippen, G. M. Atomic Physicochemical Parameters for Three-Dimensional-Structure-Directed Quantitative Structure-Activity Relationships. 2. Modeling Dispersive and Hydrophobic Interactions. *J Chem Inf Comput Sci* **1987**, *27* (1), 21-35. DOI: 10.1021/ci00053a005.
